# Solar-driven defluorination via hydroxyl radical spillover for complete mineralization of organofluorine pollutants without fluoride byproducts

**DOI:** 10.1038/s42004-025-01655-3

**Published:** 2025-08-16

**Authors:** Lei Zheng, Jing-Lan Zhang, Zhixin Zheng, Chujie Huang, Yi-Lin Xie, Xu-Bing Li, Wondu Dagnaw Fentahun, Tieyu Wang, Qing-Xiao Tong, Jing-Xin Jian

**Affiliations:** 1 Department of Chemistry, Shantou University, Shantou, PR China; 2 Guangdong Provincial Key Laboratory of Marine Disaster Prediction and Prevention, Shantou University, Shantou, PR China; 3 Key Laboratory of Photochemical Conversion and Optoelectronic Materials, Technical Institute of Physics and Chemistry, Chinese Academy of Sciences, Beijing, PR China

**Keywords:** Pollution remediation, Sustainability

## Abstract

The recalcitrance of fluorinated organic pollutants—featuring robust Csp²-F and Csp³-F bonds—poses critical challenges to aquatic ecosystems due to their extreme persistence and bioaccumulation. Whereas current destruction strategies suffer from high energy consumption and non-selective, here we present a solar-powered mineralization strategy utilizing cerium oxide/mesoporous silica (CeO_2_/mSiO_2_) heterojunction photocatalysts for complete defluorination of organofluorine contaminants, including fluorinated e-waste, fluoro-antibiotics and perfluorinated surfactant. Under visible light irradiation, the optimized 5%CeO_2_/mSiO_2_ achieved 91.1 ± 3.2% octafluorobiphenyl (OFB) and 97.7 ± 2.8% fleroxacin (FLE) degradations within 6 h. Notably, the ‘forever chemical’ perfluorooctanesulfonic acid (PFOS) can be effectively destructed, achieving a maximum of 25.9 ± 2.7% reduction in 5 days under sunshine, outperforming parallel experiments conducted without a catalyst (~0%). This process notably avoids the evolution of fluoride ions. Theoretical calculations reveal that the removal of C-F bonds by photogenerated hydroxyl radical is thermodynamically superior to hydroxyl-mediated defluorination. This work establishes an energy-efficient paradigm for eradicating “forever chemicals” without secondary pollution, advancing sustainable water remediation technologies.

## Abstract (web summary)

Current strategies for the remediation of fluorinated organic pollutants suffer from high energy consumption and secondary pollution risk. Here, the authors present a solar-powered mineralization strategy using cerium oxide/mesoporous silica heterojunction photocatalysts that achieves complete defluorination of different types of organofluorine contaminants, including fluorinated e-waste, fluoro-antibiotics and perfluorinated surfactants.

## Introduction

Since fluorine has the strongest electronegativity in the periodic table and is the second smallest atom after hydrogen, introducing fluorine and fluorine-containing groups into organic molecules can effectively improve their physicochemical properties, such as acidity, lipophilicity and stability^1^. The ubiquitous use of organofluorine chemicals poses a serious globally realistic risk to human health and ecosystems^2,3^. Representative compounds such as perfluorooctanoic acid (PFOA) and perfluorooctane sulfonate (PFOS), listed under the Stockholm Convention on Persistent Organic Pollutants (POPs)^4,5^, demonstrate significant spatial and vertical distribution gradients in the Bohai and Yellow Seas^6^. These contaminants exhibit long-range atmospheric transport potential, aquatic mobility, pronounced bioaccumulation tendencies, and considerable ecotoxicological effects^7^.

Existing organofluorine treatment technologies are mainly divided into two categories: adsorption-separation and degradation-destruction. Activated carbon^8–10^, ion exchange resin^11–13^ and mineral materials^14^ are commonly used as adsorption-separation materials to achieve the enrichment and transfer of organofluorine, but they still face challenges such as difficulty in adsorbent regeneration, low removal rate, low selectivity, and risk of secondary pollution. On the other hand, conventional pyrolysis^15–17^, incineration^18,19^, ultrasonication^20,21^, plasma-based oxidation^22^, electrochemical degradation^23,24^, supercritical water oxidation^25,26^ and ultraviolet-initiated degradation^27–30^ have been developed. However, current destruction strategies have shortcomings, including high energy consumption, harsh conditions, the use of heavy-metal catalysts, and toxic side effects such as fluoride ions-induced emission and contamination^31^. Recently, Brittany Trang et al. reported a mineralization of PFOA through a sodium hydroxide–mediated defluorination pathway, which converted the ‘forever chemical’ PFOA to fluoride ions within 24 h in polar aprotic organic solvents^32^. Undoubtedly, this robust alkali condition is too harsh and dangerous for actual sewage treatment. Leveraging solar energy and the reactivity of C_sp2_-F and C_sp3_-F bonds^33,34^, solar-driven mineralization might offer milder alternatives to address the impending risk of the organofluorine contamination.

Solar-driven degradation is a promising method to actuate efficient cleavage of organofluorine pollutants owing to its merits of facile operation, high efficiency and low secondary pollution^35^. Some previous works reported PFOA was completely degraded into CO_2_ and F^−^ on TiO_2_ photocatalyst under the irradiation of mercury lamp (mainly ultraviolet light)^36^, and the fluorinated liquid crystal monomer (LCM) pollutants were degraded under the ultraviolet/peroxy disulfate treatment^37^. However, these photodegradation systems were based on wide bandgap semiconductors (TiO_2_, In_2_O_3_, Ga_2_O_3_, BiOHP, etc) containing precious metal and heavy metal catalysts^38^, which can only use ultraviolet light (mainly 254 nm) and fail to utilize abundant sunlight^39–41^. Besides, the degradation of organofluorine releases harmful small molecule fragments or F ions, and does not fully realize the mineralization and harmlessness of pollutants. To date, few research has explored the mineralization of organofluorine pollutants containing different hybrid C-F bonds under visible light^32^. Therefore, there is an urgent need to develop visible light-responsive photocatalysts, which should demonstrate exceptional efficacy in circumventing the prohibitive thermodynamic constraints governing C-F bond cleavage in persistent organofluorine contaminants, as well as concomitant advantages in economic feasibility and ecological safety.

**Fig. 1:**
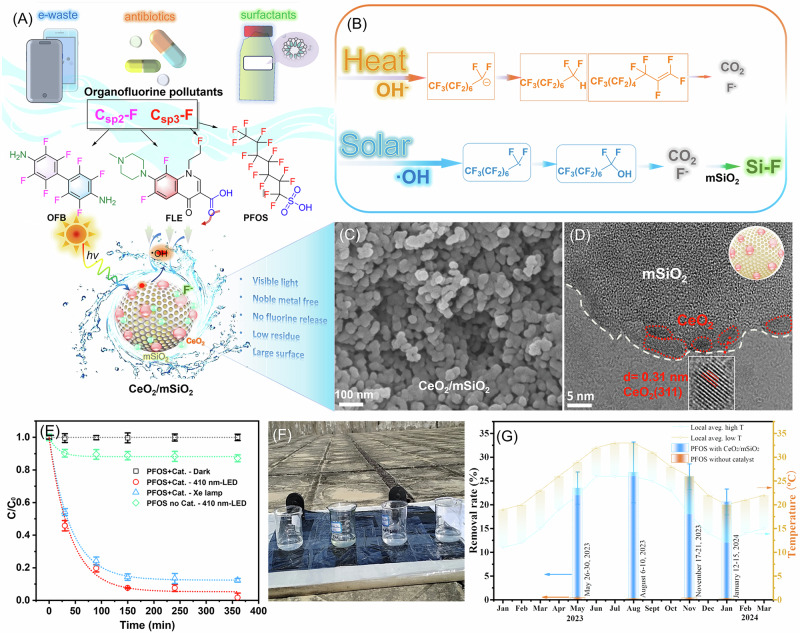
Schematic diagram illustrating the solar-driven mineralization of organofluorine pollutants. **A** Fluorinated e-waste OFB, fluoro-antibiotics FLE and perfluorinated surfactant PFOS were mineralized by CeO_2_/mSiO_2_ photocatalyst. **B** Comparison of the destruction of PFOS *via* photogenerated •OH under solar and hydroxyl-mediated defluorination under heating. SEM (**C**) and HRTEM (**D**) images of CeO_2_/mSiO_2_, showing (311) lattice fringes of CeO_2_. **E** Photodegradation of PFOS under the illuminations of visible light (410-nm LED) and simulated sunlight (Xe lamp, AM1.5 G) in the presence and absence of CeO_2_/mSiO_2_ (2.0 g L^−1^). Photograph of the outdoor sunshine experiment (**F**) and the removal rate of PFOS by CeO_2_/mSiO_2_ catalyst after 5 days illuminations (**G**), along with the local monthly average temperature recorded over a one-year period. Data are denoted as mean ± s.d. (*n* = 3).

Mesoporous silica (mSiO_2_), which is similar to the main components of sand and environmentally friendly materials, has a large specific surface area to enhance the adsorption of organic pollutants and fluoride ion^42,43^. Meanwhile, cerium oxide (CeO_2_) is a strong oxidizing photocatalyst, which can produce highly active hydroxyl radical (•OH) species under visible light irradiation to destroy organic pollutants. Herein, we present a solar-powered mineralization strategy utilizing CeO_2_/mSiO_2_ heterojunction photocatalysts for complete defluorination of organofluorine contaminants. Subsequently, fluorinated e-waste octafluoro-4,4’-biphenyldiamine (OFB)^44–48^, fluoro-antibiotic fleroxacin (FLE)^49–51^, and perfluorinated surfactant PFOS^27^ were selected as representative organofluorine pollutants with typical C_sp2_-F and C_sp3_-F bonds to explore the visible-light removal performance (Fig. 1A). Notably, experimental results and density functional theory (DFT) calculations reveal that the destruction of C_sp2_-F and C_sp3_-F bonds by photogenerated •OH was thermodynamically superior to hydroxyl (OH^−^)-mediated defluorination under heating conditions (Fig. 1B). This work provides a feasible photodegradation technology for sustainable wastewater management in China, especially because emerging contaminants with ecological risks are not given enough attention.

## Results

### Preparation and characterization of CeO_2_/mSiO_2_ nanocomposites

CeO_2_/mSiO_2_ nanocomposites with tunable CeO_2_ loadings (5–80 wt%) were fabricated via the sol-gel method and subsequent hydrothermal reaction (Supplementary Fig. S1). Under alkaline conditions, tetraethyl orthosilicate (TEOS) undergoes hydrolysis to form silicic acid (Si(OH)_4_), while CTAB micelles act as structural templates. Subsequently, silicate species condense on the surface of the micelles, forming a core-shell composite structure with the micelles as the core and silica as the shell. After removing the CTAB template *via* high-temperature calcination, mesoporous SiO_2_ (mSiO_2_) with a high specific surface area is obtained. Furthermore, introducing mSiO_2_ during the in-situ synthesis of CeO_2_ enables the uniform dispersion of cerium salts within the mSiO_2_ matrix. This approach effectively prevents the severe agglomeration of CeO_2_ nanoparticles that typically occurs during high-temperature calcination, thereby preserving their catalytic performance. As evidenced by scanning electron microscopy (SEM), the optimized 5%CeO_2_/mSiO_2_ composite demonstrates monodisperse spherical nanostructures with an average diameter of 50 nm (Fig. 1C). High-resolution transmission electron microscopy (HRTEM) analysis reveals distinct lattice fringes with a d-spacing of 0.31 nm, corresponding to the (311) crystallographic plane of CeO_2_, confirming successful surface decoration of CeO_2_ nanoparticles on the mesoporous silica matrix (Fig. 1D). Energy dispersive X-ray spectroscopy (EDX) elemental mapping (Supplementary Figs. S2, S3) verifies the homogeneous distribution of Ce, Si and O within the composite architecture. X-ray powder diffraction (XRD) patterns (Supplementary Fig. S4) exhibit progressive enhancement of characteristic CeO_2_ diffraction peaks of (111), (200) and (311) (JCPDS 34-0394) with increasing CeO_2_ loading, while pristine mSiO_2_ maintains an amorphous profile. The linear correlation between CeO_2_ content and peak intensity confirms controlled crystallinity modulation through synthesis parameter optimization.

N_2_ physisorption isotherms (Supplementary Fig. S5) demonstrate type-IV hysteresis loops, characteristic of hierarchical mesoporous structures. The 5%CeO_2_/mSiO_2_ composite retains exceptional surface area (884.7 m^2^ g^−1^) and pore volume (1.61 cm^3 ^g^−1^), comparable to bare mSiO_2_ (913.7 m^2^ g^−1^, 1.65 cm^3 ^g^−1^), with bimodal pore size distributions centered at 1.5 and 3.8 nm (micropores) and 30–60 nm (mesopores). Notably, these values surpass conventional SiO_2_ aerogels and nanospheres (Supplementary Table S1)^42,52,53^, attributable to the structural preservation during CeO_2_ integration. The superior performance stems from fundamental structural differences: CTAB-templated mSiO_2_ achieves precisely controlled 2–50 nm mesopores through micelle-directed sol-gel assembly, yielding exceptionally high surface area and uniform pore distribution. In contrast, conventional SiO_2_ aerogels exhibit macropores (tens-hundreds nm) with surface area primarily from nanoscale frameworks rather than optimized mesoporosity. The slight reduction in chiral mSiO_2_‘s surface area versus standard mSiO_2_ arises from chiral dopants perturbing CTAB micelle self-assembly during synthesis, reducing template uniformity. Crucially, our material’s mesopore density provides 3.2-folds more accessible active sites per unit volume than aerogels, explaining the enhanced performance metrics.

UV-Vis diffuse reflectance spectroscopy (Supplementary Fig. S6) reveals a redshifted absorption edge (λ > 430 nm) and reduced optical bandgap (2.86 eV) for 5%CeO_2_/mSiO_2_ compared to pristine mSiO_2_ (3.32 eV), indicating enhanced visible-light harvesting capability. Electrochemical impedance spectroscopy (EIS) Nyquist plots (Supplementary Fig. S7) demonstrate a relatively small semicircle compared with bare mSiO_2_, suggesting that the loading of CeO_2_ enhances the charge transfer efficiency of mSiO_2_. Density functional theory (DFT) calculations the total density of states (TDOS) of CeO_2_/mSiO_2_ shows increased electronic states near the Fermi level (Supplementary Fig. S8). This synergistic enhancement in charge separation and mobility originates from the type-II heterojunction formation at the CeO_2_/mSiO_2_ interface.

### Photocatalytic degradation of organofluorine pollutants

The photocatalytic efficacy of CeO_2_/mSiO_2_ nanocomposites was systematically evaluated using three structurally distinct organofluorine contaminants: fluorinated e-waste octafluorobiphenyl (OFB, C_sp²_-F bonds with amino groups)^44–48^, perfluorooctanesulfonic acid (PFOS, C_sp³_-F bonds with sulfonic acid groups)^27^, and fluorinated antibiotic fleroxacin (FLE, hybrid C_sp²_-F/C_sp³_-F bonds containing carboxylic/amino functionalities)^49–51^ (Fig. 1A, Supplementary Fig. S9). Under visible light irradiation (410 nm LEDs, 50 mW cm^−2^), the optimized 5%CeO_2_/mSiO_2_ achieved 91.1 ± 3.2% OFB degradation within 6 h, significantly outperforming commercial TiO_2_ (52.0 ± 1.9%), pure CeO_2_ (69.6 ± 1.5%), and bare mSiO_2_ (11.2 ± 0.5%). Reaction kinetics analysis revealed pseudo-first-order rate constants (k) of 0.42 h^−1^ (OFB), 0.31 h^−1^ (FLE), and 0.18 h^−1^ (PFOS), correlating with bond dissociation energies of C_sp²_-F (544 kJ mol^−1^) versus C_sp³_-F (~452–489 kJ mol^−1^). Zeta potential measurements demonstrated pH-responsive surface charge modulation of CeO_2_/mSiO_2_, ranging from +0.68 mV (pH 3.3) to −1.74 mV (pH 11.0) (Supplementary Fig. S10). This electrostatic complementarity drives substrate-specific adsorption: OFB (pKa > 9) exhibited enhanced adsorption in alkaline condition *via* amino-CeO_2_ coordination, while PFOS (pKa < 1) achieved enhanced adsorption in acidic solution through sulfonate-silica interactions (Supplementary Fig. S11). FLE’s amphoteric nature enabled dual-mode adsorption across pH 5-9, maximizing degradation efficiency (97.7 ± 2.8%).

Comparative experiments were conducted under various illumination conditions: darkness, monochromatic visible light (3 W 410-nm LEDs, ~30 mW cm^−2^), and simulated sunlight (Xenon lamp with AM 1.5 G filter, 100 mW cm^−2^). In the dark, the catalyst has no degradation effect on PFOS (Fig. 1E), despite CeO_2_ being known for its strong oxidation ability to destroy hydrocarbons. However, in the presence of the CeO_2_/mSiO_2_ catalyst and light irradiation, whether from the 410-nm LED or simulated sunlight, the removal rate of PFOS exceeded 80% within 150 min (Fig. 1E), the removal rate of OFB exceeded 90% within 150 min, and FLE exceeded 90% within 50 min (Supplementary Fig. S12). For a horizontal comparison, PFOS, with its highly stable C_sp3_-F bonds, is the most difficult to degrade (well-known as ‘forever chemical’), while FLE with C-H bonds is the easiest to degrade. In the absence of catalyst, the degradation effect of organofluoride was significantly inhibited. Additionally, the impact of different anions and cations on organofluorine degradation was investigated (Supplementary Fig. S13). Among common anions, the CO_3_^2−^ anion inhibits the degradation of OFB and FLE, while cations such as Na^+^, K^+^ and Ca^2+^ have negligible effects on the degradation of organofluorides.

Solar-driven mineralization of organofluorine contaminants under outdoor illumination was carried out over a one-year period (Fig. 1F), with parallel experiments in May, August, November, and January, (9:00 a.m. to 4:00 p.m, 7 h per day). At the measurement site (Shantou, Guangdong, China; 23.3541°N, 116.6820°E), solar irradiance reached 58.5 mW cm^−2^ at 12:30 local time. Given that PFOS exhibits an extremely slow natural degradation rate (half-life of 4.7)^54^, no significant photolysis occurred within 5 days of direct outdoor sunlight exposure. In contrast, after introducing the CeO_2_/mSiO_2_ photocatalyst, 26.8 ± 6.3% of PFOS underwent destruction within 5 days under sunlight illuminations, and the degradation efficiency fluctuated slightly with season and temperature (Fig. 1G). To our knowledge, this is the first report on the efficient removal of PFOS in outdoor environments by sunlight, which is expected to be applied to the environmental treatment of organofluorine pollutants. Additionally, 83.5 ± 2.1% of OFB and 97.7 ± 2.8% of FLE underwent photodegradation under outdoor sunlight for one-day irradiation (7 h), which was a 2.58- and 1.51-fold enhancement of the photolysis without a catalyst (Supplementary Fig. S14). This solar-driven approach achieves degradation efficiency comparable to conventional advanced oxidation processes (AOPs) such as electrochemical oxidation, Fenton-based methods, plasma treatment, and sonochemical degradation (Supplementary Table S2), while operating under milder conditions suitable for long-term remediation of perfluorinated compounds in outdoor environments.

**Fig. 2:**
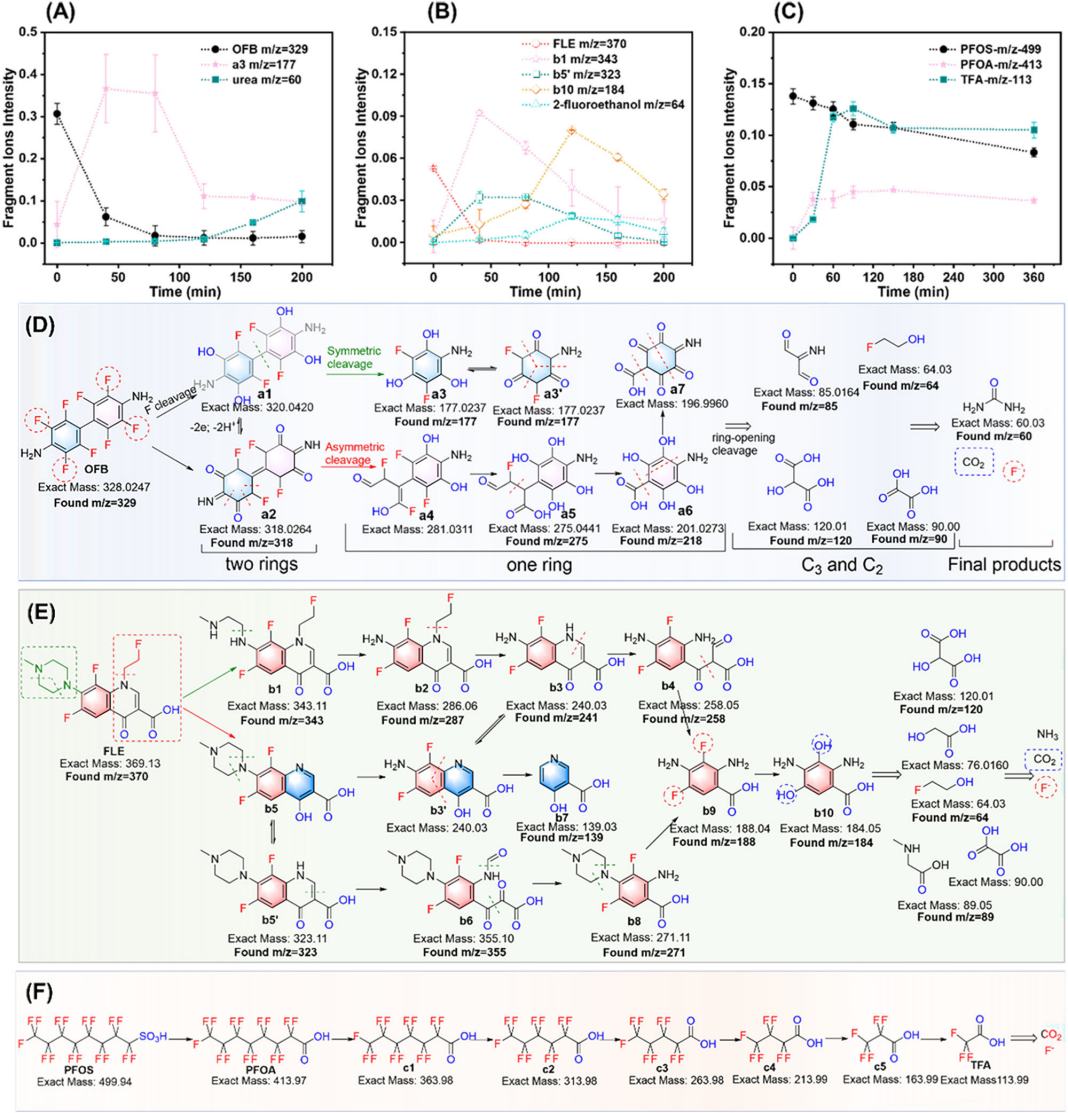
Detection of intermediate products and proposed degradation pathways. The ion fragments intensities of OFB (**A**), FLE (**B**) and PFOS (**C**) during photodegradation by the CeO_2_/mSiO_2_ catalyst, and the rational degradation paths incorporating intermediates detected by LC-MS spectrometry and GC analysis for OFB (**D**), FLE (**E**) and PFOS (**F**), respectively. Data are denoted as mean ± s.d. (*n* = 3).

The intermediates and final products of organofluorine photodegradations were identified using liquid chromatography-mass spectrometry (LC-MS) and gas chromatography (GC) analysis (Fig. 2, Supplementary Figs. S15–S19). As shown in Fig. 2A, the signal changes of each ion fragments of OFB were monitored during 200 min of continuous illumination to elucidate its photodegradation pathways. The signal peak of OFB at m/z 329 vanished completely after 40 min of illumination (Supplementary Fig. S15A). Concurrently, the signal of a2 at m/z 318 disappeared at a retention time of 6–8 min after 40 min of illumination, but reappeared at a retention time of ~2 min, then gradually weakened and ultimately disappeared within 200 min (Supplementary Fig. S15B). Similar trends were observed in the figures of m/z 275, 218, 177, 120 and 90, which exhibited new peaks at retention times of 0.77, 5.8–6.2, 6–7, 0.68 and 0.71 min, respectively (Supplementary Fig. S15C–G). Integrating molecular weight and retention time, the ion peak signals of m/z 120, 90, 85 and 64 were identified as C_3_ or C_2_ intermediates resulting from OFB photodegradation (Supplementary Fig. S15F–I). More intriguingly, after 160 min of illumination, a fragment signal peak of m/z 60 suddenly increased at the retention time of 0.74 min, which was confirmed to be urea by comparison with a standard sample (Supplementary Fig. S15J). Additionally, the indophenol blue method was employed to track the degradation of OFB, ultimately yielding 0.1623 μg mL^−1^ of ammonia (Supplementary Fig. S16), while GC analysis confirmed that OFB photodegradation ultimately produced CO_2_ (Supplementary Fig. S17A). Therefore, the intermediates of ammonia and CO_2_ could be transformed into urea under continuous illumination.

The decomposition of FLE is anticipated to initiate from the functional groups at either the N-substituted piperazine or N-substituted pyridine, which can be divided into two pathways and yield intermediates of b1–b10 with m/z of 343, 286, 240, 258, 323, 355 and 271, respectively (Fig. 2E). As illustrated in Fig. 2B, the time-varying signals confirm that FLE decomposed to b1 and b5’ within 40 min of illumination (Supplementary Fig. S18). Subsequently, they transformed into benzene or pyridine derivatives with m/z of 188, 184 and 139, followed by smaller organic molecule fragments of oxalic acid and N-methyl amino acetic acid. Notably, the detected pyridine derivative intermediate of b7 with m/z of 139 has not been previously mentioned in the reported degradation mechanism of FLE^49,51,55^. Due to the low electron cloud density of the pyridine structure, b7 intermediate is more resistant to oxidants than other benzene derivatives and is challenging to degrade completely. Ultimately, FLE was decomposed into NH_3_ (0.5122 μg mL^−1^) (Supplementary Fig. S16C) and CO_2_ (Supplementary Fig. S17B).

The degradation of PFOS initiates with the elimination of the sulfonic acid group to form PFOA, followed by the decarboxylation-hydroxylation-elimination hydrolysis pathway to shorten the carbon chain (Fig. 2F). Under continuous illumination, intermediate species with m/z of 413, 263, and 113 were produced, which are attributed to PFOA, perfluoropentanoic acid (PFPeA), trifluoroacetic acid (TFA), respectively (Supplementary Fig. S19). Within 360 min of illumination, the concentration decay of PFOS and the intermediates of PFOA and TFA were statistically analyzed, confirming that PFOS was continuously converted into PFOA and eventually reduced the carbon chain to TFA (Fig. 2C). Additionally, the final product of CO_2_ was detected by GC analysis (Supplementary Fig. S17C). The total organic carbon (TOC) variations in the CeO_2_/mSiO_2_ catalyst systems with OFB (11.4 μM, room temperature saturated solution), FLE (50 μM), and PFOS (80 μM) were measured before and after light irradiation. Initially, their TOC values were 2.40, 9.43, and 7.11 mg L^−1^, close to theoretical values. After prolonged irradiation, the TOC values decreased to 1.5–1.8 mg L^−1^, with FLE and PFOS showing TOC reductions of 80.2% and 74.0%, reflecting the complete degradation of these organic fluorine pollutants (Supplementary Fig. S20).

### DFT calculations of C_sp2_-F and C_sp3_-F cleavage

Radical quenching experiments and electron paramagnetic resonance (EPR) analysis were conducted to elucidate the active species involved in the photodegradation of organofluorine. Upon illumination, the photogenerated electron (e^−^) and hole (h^+^) are transferred to the surface of CeO_2_/mSiO_2_ semiconductor, where e^-^ can be captured by O_2_ to form superoxide ion (•O_2_^−^) and h^+^ can be trapped by water to produce hydroxyl radical (•OH). In the EPR measurements, characteristic signals of •OH and •O_2_^−^ were detected on the CeO_2_/mSiO_2_ catalyst under visible light irradiation, with intensities that increased alongside the extension of irradiation time (Supplementary Fig. S21). After purging argon to remove O_2_, the CeO_2_/mSiO_2_ photocatalyst could still degrade OFB, FLE and PFOS under irradiation, thus excluding •O_2_^-^ as an indispensable active species for organofluorine degradation (Supplementary Fig. S22). The photodegradation of organofluorine pollutants was significantly reduced by adding reagents to quench •OH and h^+^, indicating that •OH plays a crucial role in the photodegradation process (Supplementary Fig. S23). DFT calculation results reveal the spatial structure and electrostatic potential (ESP) distributions of OFB, FLE and PFOS (Supplementary Fig. S24), in which the carbon atoms of the C-F bonds are in a charge-deficient state and are therefore more susceptible to attack by hydroxyl radicals or hydroxide ions.

**Fig. 3:**
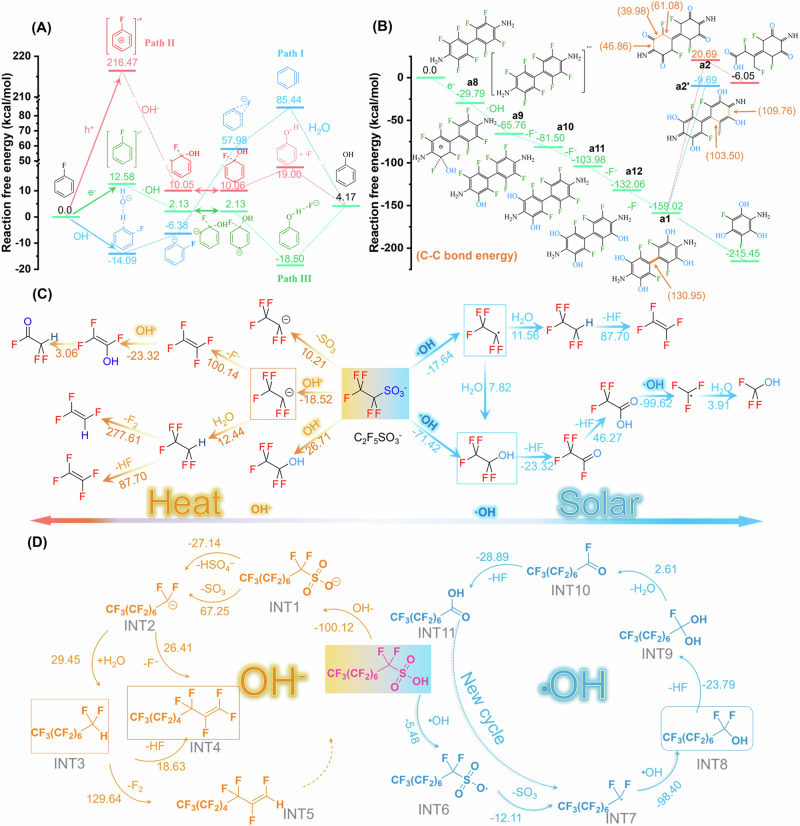
Proposed defluorination mechanisms for C_sp2_-F and C_sp3_-F bonds by OH^−^ or •OH. **A** Energy profiles of three paths for Ph-F conversion to Ph-OH. **B** DFT calculated free energy change when OFB gradually eliminates C_sp2_-F bonds, with specific C-C bond energies in brackets for the intermediates of a1, a2 and a2’. Degradation of PFeSA (**C**) and PFOS (**D**) by OH^−^ under heating and •OH under solar irradiation, with the predicated intermediates and their free energy changes (kcal mol^−1^).

To further elucidate how active species participate in the cleavage of C_sp2_-F bonds, fluorobenzene (Ph-F) was employed as the primary computational model. The feasibility of defluorination through nucleophilic elimination (Path I), oxidation pathways (Path II), and reduction pathways (Path III) was assessed using DFT theoretical calculations (Fig. 3A, Supplementary Fig. S25). Paths I and II are thermodynamically extremely difficult to occur C_sp2_-F bond breaking due to the high-energy barrier of benzyne intermediate (85.44 kcal mol^−1^) and cationic radical (+216.47 kcal mol^−1^). In contrast, the energy barrier for PhF to gain electrons to form anionic radicals in Path III is only +12.58 kcal mol^−1^, and the subsequent reaction with •OH is also a thermodynamically favored process. After the introduction of the strong electronegative fluorine on the conjugated aromatic ring, the π electron cloud is reduced, which facilitates electron acceptance. Consequently, the elimination of C_sp2_-F on the aromatic ring can be achieved by capturing photogenerated electrons, then reacting with •OH and dissociating fluoride ions. Afterward, the defluorination of OFB follows a similar Path III, losing four F atoms at the ortho-positions of the amino groups to form a1 intermediate, which is thermodynamically preferred, and the product was confirmed in LC-MS (Fig. 3B). In contrast, the fluorine atoms at the meta-positions are inert due to their significant steric hindrance. Subsequently, the a1 intermediate could undergo a C-C bond break (bond energy 130.95 kcal mol^−1^) to produce one-ring a3 intermediate, or lose electrons to form a2 intermediate with quinone structures and followed by C-C bond cleavage to form one-ring a4 intermediate (Supplementary Fig. S26). By comparing the C-C bond energies, the destruction of the benzene ring structure via the quinone intermediate is the most thermodynamically favorable path (Supplementary Table S3). Consequently, organofluorine pollutions in e-waste containing C_sp2_-F bonds could be activated by receiving photogenerated electrons and reacting with •OH to form phenolic hydroxyl structures, which can then be oxidized to carbonyl-containing quinone structures and gradually dissociate into small molecular fragments.

The cleavage of C_sp3_-F was analyzed using perfluoroethane sulfonic acid (PFeSA) as the primary computational model. According to the recent work in Science^32^, heating PFOA and NaOH in polar aprotic organic solvents could effectively remove C_sp3_-F *via* hydroxyl-mediated defluorination. Here, the destruction of C_sp3_-F in perfluorinated compounds by heated OH^-^ and photogenerated •OH was compared (Fig. 3C, Supplementary Fig. S27). Under heating conditions, the direct removal of sulfur trioxide (SO_3_) or the nucleophilic substitution of SO_3_^−^ by OH^−^ are thermodynamic energy-consuming processes. A plausible pathway is to produce an alkyl carbon anion CF_3_CF_2_^−^ and a hydrogen sulfate anion (HSO_4_^−^), with a free energy change of −18.52 kcal mol^−1^, which represents a thermodynamically favorable reaction pathway. However, this carbon anion encounters an energy barrier exceeding 100 kcal mol^−1^ when it directly releases an F^−^ to form an olefin structure. Then, it tends to form an extremely stable CF_3_CF_2_-H structure by interacting with the solvent water molecule, thereby significantly hindering the subsequent defluorination process. Conversely, the photogenerated •OH could react with PFeSA to produce a carbon-centered radical CF_3_CF_2_• or an alcohol of CF_3_CF_2_OH with free energies of −17.64 and −71.42 kcal mol^−1^, respectively, which are thermodynamically feasible reactions. Furthermore, the α-difluorool (-CF_2_OH) structure rapidly and spontaneously hydrolyzes to acyl fluoride (-COF) and subsequently to carboxylic acid (-COOH), releasing two HF molecules.

As depicted in Fig. 3D, the degradation of PFOS by OH– and •OH under heating and illumination conditions was compared. In the heating alkaline solution, the sulfonate anion (INT1) formed by PFOS dissociation is attacked by OH^−^, resulting in the production of HSO_4_^−^ and a carbon anion intermediate (INT2). However, INT2 tends to further evolve into superhydrophobic and chemically stable intermediates INT3, INT4 and INT5, making subsequent defluorinations difficult to proceed (Supplementary Fig. S28). In contrast, PFOS can be directly converted into sulfonate radicals (INT6) by photo-generated •OH, and then lose the SO_3_ fragment to form a carbon radical •CF_2_R (ITN7), and subsequently react with •OH to form an intermediate (INT8) with an active RCF_2_OH group. The free energy changes for these continuous reaction steps are −5.48, −12.11 and −98.40 kcal mol^−1^, respectively, indicating thermodynamically favorable processes. Subsequently, the α-difluorool (-CF_2_OH) group undergoes hydrolysis reactions to form geminal diol (INT9), acyl fluoride (INT10) and finally carboxylic acid groups (INT11). Following this, the generated perfluorooctanoic acid (PFOA) can be gradually reduced by a difluoromethylene unit (CF_2_) using a similar reaction path, with free energy changes of −186~−188 kcal mol^−1^ (Supplementary Fig. S29). The free energy changes of the stepwise chain-shortening reactions exceed the free energy change of the initial degradation of PFOS to PFOA, confirming the super stability and difficulty in initiating the destruction of PFOS. In conclusive, under mild illumination conditions, photogenerated •OH offers a favorable mechanistic pathway for the destruction of C_sp3_-F bonds.

The fluoro-antibiotics of FLE is speculated to have three parallel degradation pathways: deflourination of C_sp2_-F bonds, piperazine ring cleavage, and aromatization involving N-fluoroethyl elimination. The plausibility of the intricate degradation path of FLE was confirmed by energy barrier and bond energy calculations, with the lowest starting energy barrier (2.15 kcal mol^−1^) for the deflourination of C_sp2_-F bonds (Supplementary Fig. S30). Similar to the previous C_sp2_-F bond destruction process, the aromatic ring of FLE accepts an electron and then reacts with •OH to produce phenolic hydroxyl structure, achieving defluorination of C_sp2_-F bonds, which is thermodynamically permitted. In the second pathway, piperazine undergoes oxidative ring opening, in which the nitrogen atom in piperazine loses electrons to form an imine cation, and then hydrolyzes, leading to the breaking of the C-N bond. This pathway has been speculated and mentioned in the literature^47,49,51,55^, even though the reaction energy barrier for the initial electron loss process is high. In the third pathway, FLE eliminates the β-fluoroethyl group and then rearranges protons to form a benzopyridine skeleton structure with a low free energy change of 2.15 kcal mol^−1^. These paths are intertwined and occur simultaneously or successively, allowing FLE to be degraded into various small fragments, as confirmed by LC-MS analysis. Considering the C-N bond energies in N-ethyl and piperazine, the C-N bond energy in ethyl is lower (100 kcal mol^−1^) than that of C-N bonds in piperazine (151.58 and 141.96 kcal mol^−1^) (Supplementary Table S4), making the aromatization pathway feasible, which is consistent with the results of LC-MS analysis. After aromatization, the C-N bond energy of piperazine decreases to 138.14 and 138.03 kcal mol^−1^, which is beneficial to the subsequent C-N dissociation process. In addition, the destruction of 2-fluoroethanol fragment by •OH is thermodynamically favorable.

### Defluorination of organofluorine

**Fig. 4:**
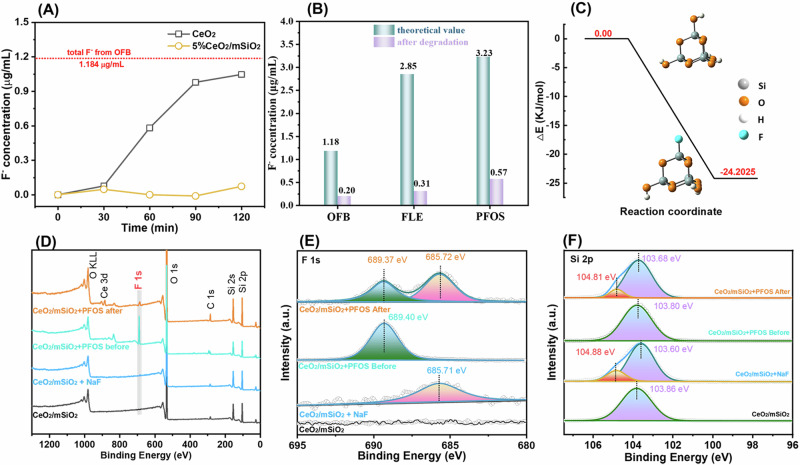
Exploration of the adsorption of F^-^ by CeO_2_/mSiO_2_ catalyst after photodegradation. **A** The concentration change of F^−^ during the photodegradation of OFB. **B** Theoretical and detected F^−^ concentrations after completely photodegradation of OFB (11.4 μM), FLE (50 μM) and PFOS (10 μM) with irradiation times of 2, 2 and 6 h, respectively. **C** Free energy change of the conversion of Si-OH into Si-F. XPS survey (**D**) and high-resolution Si 2p (**E**) and F 1s (**F**) of CeO_2_/mSiO_2_ before and after degradation of PFOS, taking CeO_2_/mSiO_2_ soaked in NaF solution as a reference.

Unexpectedly, the concentration of F^−^ in the solution did not increase significantly after the complete photodegradation of OFB, FLE and PFOS using CeO_2_/mSiO_2_ photocatalyst (Fig. 4), which is advantageous compared to the reported systems that release F^−^ and CO_2_^41,56^. In the comparative experiments, the concentration of F^−^ increased to 1.05 μg mL^−1^ after 120 min of illumination using CeO_2_ as photocatalyst, which was close to the theoretical value (1.18 μg mL^−1^) of organofluorine pollutants (Fig. 4A). This result indicates that the generated F^−^ is adsorbed by the CeO_2_/mSiO_2_ catalyst, as mesoporous SiO_2_ is a potential adsorbent for removing free F^−^^42,43^. EDS mapping shows that the F element was primarily distributed on the edges of the catalyst particles (Supplementary Figs. S31–S33). Notably, the contents of F element in the CeO_2_/mSiO_2_ catalysts increased to 1.55%, 2.48% and 2.20% after the photodegradation of PFOS, OFB, FLE, respectively. In FTIR spectra, the characteristic signals of organofluorine, attributed to the stretching vibration (ν) of C-F, N-H, aromatic ring, C-N, and the bending vibration (δ) of N-H, are obviously attenuated or even disappeared after illumination. Interestingly, CeO_2_/mSiO_2_ with organofluorine after illumination shows a weak signal at a lower wavenumber than C-F, which is assigned to the formation of Si-F bonds^57^. The XRD of CeO_2_/mSiO_2_ soaked with sodium fluoride (NaF) solution exhibits new diffraction peaks at 38.83° and 77.73°, indicating the chemical adsorption of F^−^ on the catalyst. Besides, the rationality of the chemical adsorption of mSiO_2_ and F ions was confirmed by DFT calculations, which shows a free energy change of −24.20 kJ mol^−1^ from the conversion of Si-OH to Si-F (Fig. 4C). From the perspective of reaction equilibrium, the conversion of soluble F^-^ into insoluble Si-F states is conducive to the degradation of organofluorine. Consequently, CeO_2_/mSiO_2_ not only achieves the destruction of organofluorine under sunlight irradiation but also does not release harmful F^−^, realizing the mineralization of organofluorine pollutants.

XPS measurements were conducted to compare the elemental compositions and valence states of CeO_2_/mSiO_2_ catalyst before and after the photodegradation of PFOS, OFB and FLE (Fig. 4D–F, Supplementary Figs. S33–S35). The XPS survey spectra show the characteristic peaks of Si, O and Ce elements, while the F characteristic peak (~685 eV) emerges after photodegradation (Fig. 4D). In the high-resolution XPS F 1s spectra, CeO_2_/mSiO_2_ after photodegradation of PFOS pollutants exhibit two characteristic peaks at 685.72 and 689.37 eV (Fig. 4E), which correspond to the F-C groups in organofluoride and the adsorbed F-Si groups, respectively, as confirmed by comparing reference samples before illumination and those soaked in NaF. Similarly, high-resolution XPS Si 2p spectra show an increasing peak at 104.81 eV after photodegradation or soaked in NaF solution (Fig. 4F, Supplementary Figs. S34–S36), attributed to Si-F bond formation. These results directly confirm that CeO_2_/mSiO_2_ catalyst can adsorb the free F^−^ during the photodegradation of organofluorine. Besides, high-resolution XPS Ce 3d and O 1s remain at similar binding energies after photodegradation or NaF treatment, confirming the stability of catalysts. After the third cycle, the photodegradation ability of 5%CeO_2_/mSiO_2_ photocatalyst for OFB remains above 80% (Supplementary Fig. S37). This result also indicates that the photocatalytic activity primarily depends on the CeO_2_ component. The XPS S 2p spectra of PFOS (Supplementary Fig. S34C) and N 1s of FLE (Supplementary Fig. S36F) signals completely disappear after photodegradation, confirming organofluorine pollution was effectively mineralized. Furthermore, the mineralization of organofluorides by CeO_2_/mSiO_2_ catalyst is applicable to other organofluorides, such as PFOA and fluorinated e-waste DTMDEB (Supplementary Fig. S38).

## Discussion

In summary, this work presents a potential technology for the removal of organofluorine pollutants by harnessing solar energy along a green and sustainable energy development path. CeO_2_/mSiO_2_ nanocomposites, synthesized *via* sol-gel and hydrothermal methods, possess a large specific surface area and the capability to absorb visible light. Fluorinated e-waste OFB, fluoro-antibiotic FLE, and perfluorinated pollutants PFOS were chosen as representative models featuring typical C_sp2_-F and C_sp3_-F bonds. Under visible light illumination, the optimal 5%CeO_2_/mSiO_2_ photocatalysts demonstrate efficient photodegradation performance for OFB (200 min, 99.5%), FLE (200 min, 94.4%) and PFOS (360 min, 98.3%). Notably, the degradation rate of PFOS by CeO_2_/mSiO_2_ reaches 25.9 ± 2.7% under outdoor sunlight within 5 days, significantly outperforming the parallel experiment with almost 0% degradation without catalyst. This marks the first report on the effective removal of ultra-stable PFOS under sunlight. Additionally, the influence of pH, anion and cation on the degradation performance of organofluorine pollutants was investigated through preliminary simulations of actual wastewater treatment processes. Regarding the degradation mechanism, DFT calculations illustrate the destruction pathways of C_sp2_-F and C_sp3_-F bonds by the generated •OH under illumination, which has a significant thermodynamic advantage over OH^-^ under heating conditions. Notably, CeO_2_/mSiO_2_ can adsorb F ions, enabling the removal of organic pollutants with minimal fluorine emission, This study has achieved the solar-driven mineralization of fluorine-containing organic pollutants without F emission, offering a potential green, energy-saving, and sustainable technology for the treatment of fluorine-containing organic pollutants in wastewater management.

## Methods

### Chemicals and materials

Ce(NO_3_)_3_•6H_2_O (99.5%) was purchased from Aladdin, P. R. China. Tetraethyl orthosilicate (TEOS, 99%), Hexadecyl trimethyl ammonium bromide (CTAB, 99%), octafluoro-4,4’-biphenyldiamine (OFB, 98%) were purchased from Energy Chemical Co., Ltd. HCl, NaOH, Na_2_C_2_O_4_, isopropyl alcohol (IPA), 6,8-Difluoro-1-(2-fluoroethyl)-7-(4-methylpiperazin-1-yl)-4-oxo-1,4-dihydroquinoline-3-carboxylic acid (fleroxacin, FLE, 98%), 2-[difluoro-(3,4,5-trifluorophenoxy) Methyl]-5-(4-ethylphenyl)-1,3-difluoro-benzene (BTMDEB, 97%) were purchased from Bidepharm Medical Co., Ltd. (Shanghai, China). Perfluorooctanoic acid potassium salt (PFOS, 98%) and perfluorooctanoic acid (PFOA, 98%) were purchased from the J&K Scientific. Ammonium hydroxide (25%) was purchased from Mackin Co., Ltd. (Shanghai, China). All reagents used as received without any further purification.

### Instruments

The morphology, elemental contents of the catalyst were characterized using Field Emission Scanning Electron Microscope and energy dispersive X-ray spectroscopy (SEM-EDS, Gemini 300, Germany) and transmission electron microscopy (TEM, JEM-F200, Japan). The crystalline structures of the catalysts were identified by X-ray powder diffraction (XRD, D8 ADVANCE, Germany). The surface area and pore size distribution were determined by the BET method from N_2_ adsorption-desorption isotherms at 77 K using ASAP 2020 PLUS HD88 (USA). X-ray photoelectron spectroscopy (XPS) was performed usedThermo ESCALAB 250Xi. Solid state UV–vis diffuse reflectance spectra were measured using a Lambda 950 UV/VIS Spectrometer equipped with an integrating sphere attachment, with BaSO_4_ power as background reference. Fourier transform infrared (FTIR) spectroscopy was performed over a range of 400~4000 cm^−1^ using a Nicolet iS50 spectrometer (USA). Electrochemical impedance spectroscopy (EIS) was collected using an electrochemical workstation (CHI660E, Shanghai Chenhua, China). Electron paramagnetic resonance (EPR, A300, BRUKER, USA) with 5, 5-dimethyl-1-pyrroline N-oxide (DMPO, J&K Scientific Ltd., Beijing) as radical capturer was used to detect the presence of hydroxyl radical (•OH) and superoxide radical (•O_2_^−^) under simulated sunlight irradiation. The concentrations of OFB, FLE, and DTMDEB were analyzed using a UV-8000 UV–vis analyzer (METASH, China), with detection wavelengths set at 267 nm, 270 nm, and 278 nm, respectively. TOC were measured on a SHIMADZU instrument (Total Organic Carbon Analyzer TOC-L CPH Basic System, TOC-L CPH).

### Preparation and characterization of CeO_2_/mSiO_2_ nanocomposites

The mesoporous SiO_2_ (mSiO_2_) was synthesized by sol-gel method^58^. Firstly, 1.458 g of CTAB was dissolved in 60.0 mL deionized water, and 4.4 mL TEOS and 0.56 mL ammonia were added to trigger the reaction. Thus, the molar ratio of CTAB, TEOS, deionized water and ammonia was controlled as 0.01: 0.002: 1.6: 0.015. The mixed solution was stirred at room temperature for 5 h. As the reaction proceeded, the white turbid solution gradually transforms into a clear and transparent solution, and finally forms a viscous milky white sol. After filtration, the sol was washed with deionized water three times, dried at 80 °C for 12 h, and then ground into a powder sample using an agate grinding machine. Finally, the powder samples were calcinated in the muffle furnace at 550 °C for 6 h to obtain mSiO_2_.

The CeO_2_/mSiO_2_ with different ratios were synthesized using hydrothermal methods. Initially, varying amounts of Ce(NO_3_)_3_•6H_2_O were ultrasonically dispersed in 10 mL methanol, and then 0.95 g mSiO_2_ powders were added. The solvent was removed by vacuum rotary evaporation to ensure a complete mix Ce(NO_3_)_3_·6H_2_O and mSiO_2_. Subsequently, the mixed raw materials were transferred into the ceramic crucible and calcinated in the muffle furnace at 250 °C for 3 h to obtain CeO_2_/mSiO_2_ composites. A series of mSiO_2_ with varying CeO_2_ loadings were synthesized by adjusting the mass ratio of Ce(NO_3_)_3_•6H_2_O to mSiO_2_, which were named as 5%CeO_2_/mSiO_2_, 10%CeO_2_/mSiO_2_, 20%CeO_2_/mSiO_2_, 50%CeO_2_/mSiO_2_, and 80%CeO_2_/mSiO_2_, respectively.

### Photodegradation experiments

Photodegradation experiments were conducted under the irradiations of a pure 410-nm LED lamp, and a 300 W xenon lamp as well as outdoor sunlight, respectively. Saturated aqueous solution of OFB (11.4 μmol L^−1^ at R.T.)^46,48^, 50.0 μmol L^−1^ FLE and 10.0 μmol L^−1^ PFOS were used as organofluorine pollutant solutions to evaluate their photodegradation performance. The light interval was set, and after each interval, 3 mL of the reaction solution was centrifuged and filtered to test the change in organofluorine concentrations. It is worth noting that the concentration of OFB and FLE were monitored by UV-Vis absorption spectroscopy. Whereas the concentrations of PFOS was detected by HPLC-MS. The degradation rate was calculated using the formula: (C_0_-C_t_)/C_0_, where C_t_ is the concentration of degraded organo-fluorine pollutants, and C_0_ is the initial concentration of organofluorine pollutants. The optimization of the pH of the reaction solution was regulated by 0.1 M NaOH and HCl.

### Intermediates and products analysis

The intermediates of OFB, PFOS and FLE were analyzed using a Thermo Ultimate 3000 Infinity HPLC System equipped with a Thermo TSQ ENDURA LC/MS System, which utilized electrospray ionization (ESI) in both positive and negative ion modes, along with Q1 Full Scan for the target analyzed. The chromatographic column used was an AC Agilent ZORBAX SB-C18 (2.1 mm × 100 mm, 1.8 μm). Prior to detection, all samples were filtered through 0.22 μm needle-like filters. The intermediate products were identified by liquid chromatography-mass spectrometry (LC-MS) analysis with the following solvent systems: for OFB, a mixture of water (20%) and acetonitrile (80%) at a flow rate of 0.3 mL min^−1^; for FLE, a mixture of methanol (25%) and water (75%) at a flow rate of 0.8 mL min^−1^; and for PFOS, 2 mM ammonium acetate in acetonitrile (100%) at a flow rate of 0.5 mL min^−1^. Mass spectrometry (MS) was performed in an electrospray ionization (ESI) mode, with the scanning mass ranged set from 50 to 500 m/z.

### DFT calculations

DFT calculations for compounds and intermediates were performed using the Gaussian 09 program. For geometries optimizations and singlet point energy calculations, the M062X/6-31 g(d) and M062X/6-311 g(2 d) levels of theory were employed. Intrinsic reaction coordinate (IRC) calculations were executed to identify transition states and ensure their connection to the corresponding intermediates. The bond dissociation energies (BDE) were computed at an identical theoretical level^59,60^.

## Supplementary information

Supplementary materials

## Data Availability

The data supporting the findings of this work are available within the article and its Supplementary Information files. All the data reported in this work are available from the authors. Source data are provided with this paper.
