# Supplementary material for: Solar-driven defluorination via hydroxyl radical spillover for complete mineralization of organofluorine pollutants without fluoride byproducts
Source: Commun Chem. 2025 Aug 16;8:249. doi: 10.1038/s42004-025-01655-3 (PMC12357893; doi:10.1038/s42004-025-01655-3)
Supplement: Supplementary file 1 — Supplementary materials [file 42004_2025_1655_MOESM1_ESM.pdf]

Supplementary Materials for

Solar-driven defluorination *via* hydroxyl radical spillover for complete mineralization of organofluorine pollutants without fluoride byproducts

Lei Zheng,[1,2,†] Jing-Lan Zhang,[1,†] Zhixin Zheng,[2] Chujie Huang,[2] Yi-Lin Xie,[1] Xu-Bing Li,[3] Fentahun Wondu Dagnaw,[1] Tieyu Wang,[2,] Qing-Xiao Tong,[1,*] Jing-Xin Jian[1,2,*]*

Corresponding authors: jxjian@stu.edu.cn; wangt@stu.edu.cn; qxtong@stu.edu.cn

The PDF file includes:

Supplementary Text
Figs. S1 to S38
Tables S1 to S4
References
Data S1

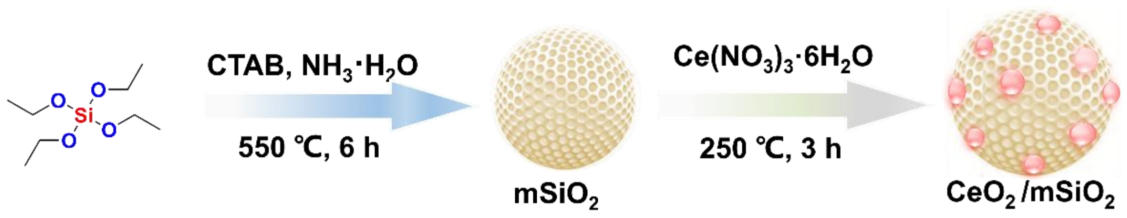

Fig. S1. The preparation of $mSiO_2$ and CeO_2/$mSiO_2$ nanocomposites.

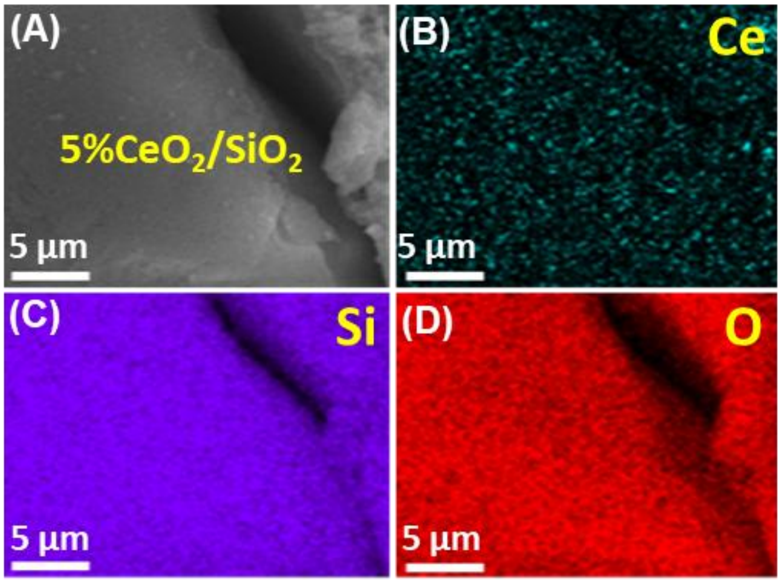

Fig. S2. SEM image of 5%CeO_2/$mSiO_2$ with EDS mapping of Ce (B), Si (C) and O (D) elements.

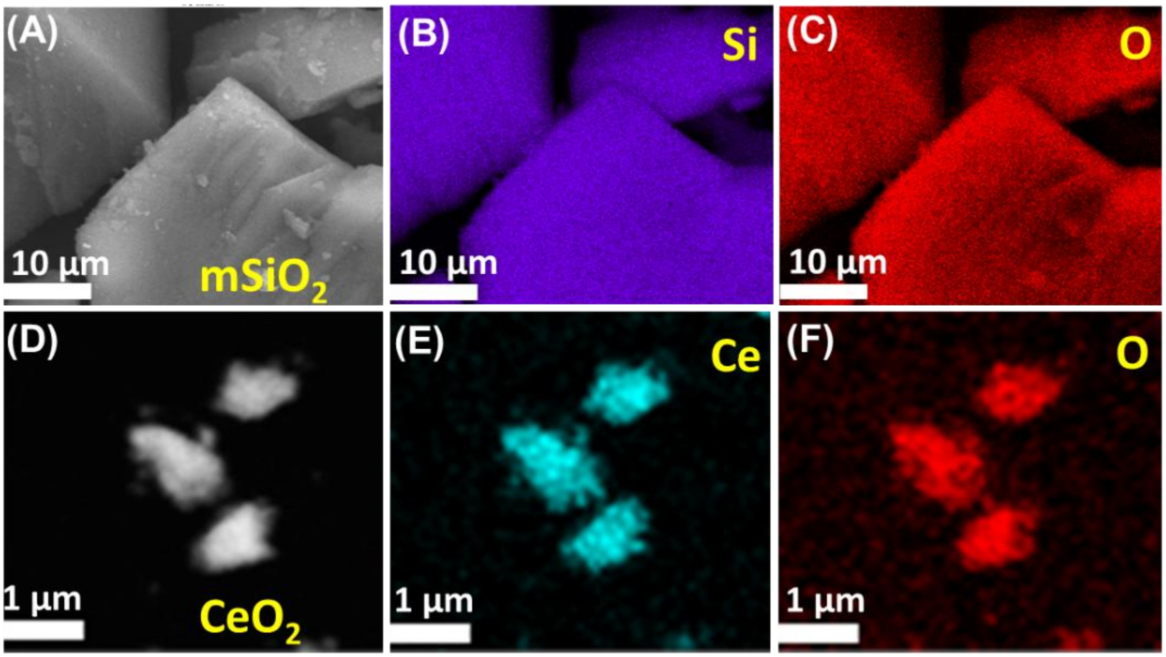

Fig. S3. SEM image of $mSiO_2$ nanoparticles (A) with EDS-mapping of Si (B), O (C) elements. SEM image of CeO_2 nanoparticles (D) with EDS-mapping of Ce (E), O (F) elements.

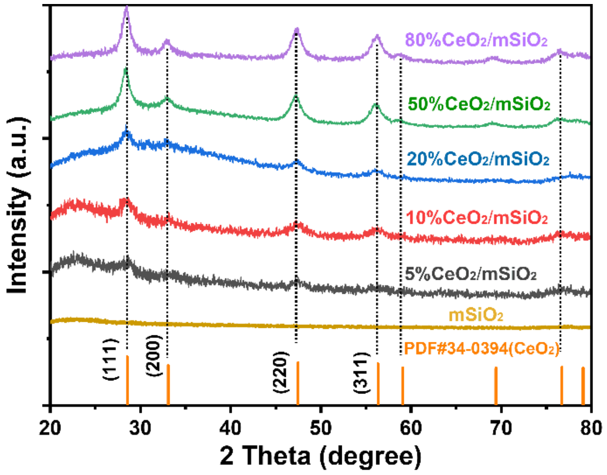

Fig. S4. XRD patterns of $CeO_2/mSiO_2$ with different loading percentages of CeO_2.

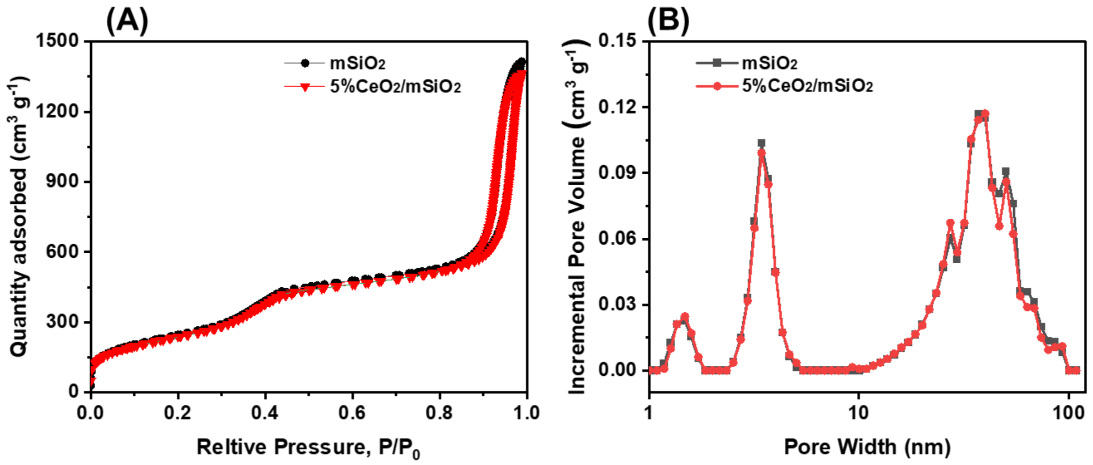

Figure S5. N_2 adsorption/desorption isotherms of $mSiO_2$ and $5\%CeO_2/mSiO_2$ (A) and the pore size distribution (B).

Table S1. The physical properties of prepared $mSiO_2$ and CeO_2/SiO_2 catalyst.

Sample	Surface area (m^2 g^{-1})	pore size (nm)	References
BMS ($mSiO_2$)	896	5	[1]
SiO_2 aerogel	881	6.7	[2]
l-$mSiO_2$ nanospheres	525	10	[3]
CeO_2/SiO_2-200 aerogel	764	7.7	[2]
CeO_2/SiO_2	230		[4]
$mSiO_2$	913.7	1.5, 3.8, 30-60	This work
$5\%CeO_2/mSiO_2$	884.7	1.5, 3.8, 30-60	This work

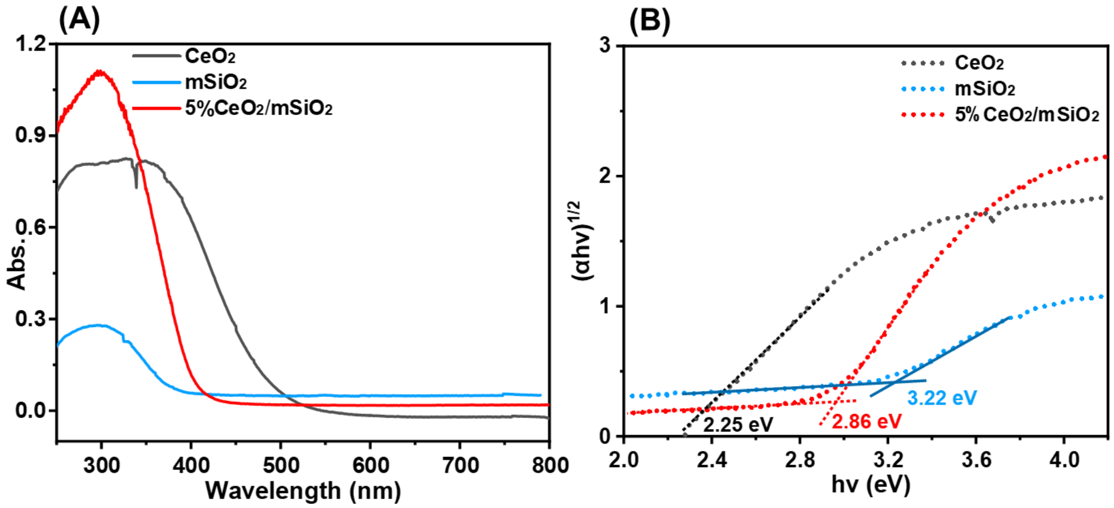

Fig. S6. Absorption spectra (A) and Tauc plot (B) of $mSiO_2$, CeO_2 and 5%CeO_2/$mSiO_2$.

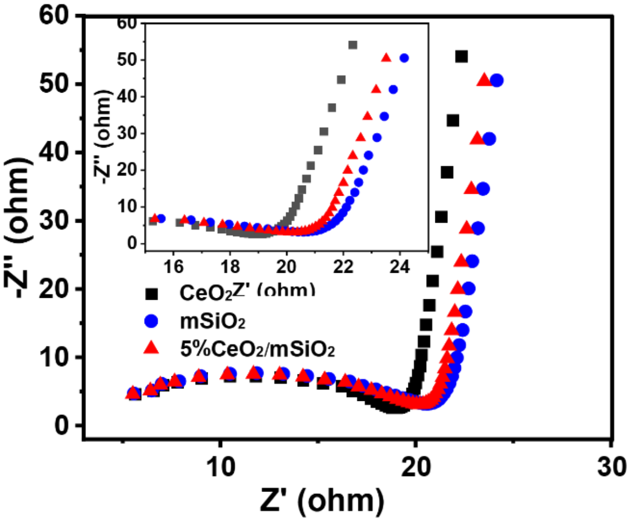

Fig. S7. Nyquist plots of CeO_2, $mSiO_2$, and 5%CeO_2/$mSiO_2$.

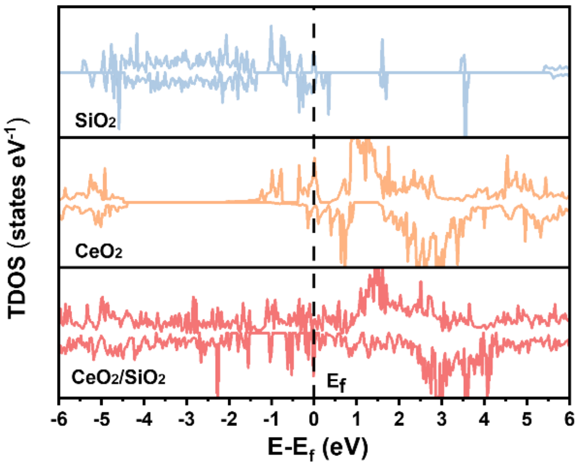

Fig. S8. DFT calculated total density of states (TDOS) plots of CeO_2, $mSiO_2$, and $CeO_2/mSiO_2$.

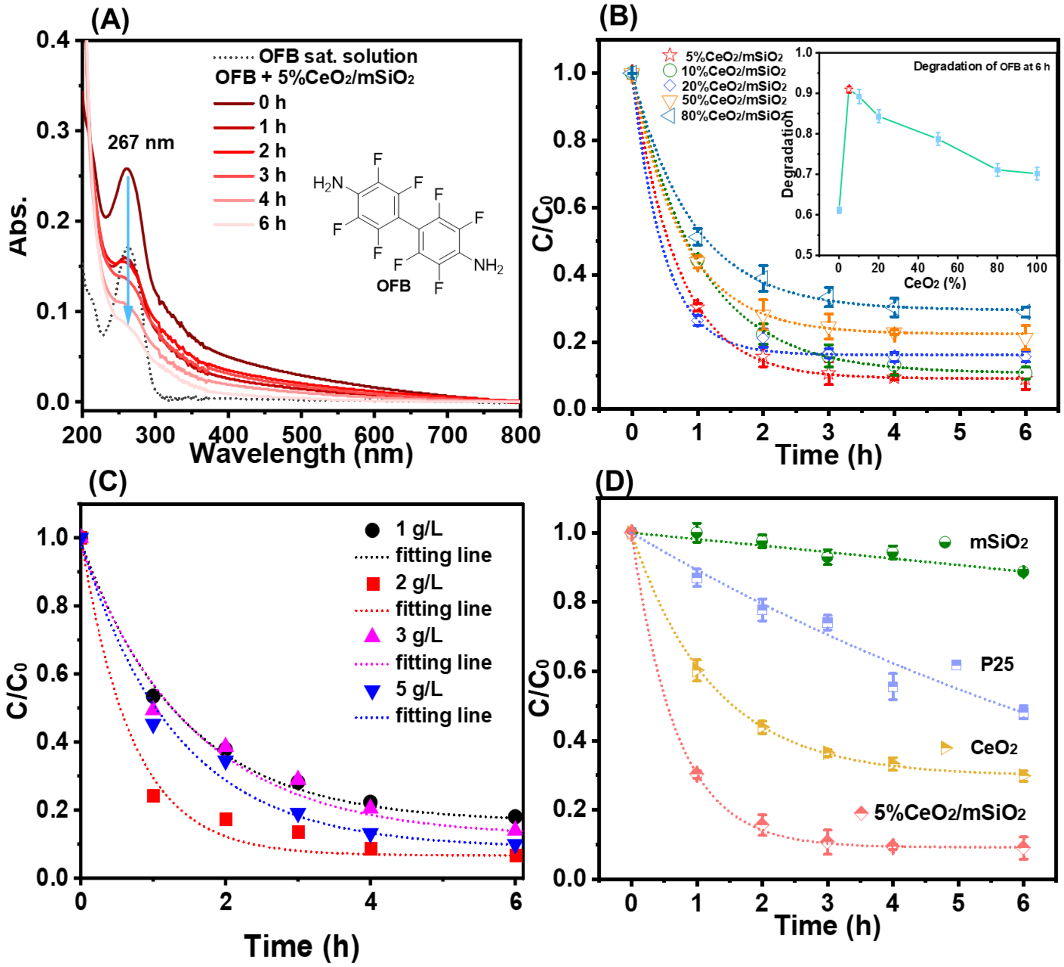

Fig. S9. (A) UV-vis absorption spectra of OFB solutions. Photodegradation of OFB by $CeO_2/mSiO_2$ catalysts with different CeO_2 loadings (B) and with different amounts of

5%CeO_2/mSiO_2 catalyst (C). (D) Photodegradation of OFB with commercial P25 (TiO_2), mSiO_2, CeO_2, and 5%CeO_2/mSiO_2 catalysts.

Supplementary Notes 1: Firstly, the comparative experiments of photodegradation of organic fluorine pollutants were carried out in dark, no 5%CeO_2/mSiO_2 catalyst or simulated sunlight (Xe lamp with AM 1.5 G filter). As shown in Fig. S9A, the characteristic absorption peak of OFB at 267 nm in aqueous solution significantly decreased under the illumination of visible light (410 nm LEDs, ~50 mW/cm^2), which was attributed to its photodegradation process. Moreover, the photodegradation of OFB by CeO_2/mSiO_2 catalysts with different CeO_2 loadings was investigated under the same conditions. As illustrated in Fig. S9B, CeO_2/mSiO_2 with low CeO_2 loading of 5% presented the best photodegradation activity. Compared with the reported noble metal oxide catalysts of In_2O_3 and Ga_2O_3,[5-7] low loading of CeO_2 has higher atomic economy and decreases the toxic damage of heavy metal ions to the ecological environment. The optimal amount of CeO_2/mSiO_2 photocatalyst was 2.0 g/L (Fig. S9C). After continuous illumination for 6 h, the degradation rate of OFB by 5%CeO_2/mSiO_2 reached 91.1 ± 3.2 % (Fig. S9D). In outdoor photodegradation of OFB, FLE, and PFOS, 20 mL of organofluorine pollutant was poured into a 100 mL beaker, and the amount of CeO_2/mSiO_2 photocatalyst was set to 2.0 g/L. Four parallel experiments were performed for each sample.

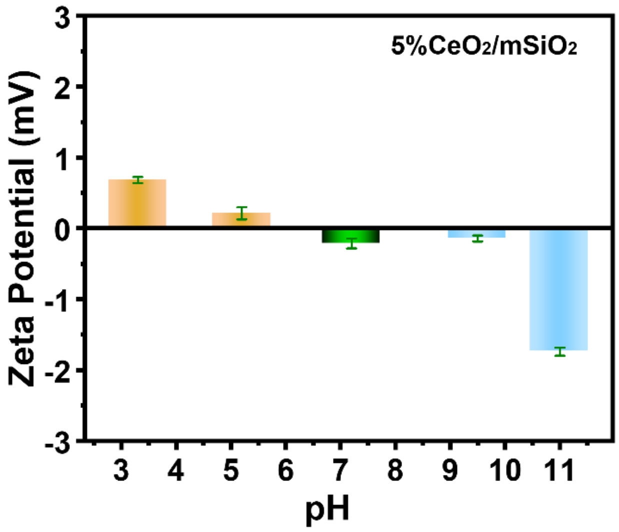

Fig. S10. Zeta potential of 5%CeO_2/mSiO_2 nanocomposites at different pH conditions.

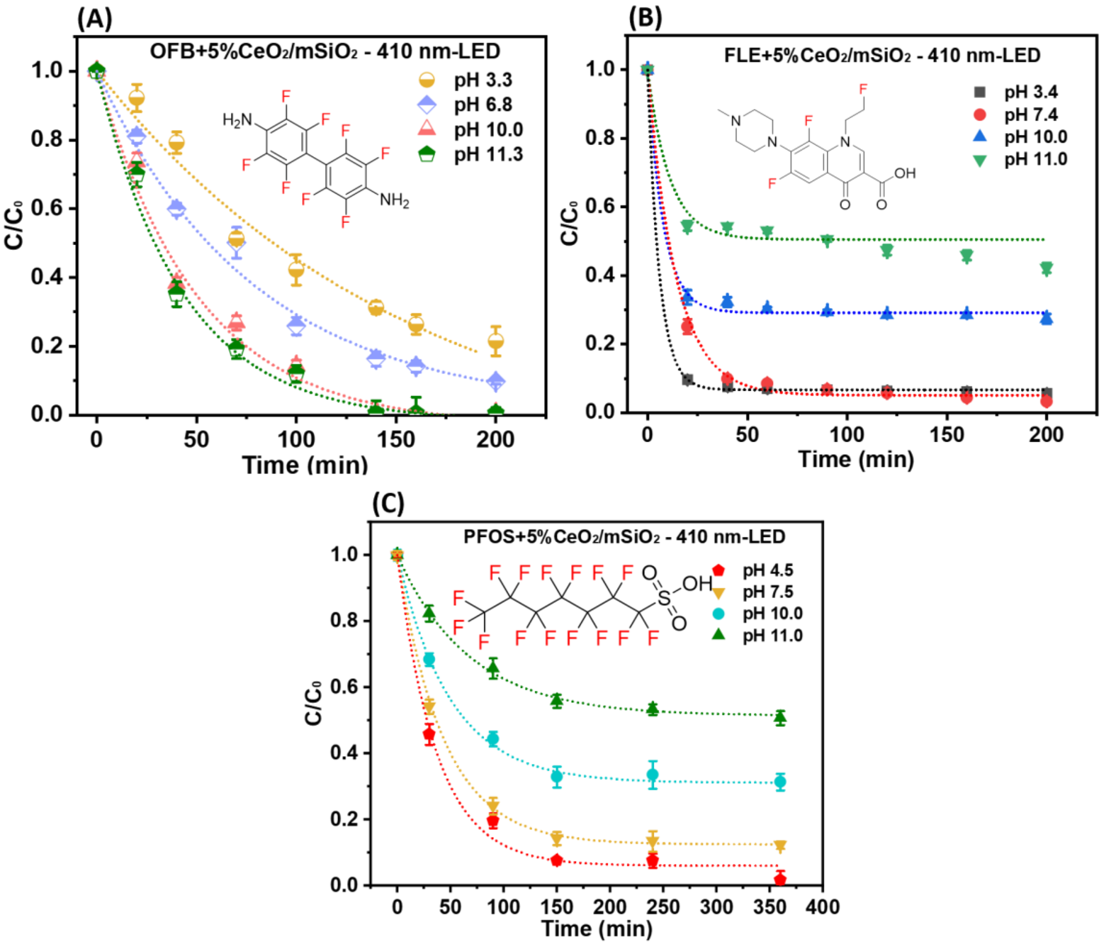

Fig. S11. Photodegradation of OFB (A), FLE (B), and PFOS (C) at different pH values by 5%CeO_2/mSiO_2.catalyst under the irradiation of 410-nm LED.

Supplementary Notes 2: The degradation rate of OFB under alkaline conditions (pH 11.3) reached 99.5±1.7% after 200 min of 410 nm-LED illumination, outstripping the degradation rates of 78.5±4.2% under acidic conditions (pH 3.3) and 90.3±0.7% in neutral environments (pH 6.8). While, the degradation of FLE under acidic conditions reached 94.4±0.1% after 200 min irradiation, and the degradation of PFOS reached 98.3±2.8% after 360 min irradiation, which was superior to that in alkaline conditions.

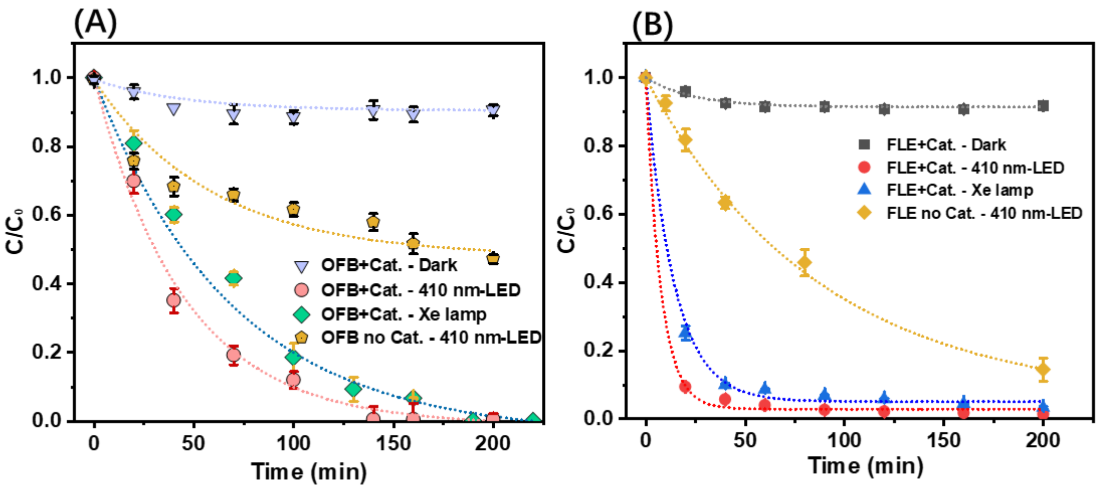

Fig. S12. Photodegradation of OFB (A) and FLE (B) by 5%CeO_2/$mSiO_2$ catalyst (2.0 g/L) in the dark and under the irradiation of 410-nm LED and Xe lamp.

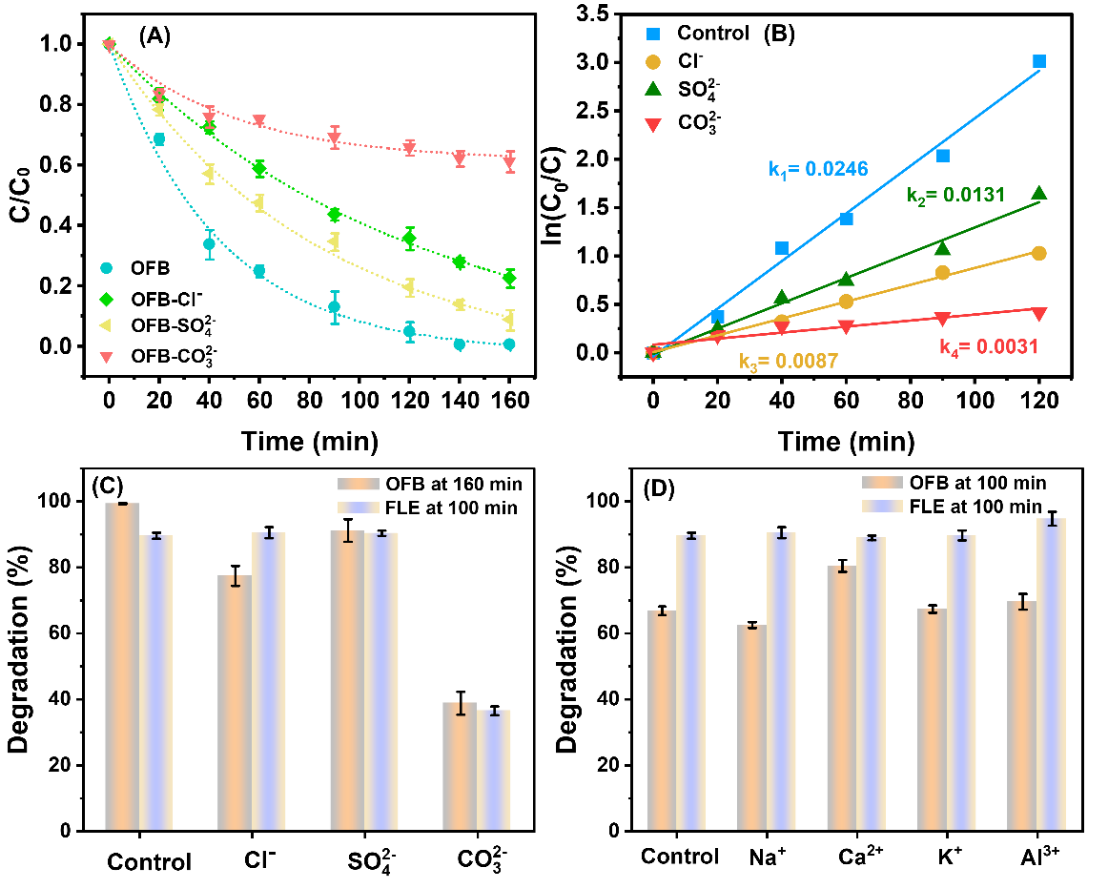

Fig. S13. Photodegradation of OFB with different anions (A) and the corresponding kinetics fitting curve (B). Photodegradation of OFB and FLE with different anions (C) and cations (D).

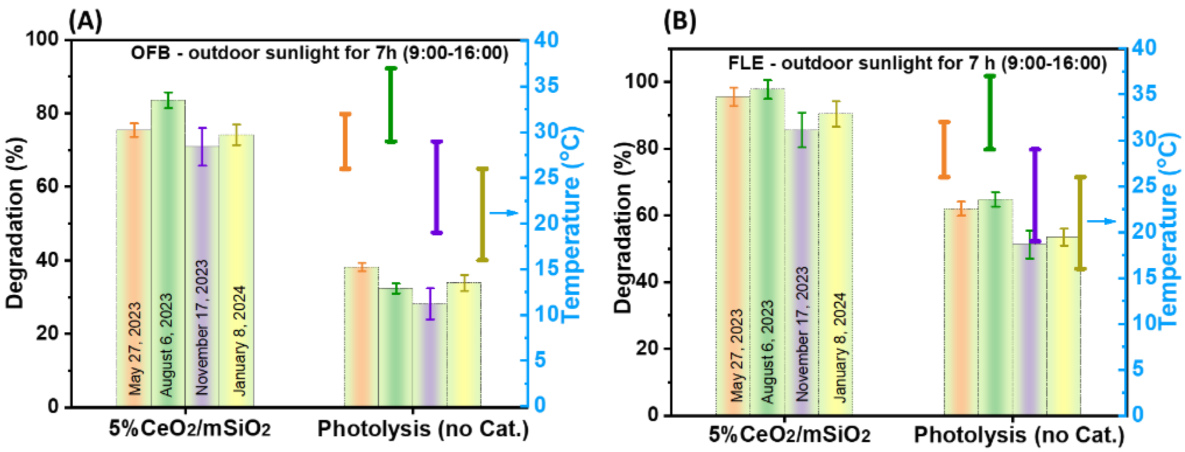

Fig. S14. The removal of OFB (A) and FLE (B) in the absence and presence of 5%CeO_2/mSiO_2 catalyst under outdoor sunlight, with the average daily temperatures.

Table S2. Comparative analysis of solar-driven mineralization method with the reported advanced oxidative processes.

Methods	Time (min)	Deg. (%)	Contaminant (C%)	Conditions	Refs.
Electrocatalytic redox (Co-CN_2-Fe_2O_3)	120	96.1	PFOA (10 mg/L)	-0.06 V in an H-cell equipped with a gas diffusion cathode (pH 2)	8
Plasma reactor	120	>99	PFCAs (100 mg/L)	Energy requirement (EE/O):380 to 830 kWh/m^3	9
Dual-frequency ultrasonic activated persulfate	360	47	PFOS (5.79 μM)	frequency = 20 kHz @ 550 W + 43 kHz @ 250 W (pH 3.69)	10
Ultrasonication	180	46	PFAS (1 mg/L)	frequency = 130 kHz	9
Chemicals redox (KOH, $K_2S_2O_8$, Na_2SO_3)	480	94-103	PFSAs (n=4,6,8,0.025 mM)	120 °C/UV (pH < 2 or >12)	11
Solar-driven defluorination CeO_2/mSiO_2	180	96.71	PFOS (10 μM)	300 W xenon lamp/400 nm LED (pH)	This work

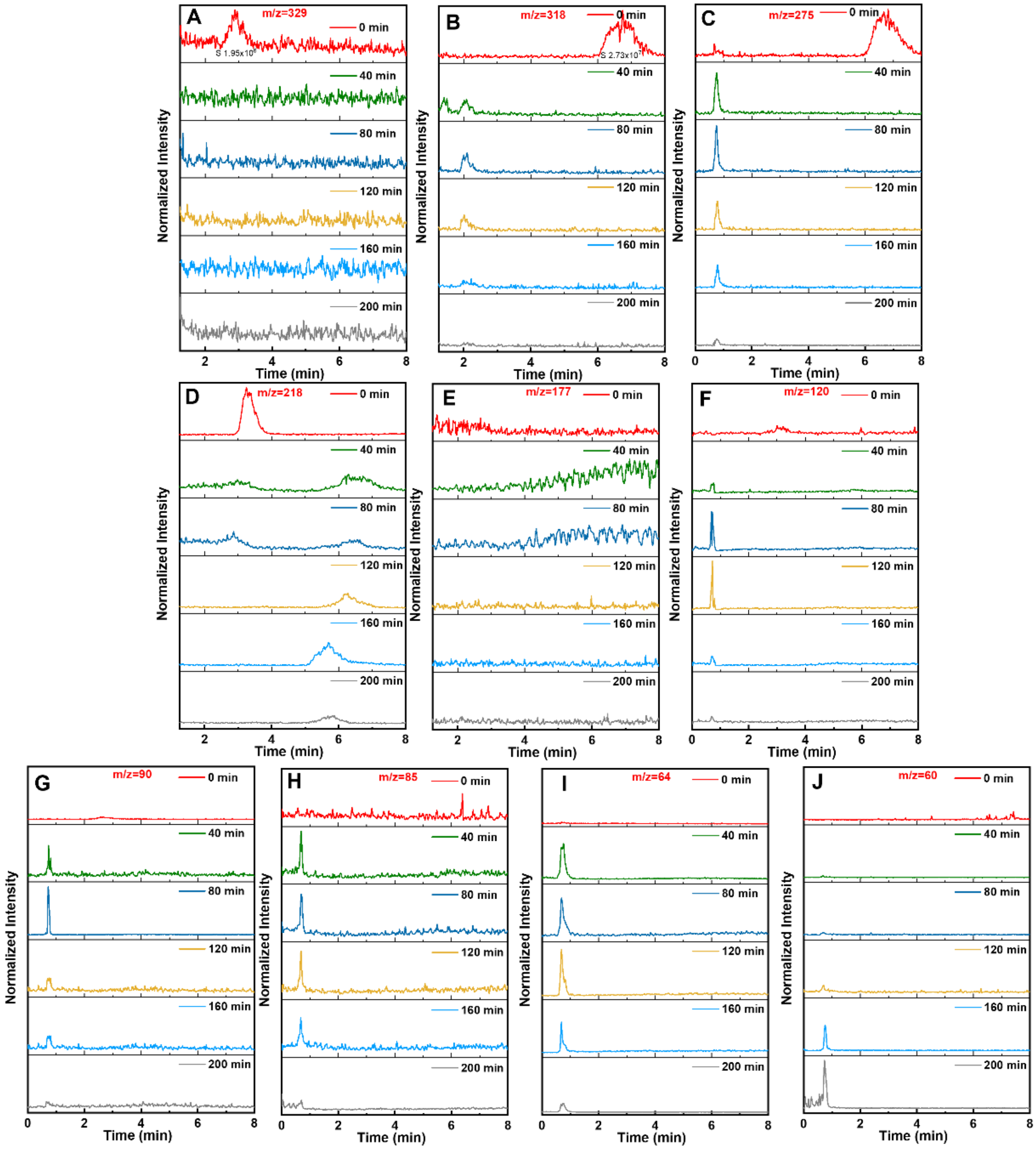

Fig. S15 (A-J) LC-MS of OFB with 5%CeO_2/$mSiO_2$ catalyst after 0, 40, 80, 120, 160 and 200 min of illumination, with m/z 329, 318, 275, 218, 177, 120, 90, 85, 64 and 60, respectively.

Supplementary Notes 3: Before illumination, OFB solution showed two sets of signal peaks in 2.5-3.5 min with characteristic m/z peaks of 329, 218, 177, 120 and 90, and peaks in 6.0-8.0 min with characteristic m/z of 318, 275 and 85. The first group of signals is derived from protonated OFB with shortened retention time in liquid chromatography due to its increased

polarity after protonation, while the latter group of peaks comes from the ion fragment signal of OFB component. Compared to the initial intensities of m/z 329 (1.95 × 10^6) and m/z 318 (2.73 × 10^7) signals, OFB did not undergo protonation under the LC-MS test conditions but reacted with hydroxyl groups to form intermediate **a2** (m/z 318). It is worth noting that OFB has two groups of fluorine atoms. The fluorine atom at the ortho positions of the amino group is highly active and prone to substitution, while the fluorine atom at the meta positions of the amino group is inert and has a large steric hindrance. The signal changes of each ion fragment were tracked during the 200 min of illumination to clarify the photodegradation paths of OFB.

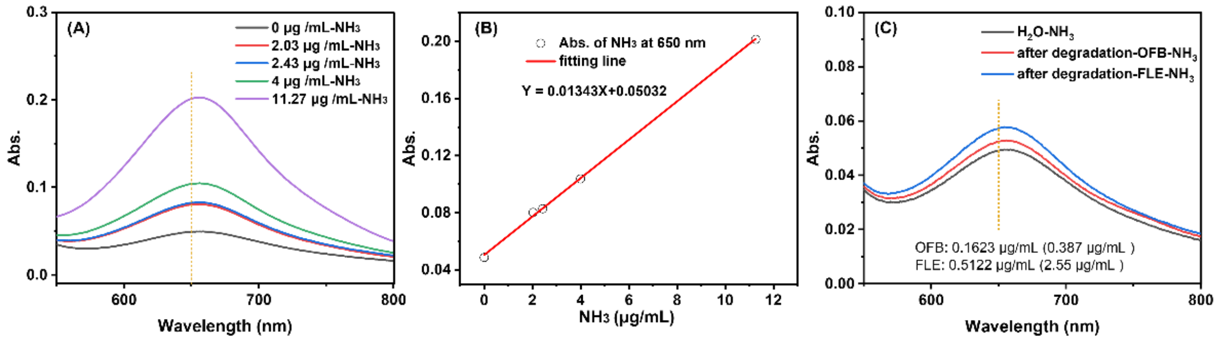

Fig. S16. The indophenol blue method was used to track the production of NH_3 in the degraded OFB and FLE.

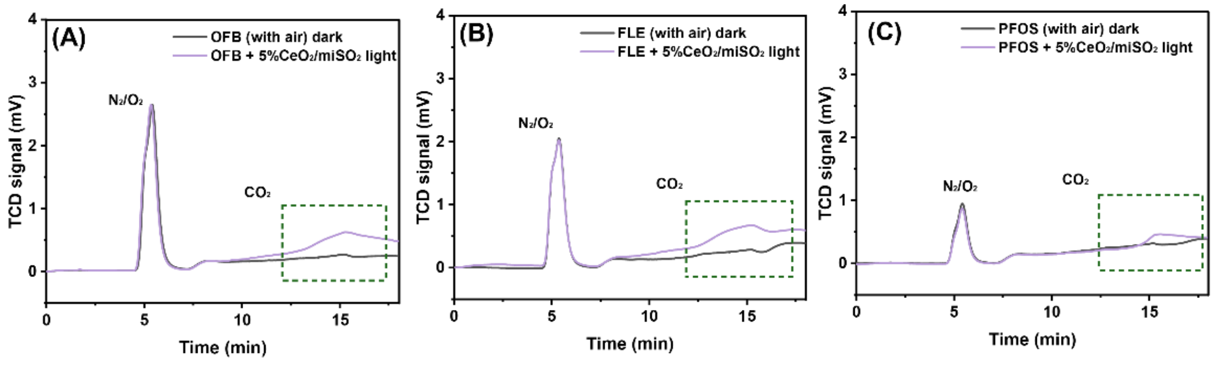

Fig. S17. Gas products of CO_2 were detected by TCD detectors after the photodegradation of OFB (A), FLE (B) and PFOS (C) by 5%CeO_2/mSiO_2 catalyst.

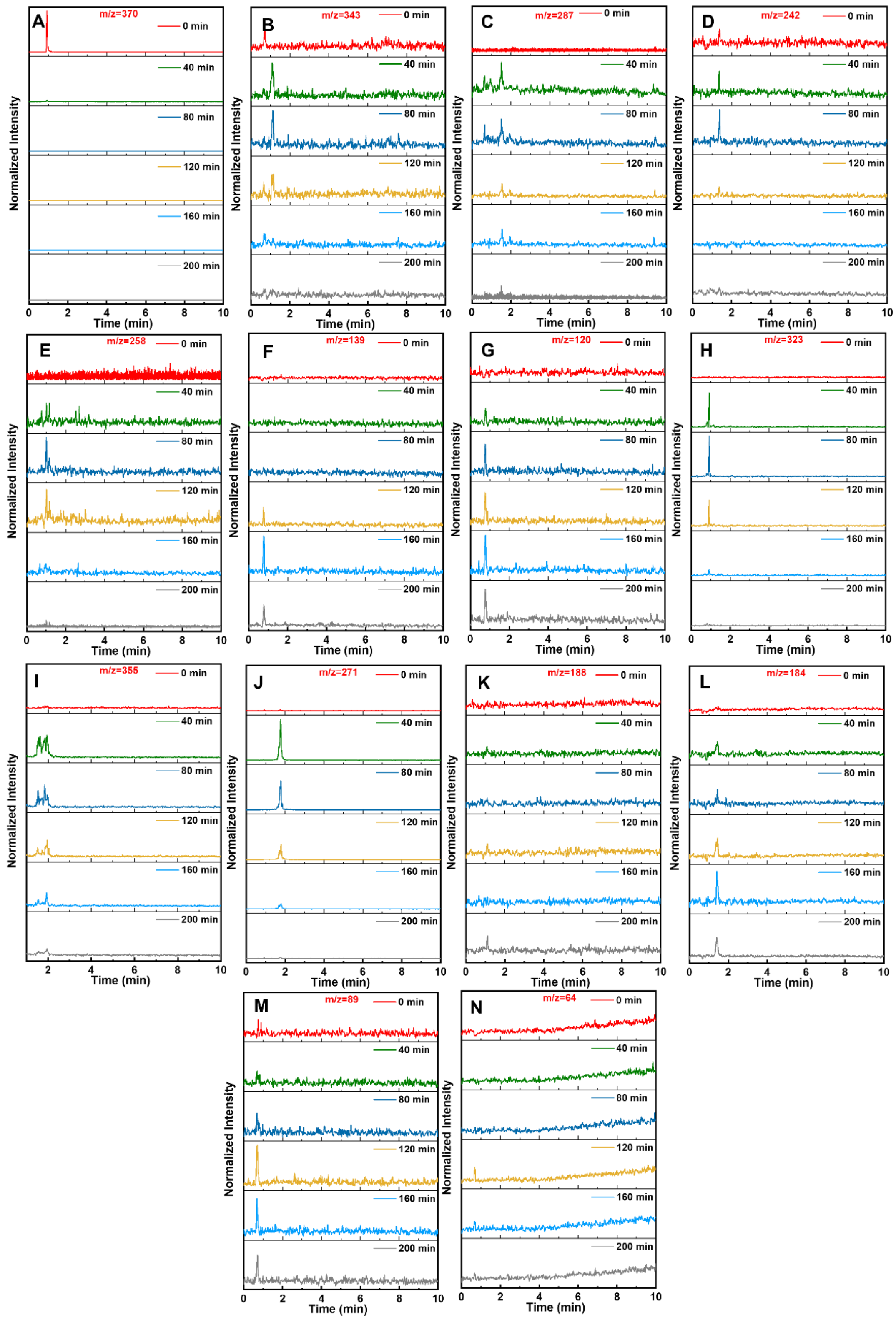

Fig. S18. LC-MS of FLE after 0, 40, 80, 120, 160 and 200 min of illuminations, with m/z 370, 243, 287, 242, 258, 139, 170, 323, 355, 271, 188, 184, 89 and 64, respectively. (A-N)

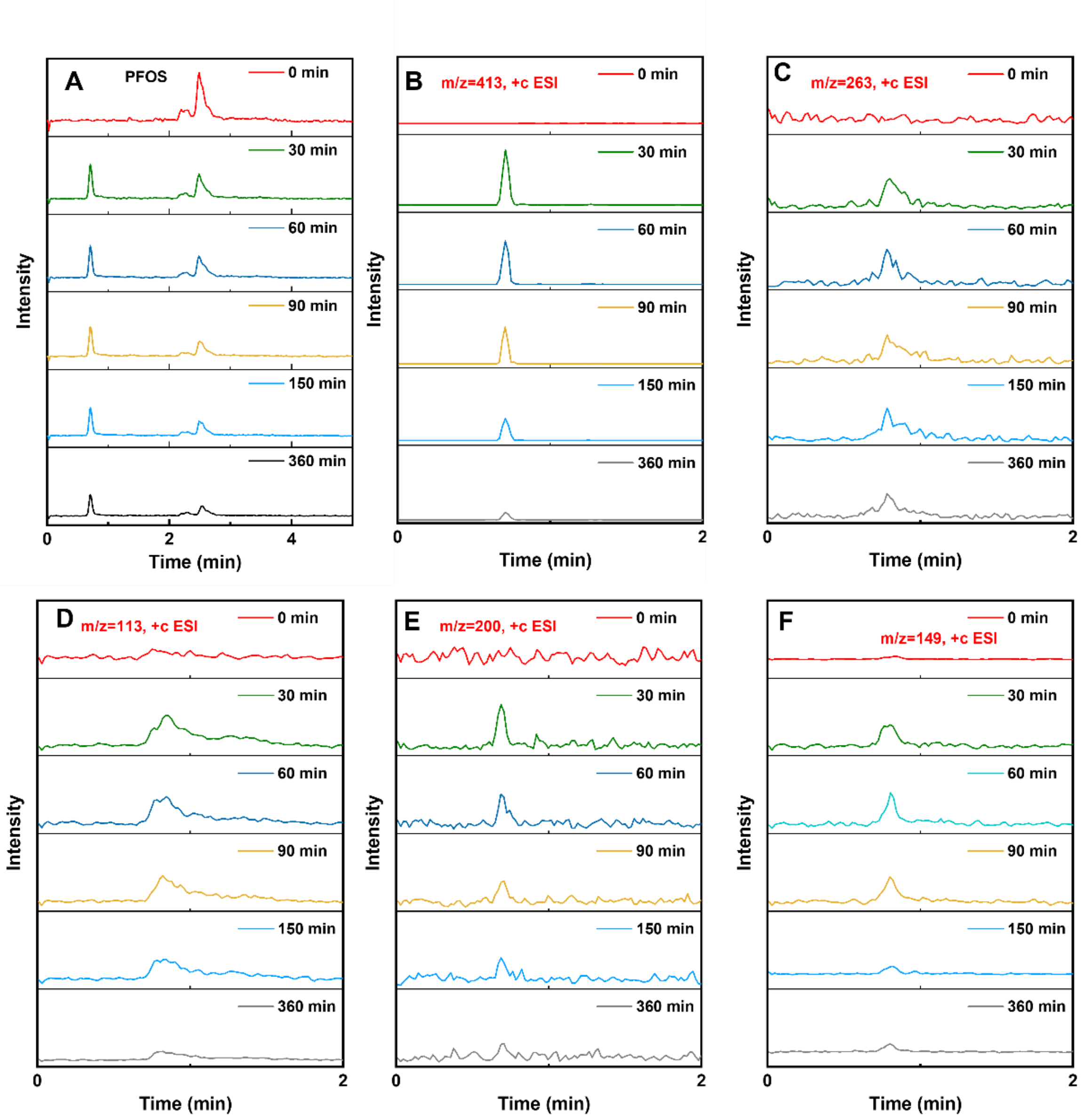

Fig. S19. (A-F) LC-MS of PFOS with 5%CeO_2/mSiO_2 catalyst after 0, 30, 60, 90, 150 and 360 min of illumination, with m/z 499, 413, 263, 113, 200 and 149, respectively.

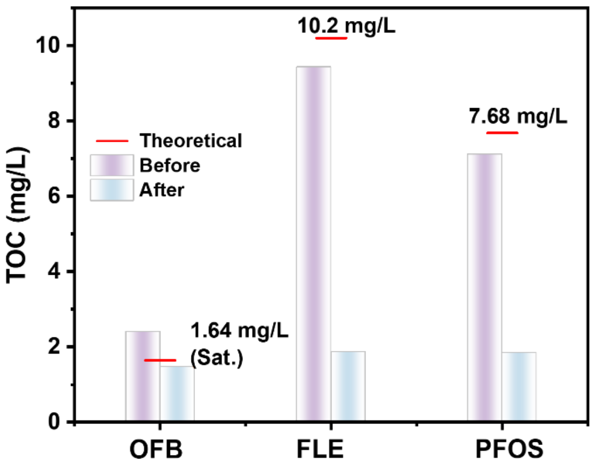

Fig. S20. TOC of OFB (11.4 μM), FLE (50.0 μM), PFOS (80.0 μM) solutions before and after photodegradation by $CeO_2/mSiO_2$ catalysts.

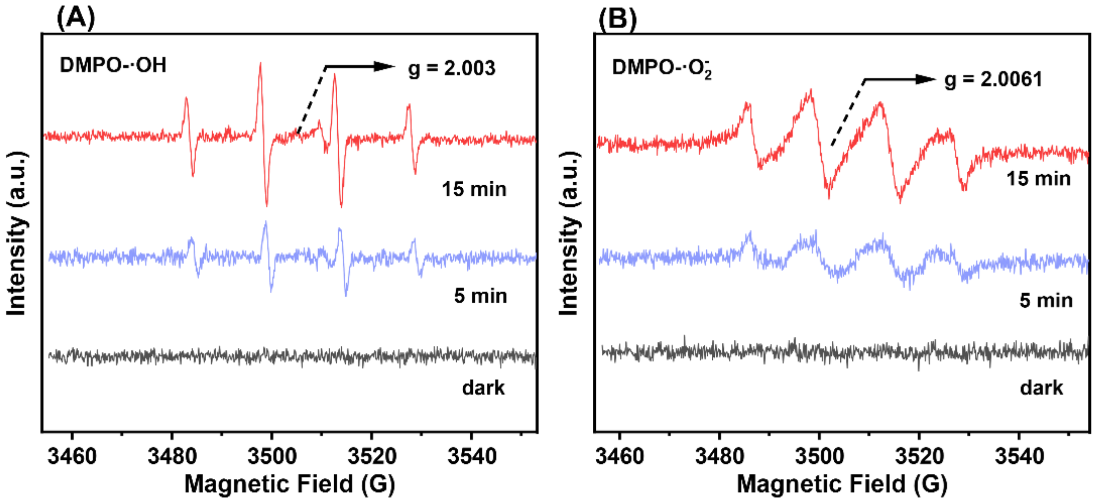

Fig. S21. EPR spectra of •OH (A) and $•O_2^-$ (B) on 5%$CeO_2/mSiO_2$ catalyst under irradiation.

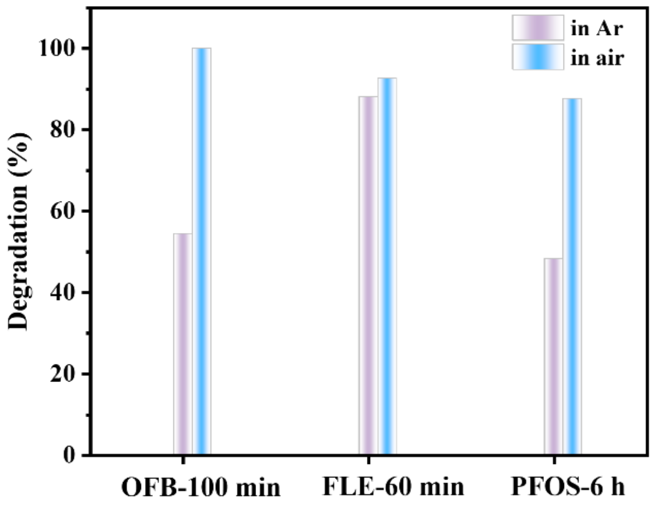

Fig. S22. Photodegradation of OFB, FLE and PFOS under the irradiation in air or Ar.

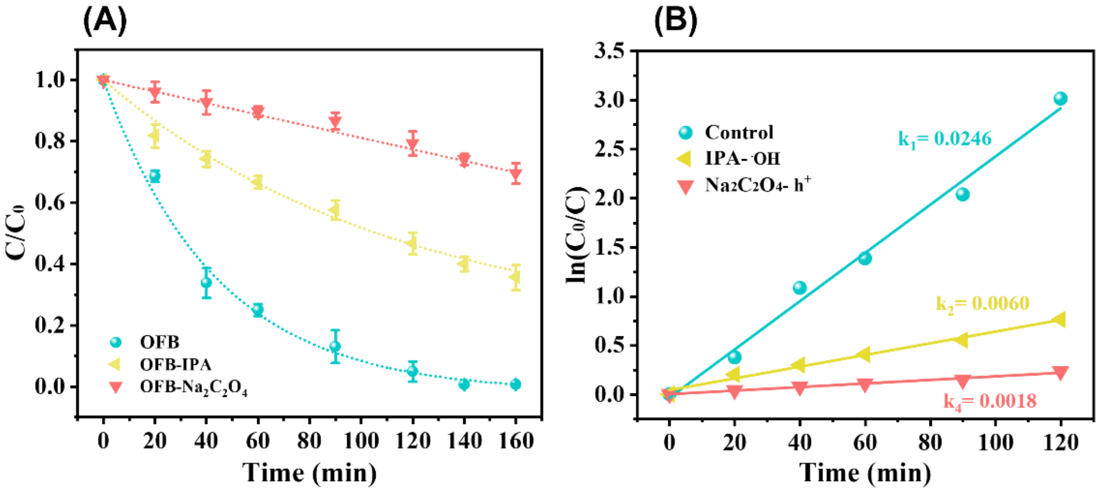

Fig. S23. Photodegradation of OFB with adding isopropanol (IPA) and sodium oxalate ($Na_2C_2O_4$) to quench •OH and h^+ (A) and the corresponding degradation kinetics fitting curve (B).

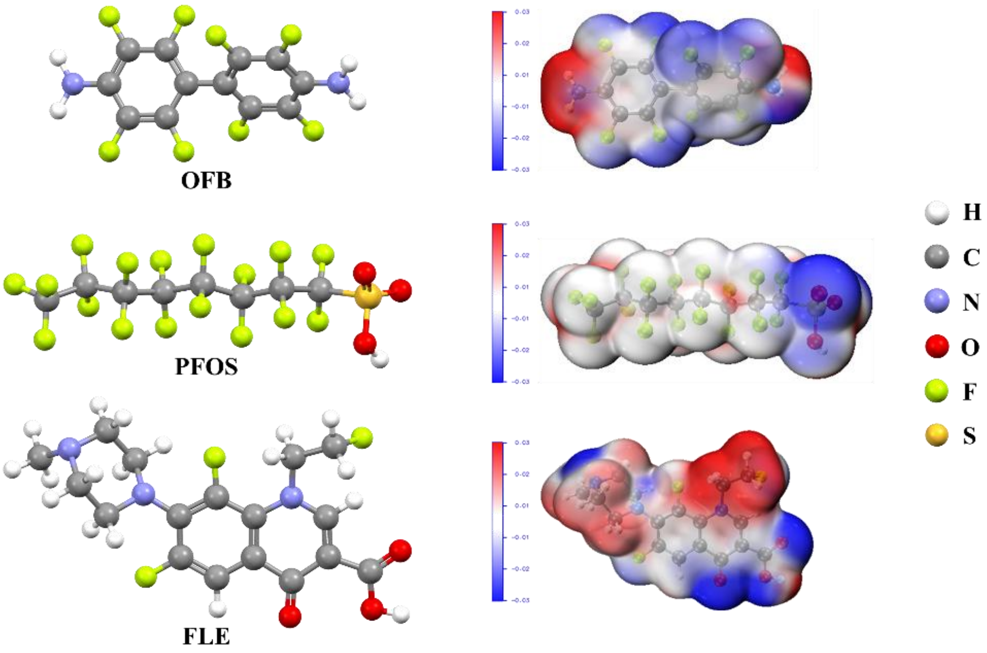

Fig. S24. Chemical structures and electrostatic potential (ESP) distributions of OFB, FLE, and PFOS.

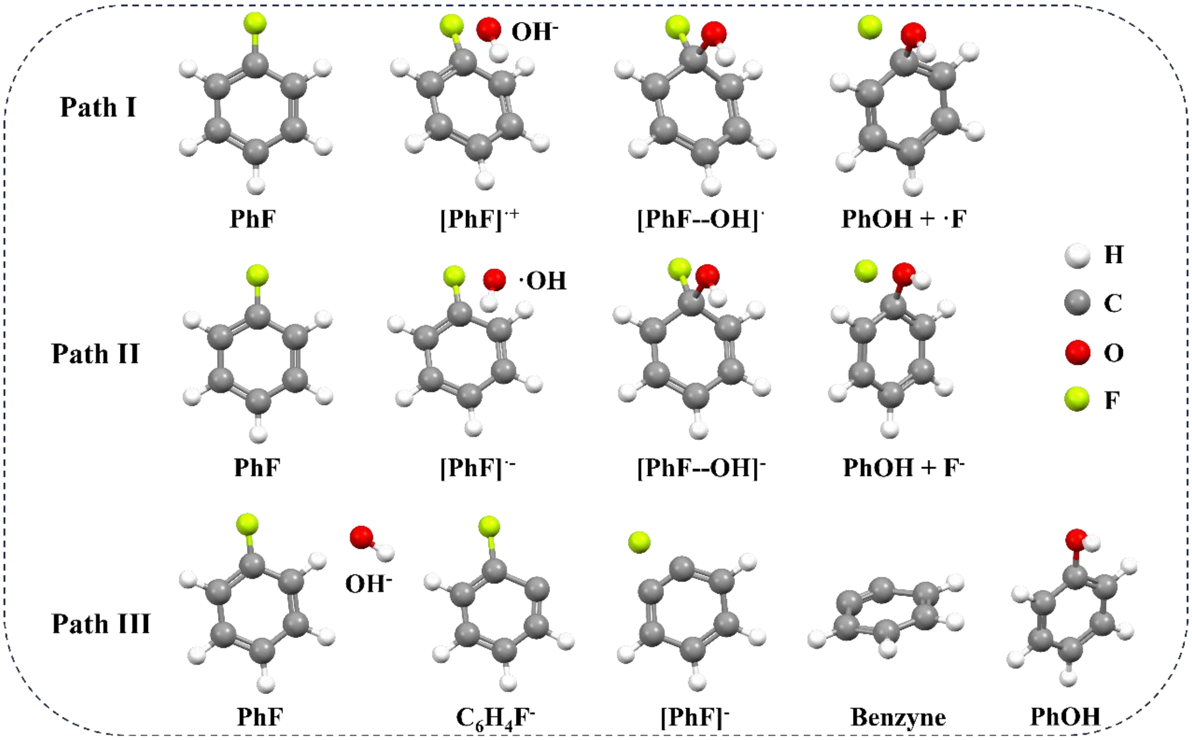

Fig. S25. DFT calculation of the defluorination of PhF to produce PhOH through the nucleophilic elimination (Path I), oxidation (Path II), and reduction pathways (Path III).

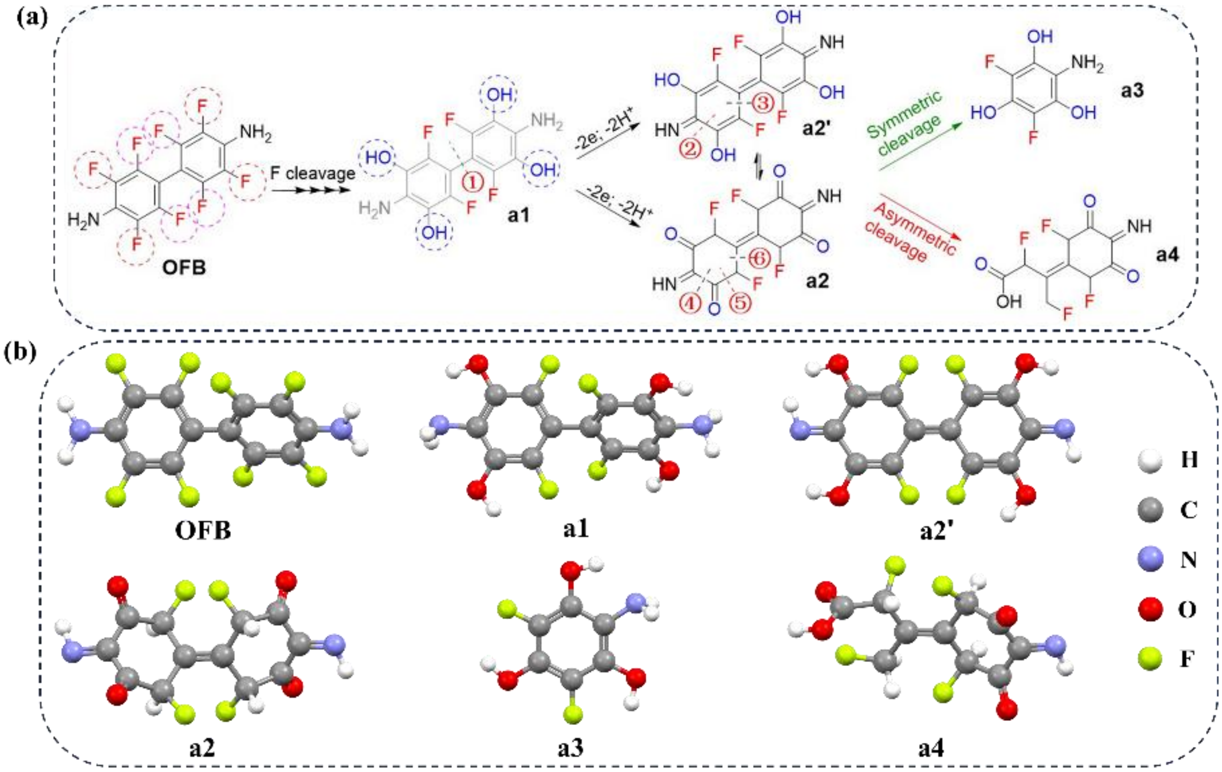

Fig. S26. DFT calculations of the C-C bond cleavage in OFB intermediates.

Table S3. The C-C bond energies of OFB.

OFB	①	②	③	④	⑤	⑥
C-C bond energy （kcal/mol）	130.95	109.76	103.50	46.86	39.98	61.08

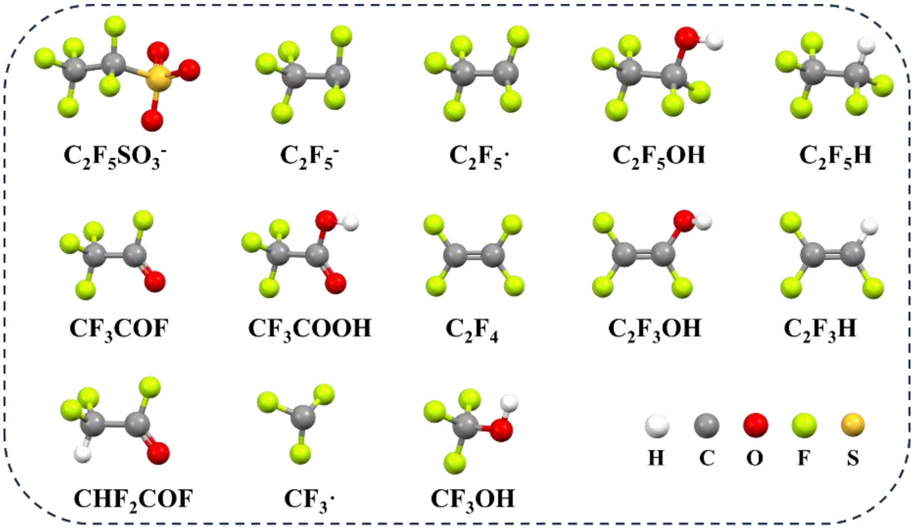

Fig. S27. DFT calculations of the degradation of PFeSA by •OH and OH^-.

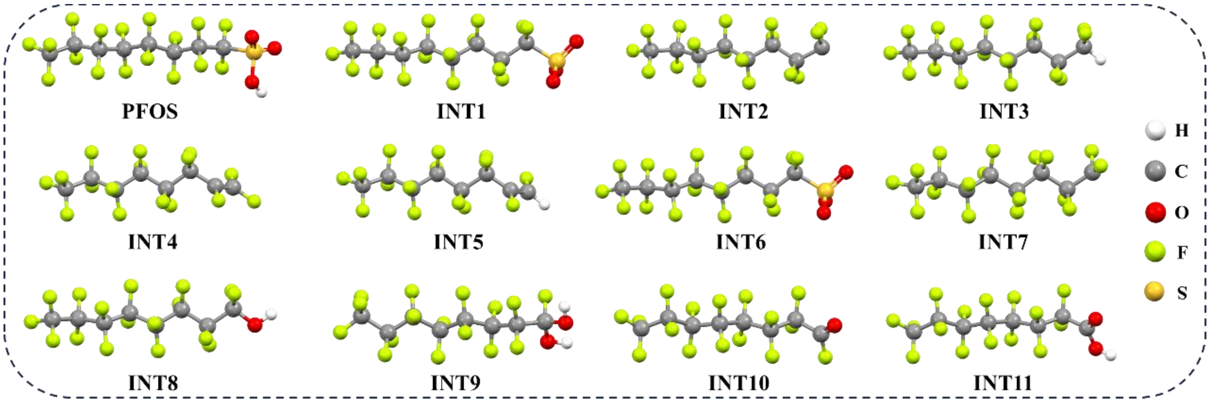

Fig. S28. DFT calculations of the degradation of PFOS by OH^- and •OH.

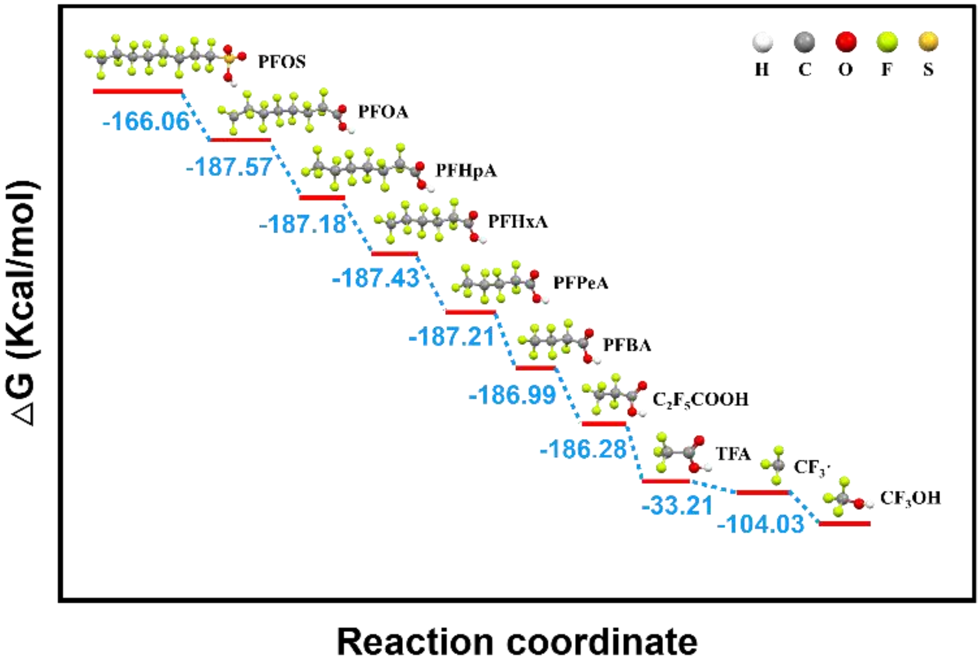

Fig. S29. The free energy changes of the stepwise chain-shortening reaction of PFOS.

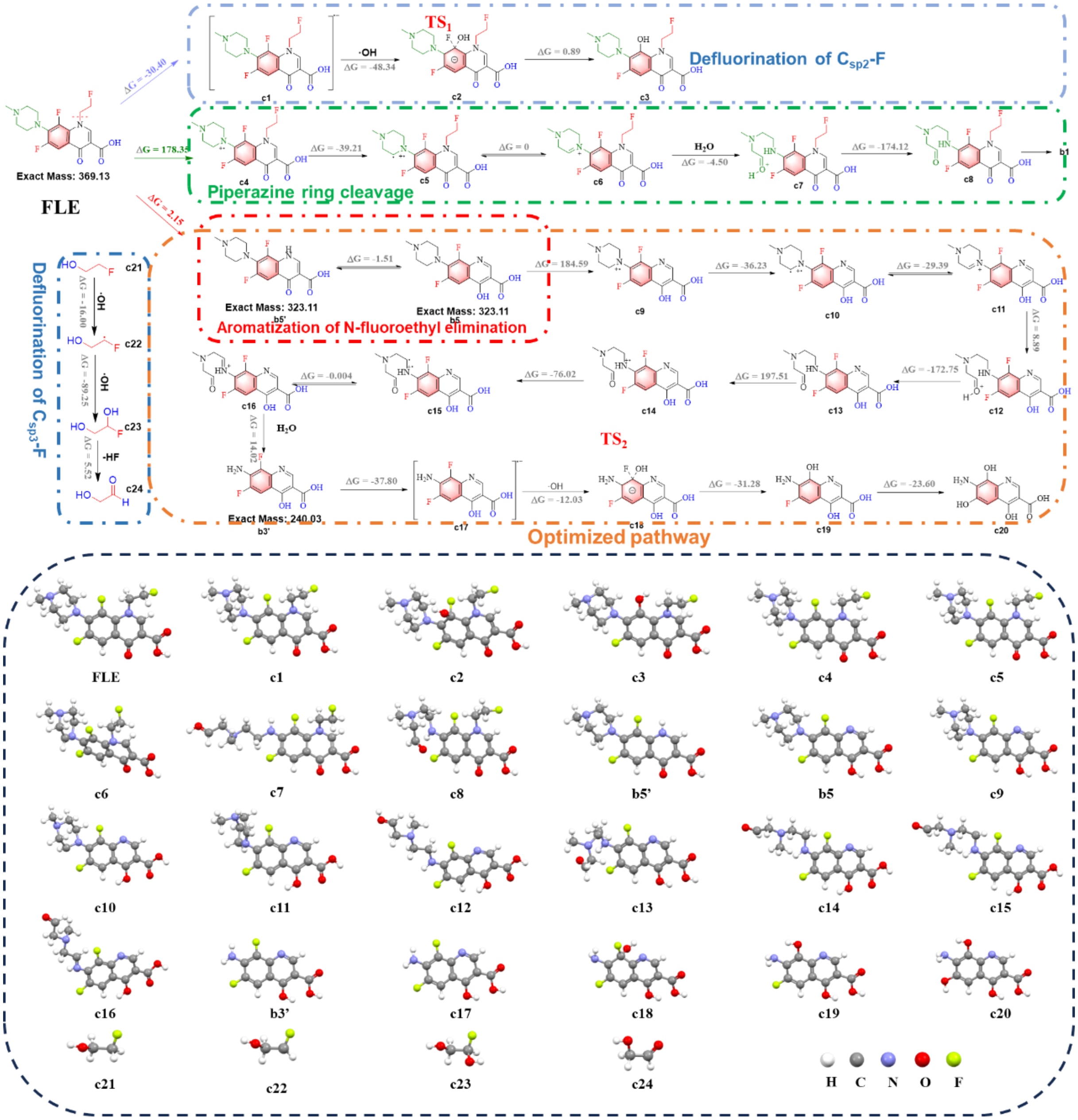

Fig. S30. The defluorination pathways and calculated intermediates of FLE.

Supplementary Notes 4: Similar to the previous C_{sp2}-F bond destruction process, the aromatic ring of FLE accepts an electron and then reacts with •OH to produce phenolic hydroxyl structure, achieving defluorination of C_{sp2}-F bonds, which is thermodynamically permitted. In the second pathway, piperazine undergoes oxidative ring opening, in which the nitrogen atom in piperazine loses electrons to form an imine cation, and then hydrolyzes, leading to the breaking of the C-N bond. This pathway has been speculated and mentioned in the literature,[12-15] even though the reaction energy barrier for the initial electron loss process is high. In the third pathway, FLE

eliminates the β-fluoroethyl group and then rearranges protons to form a benzopyridine skeleton structure with a low free energy change of 2.15 kcal mol^{-1}. These paths are intertwined and occur simultaneously or successively, allowing FLE to be degraded into various small fragments, as confirmed by LC-MS analysis. Considering the C-N bond energies in N-ethyl and piperazine, the C-N bond energy in ethyl is lower (100 kcal/mol) than that of C-N bonds in piperazine (151.58 and 141.96 kcal mol^{-1}) (**Table S4**), making the aromatization pathway feasible, which is consistent with the results of LC-MS analysis. After aromatization, the C-N bond energy of piperazine decreases to 138.14 and 138.03kcal mol^{-1}, which is beneficial to the subsequent C-N dissociation process. In addition, the destruction of 2-fluoroethanol fragment by •OH is thermodynamically favorable.

Table S4. The C-C bond energies of FLE and its intermediates.

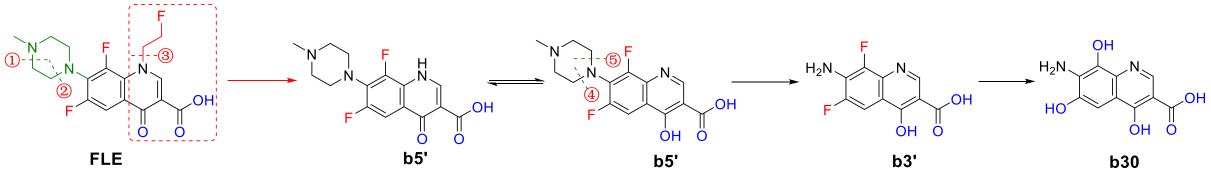

	①	②	③	④	⑤
C-N bond energy （kcal/mol）	151.58	141.96	100.00	138.03	138.14

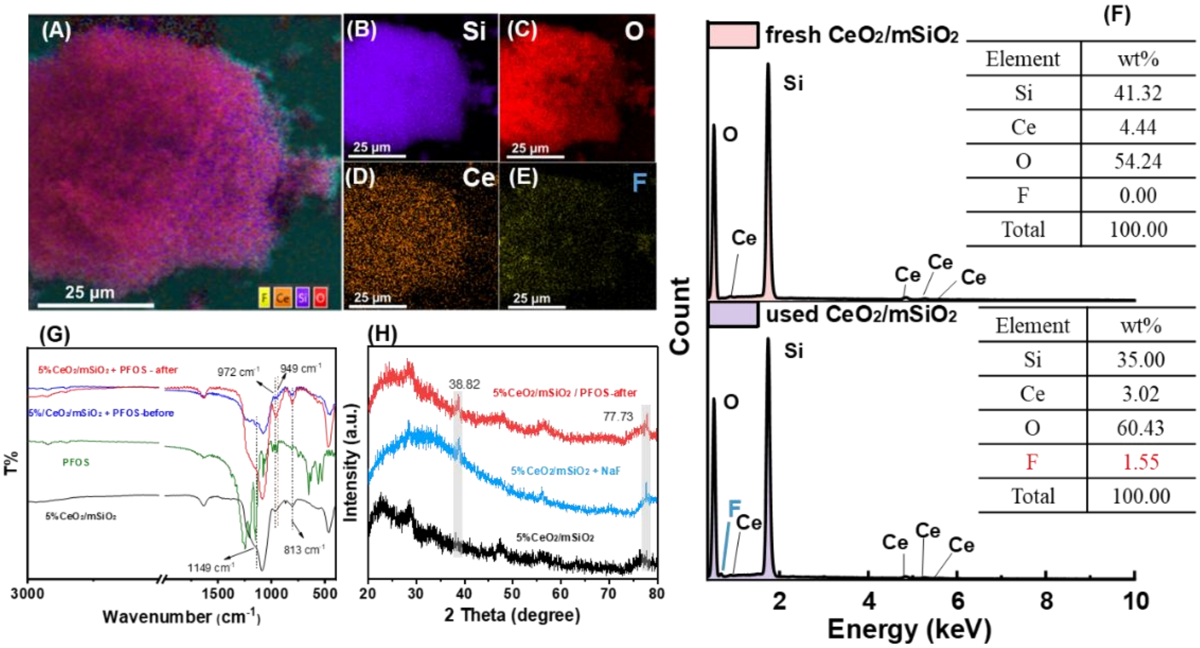

Fig. S31. SEM (A) and the related EDS-mapping of Si (B), O (C), Ce (D), and F (E) elements of $CeO_2/mSiO_2$ catalyst after the degradation of PFOS. (F) Elemental contents of $CeO_2/mSiO_2$ before and after photodegradation of PFOS. (G) FTIR spectra of $CeO_2/mSiO_2$, PFOS, $CeO_2/mSiO_2$ mixed with PFOS before and after photodegradation. (K) XRD patterns of $CeO_2/mSiO_2$, $CeO_2/mSiO_2$ soaked in NaF solution, $CeO_2/mSiO_2$ with PFOS after photodegradation.

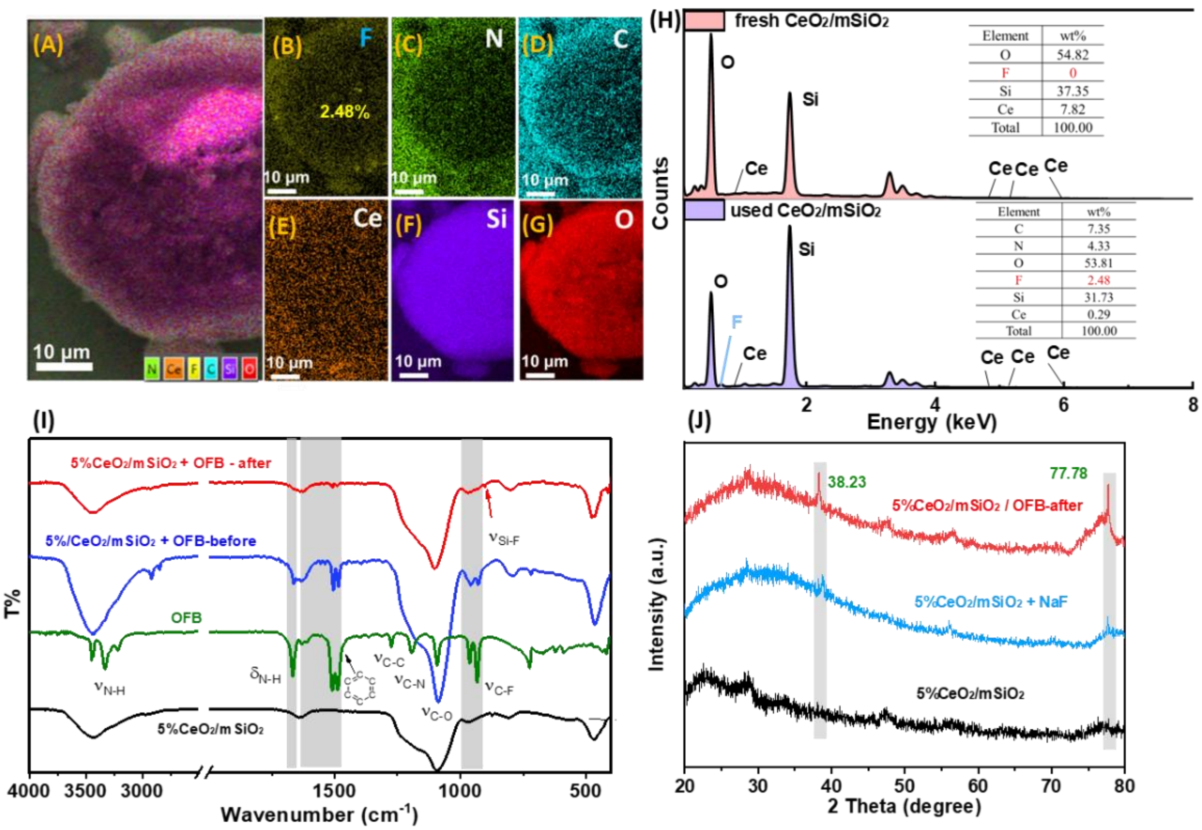

Fig. S32. SEM (A) and the related EDS-mapping of F (B), N (C), C (D), Ce (E), Si (F) and O (G) elements of 5%CeO_2/mSiO_2 catalyst after the degradation of OFB. (H) Elemental contents of 5%CeO_2/mSiO_2 before and after photodegradation of OFB. (I) FTIR spectra of CeO_2/mSiO_2, OFB, CeO_2/mSiO_2 mixed with OFB before and after photodegradation. (J) XRD patterns of CeO_2/mSiO_2, CeO_2/mSiO_2 soaked in NaF solution, CeO_2/mSiO_2 mixed OFB after photodegradation.

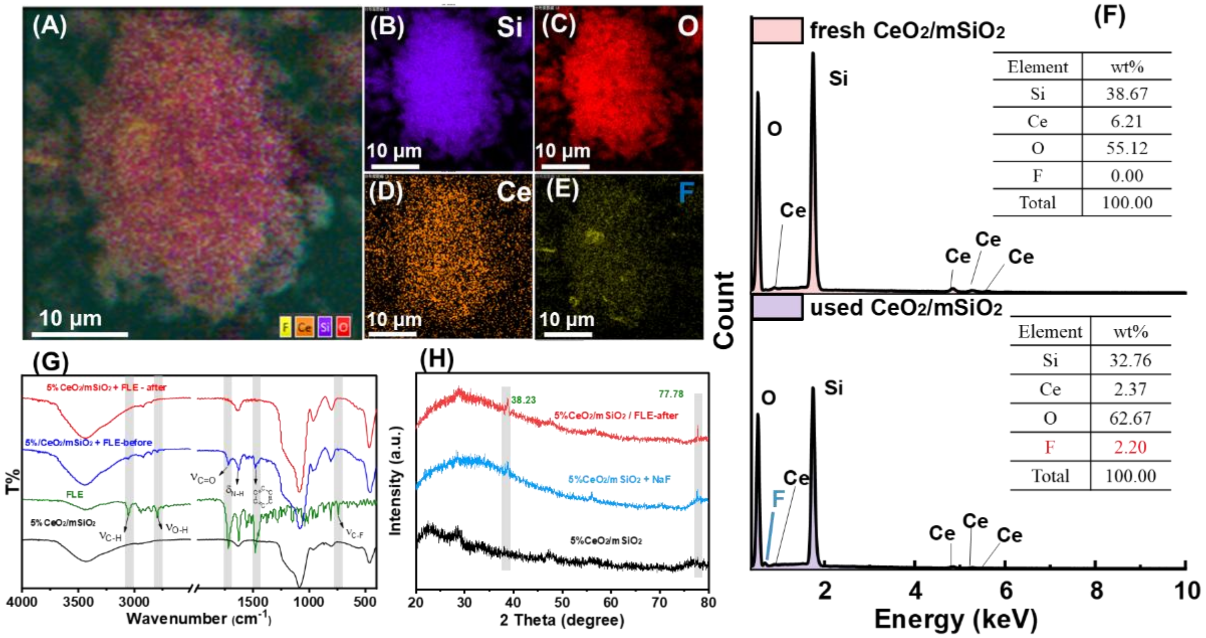

Fig. S33. SEM (A) and the related EDS-mapping of Si (B), O (C), Ce (D), and F (E) elements of 5%CeO_2/mSiO_2 catalyst after the degradation of FLE. (F) Elemental contents of 5%CeO_2/mSiO_2 before and after photodegradation of FLE. (G) FTIR spectra of CeO_2/mSiO_2, FLE, CeO_2/mSiO_2 mixed with FLE before and after photodegradation. (H) XRD patterns of CeO_2/mSiO_2, CeO_2/mSiO_2 soaked in NaF solution, CeO_2/mSiO_2 with FLE after photodegradation.

Supplementary Notes 5: In FTIR spectra, the characteristic signals of organofluorine, attributed to the stretching vibration (ν) of C-F, N-H, aromatic ring, C-N, and the bending vibration (δ) of N-H, are obviously attenuated or even disappeared after illumination. Interestingly, CeO_2/mSiO_2 with organofluorine after illumination shows a weak signal at a lower wavenumber than C-F, which is assigned to the formation of Si-F bonds.[16] The XRD of CeO_2/mSiO_2 soaked with sodium fluoride (NaF) solution exhibits new diffraction peaks at 38.83° and 77.73°, indicating the chemical adsorption of F^- on the catalyst

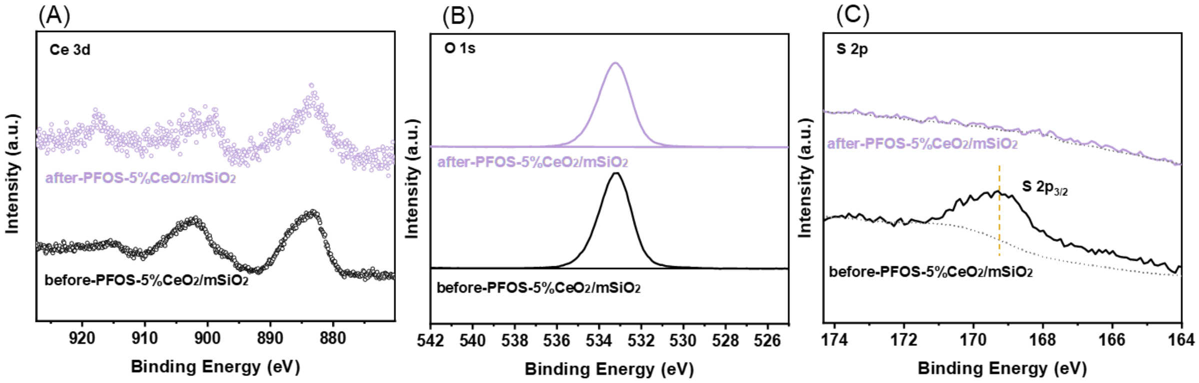

Fig. S34. High-resolution XPS of Ce 3d (A), O 1s (B) and S 2p (C) of 5%CeO_2/mSiO_2 before and after photodegradation of PFOS.

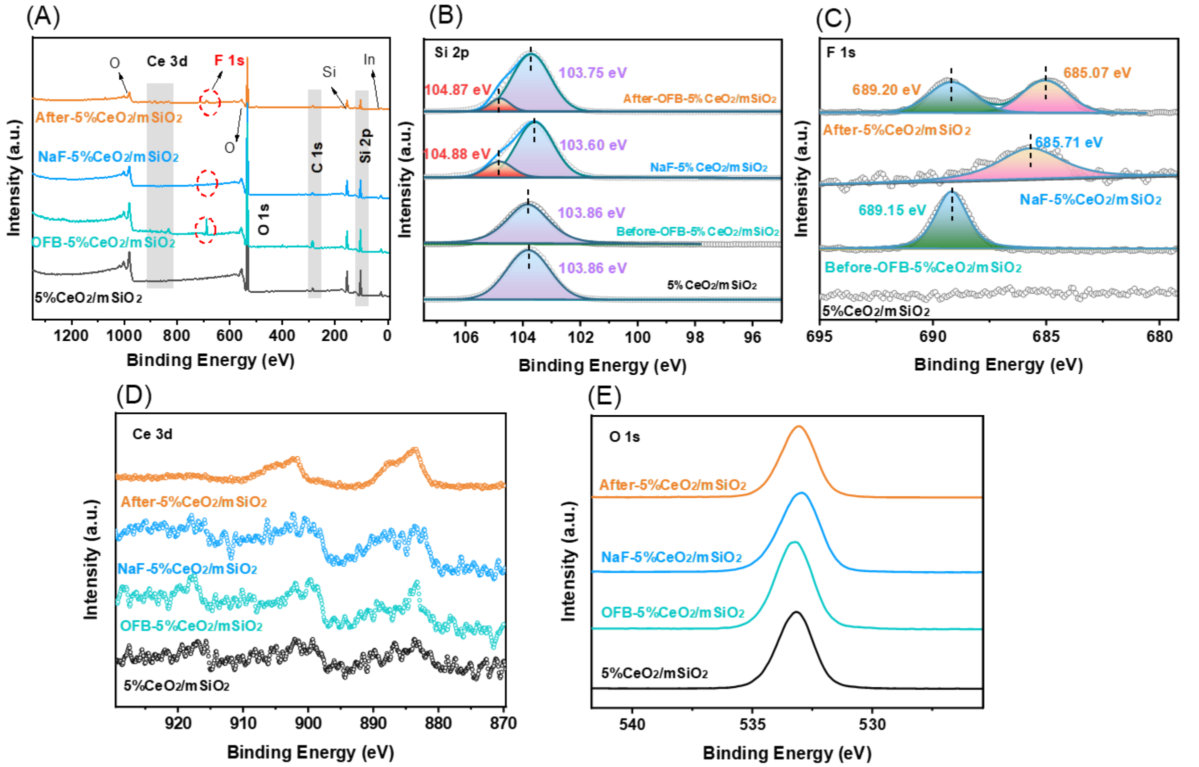

Fig. S35. XPS spectra (A) and high-resolution XPS spectra of Si 2p (B), F 1s (F), Ce 3d (D) and O 1s (E) of CeO_2/mSiO_2 before and after degradation of OFB. CeO_2/mSiO_2 soaked in NaF solution was measured as a reference.

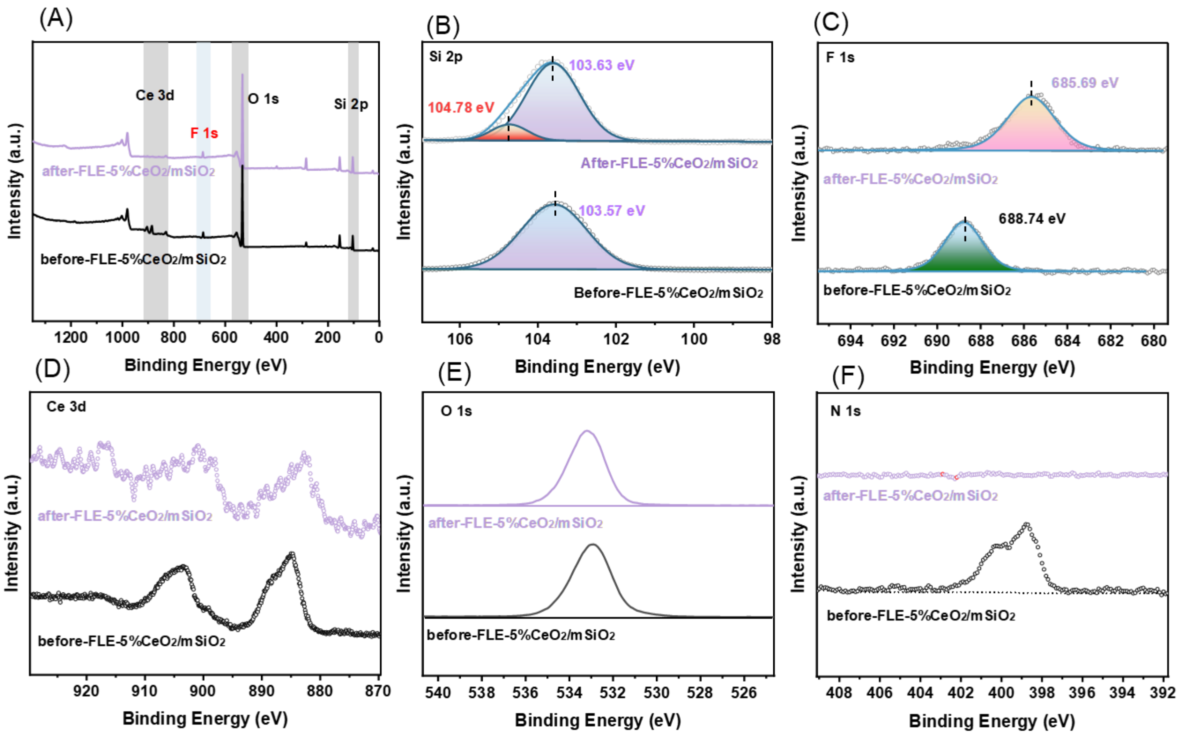

Fig. S36. XPS spectra (A) and high-resolution XPS spectra of Si 2p (B), F 1s (F), Ce 3d (D) and O 1s (E) of $CeO_2/mSiO_2$ before and after degradation of FLE. $CeO_2/mSiO_2$ soaked in NaF solution was measured as a reference.

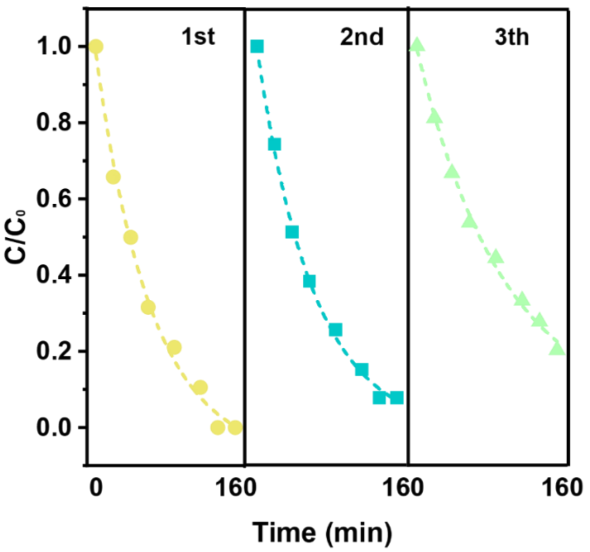

Fig. S37. Recycle experiments of photodegradation of OFB by 5%$CeO_2/mSiO_2$.

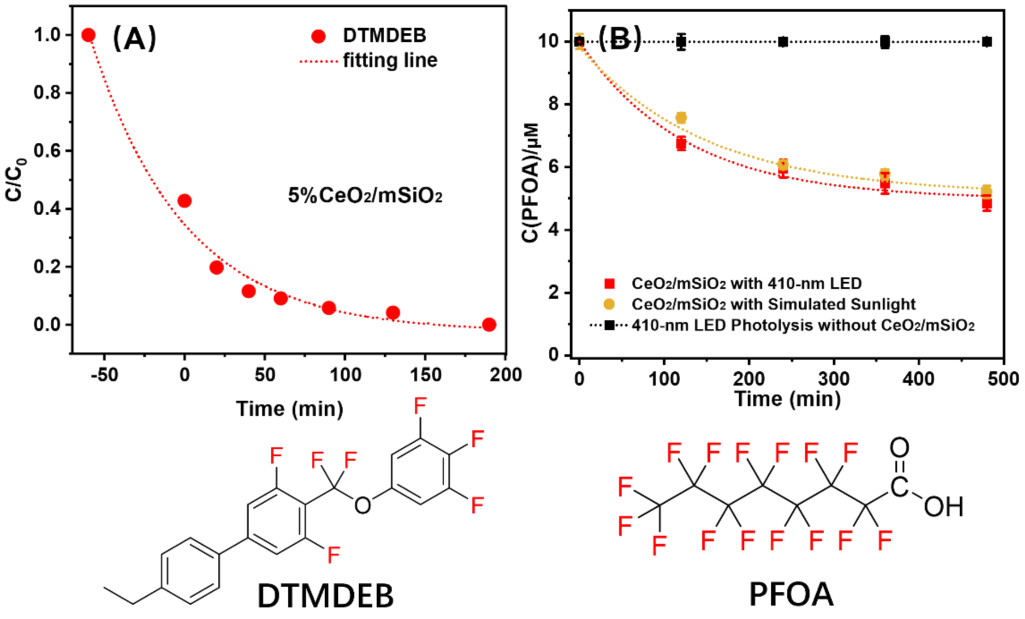

Fig. S38. Photodegradation of DTMEB (A) and PFOA (B) by CeO_2/mSiO_2 catalysts.

Supplementary Reference

1 You, X. *et al.* Synthesis of phosphotungstic acid-supported bimodal mesoporous silica-based catalyst for defluorination of aqueous perfluorooctanoic acid under vacuum UV irradiation. *Chem. Eng. J.* **335**, 812-821 (2018).

2 Yin, L. *et al.* A facile fabrication of highly dispersed CeO_2/SiO_2 aerogel composites with high adsorption desulfurization performance. *Chem. Eng. J.* **428**, 132581 (2022).

3 Xu, L. *et al.* Chiral Skeletons of Mesoporous Silica Nanospheres to Mitigate Alzheimer's β-Amyloid Aggregation. *J. Am. Chem. Soc.* **145**, 7810-7819 (2023).

4 Lin, J., Wu, Y., Khayambashi, A., Wang, X. & Wei, Y. Preparation of a novel CeO_2/SiO_2 adsorbent and its adsorption behavior for fluoride ion. *Adsorpt. Sci. Technol.* **36**, 743-761 (2017).

5 Tan, X. *et al.* Indium-modified Ga_2O_3 hierarchical nanosheets as efficient photocatalysts for the degradation of perfluorooctanoic acid. *Environ. Sci. Nano* **7**, 2229-2239 (2020).

6 Jiang, F., Zhao, H. T., Chen, H., Xu, C. M. & Chen, J. Enhancement of photocatalytic decomposition of perfluorooctanoic acid on CeO_2/In_2O_3. *RSC Adv.* **6**, 72015-72021 (2016).

7 Xu, B. *et al.* Improved photocatalysis of perfluorooctanoic acid in water and wastewater by Ga_2O_3/UV system assisted by peroxymonosulfate. *Chemosphere* **239**, 124722 (2020).

8 Han, J. Y. *et al.* A bifunctional single-atom catalyst assisted by Fe_2O_3 for efficiently electrocatalytic perfluorooctanoic acid degradation by integrating reductive and oxidative processes. *Chem. Eng. J.* **470**, 144270 (2023).

9 Khoshyan, A. *et al.* Degradation of per- and poly-fluoroalkyl substances (PFAS) using ultrasonication: Effect of reactor materials. *J. Water Process. Eng.* **63**, 105511 (2024).

10 Lei, Y.-J., Tian, Y., Sobhani, Z., Naidu, R. & Fang, C. Synergistic degradation of PFAS in water and soil by dual -frequency ultrasonic activated persulfate. *Chem. Eng. J.* **388**, 124215 (2020).

11 Liu, Z. *et al.* Near-Quantitative Defluorination of Perfluorinated and Fluorotelomer Carboxylates and Sulfonates with Integrated Oxidation and Reduction. *Environ. Sci. Technol.* **55**, 7052-7062 (2021).

12 Wang, J. *et al.* Oxidation of selected fluoroquinolones by ferrate(VI) in water: Kinetics, mechanism, effects of constituents, and reaction pathways. *Environ. Res.* **243**, 117845 (2024).

13 Ma, L. *et al.* Photodegradation of fleroxacin by g-C_3N_4/PPy/Ag and HPLC-MS/MS analysis of degradation pathways. *RSC Adv.* **13**, 11912 (2023).

14 Gong, Z. *et al.* Rare earth lanthanum doped catalyst for photocatalytic degradation of fleroxacin. *J. Environ. Chem. Eng.* **11**, 111176 (2023).

15 He, G. *et al.* Reaction of fleroxacin with chlorine and chlorine dioxide in drinking water distribution systems: Kinetics, transformation mechanisms and toxicity evaluations. *Chem. Eng. J.* **374**, 1191-1203 (2019).

16 Chang, K. M. *et al.* Influences of deposition temperature on thermal stability and moisture resistance of chemical vapor deposited fluorinated silicon oxide by using indirect fluorinating precursor. *Appl. Phys. Lett.* **69**, 1238-1240 (1996).

Supplementary Data S1. XYZ coordinates of optimized DFT structures and corresponding energies

PhF

G_{corr}[M062X/6-31G(d)] = 0.064856 a. u.
E[M062X/6-311G(2d)] = -331.442000 a. u.
G = G_{corr} + E = -331.377144 a. u.
C -2.15346530 0.99009900 0.00000000
C -0.75830530 0.99009900 0.00000000
C -0.06076730 2.19785000 0.00000000
C -0.75842130 3.40635900 -0.00119900
C -2.15324630 3.40628100 -0.00167800
C -2.85084730 2.19807500 -0.00068200
H -2.70322430 0.03778200 0.00045000
H -0.20879730 0.03758600 0.00131500
H -0.20822130 4.35850200 -0.00125800
H -2.70336830 4.35856200 -0.00263100
H -3.95045130 2.19825800 -0.00086200
F 1.28923247 2.19794821 0.00077832

[PhF]$^{\cdot+}$

G_{corr}[M062X/6-31G(d)] = 0.063109 a. u.
E[M062X/6-311G(2d)] = -331.095283 a. u.
G = G_{corr} + E = -331.032174 a. u.
C -2.15346530 0.99009900 0.00000000
C -0.75830530 0.99009900 0.00000000
C -0.06076730 2.19785000 0.00000000
C -0.75842130 3.40635900 -0.00119900
C -2.15324630 3.40628100 -0.00167800
C -2.85084730 2.19807500 -0.00068200
H -2.70322430 0.03778200 0.00045000
H -0.20879730 0.03758600 0.00131500
H -0.20822130 4.35850200 -0.00125800
H -2.70336830 4.35856200 -0.00263100
H -3.95045130 2.19825800 -0.00086200
F 1.28923247 2.19794821 0.00077832

[PhF]$^{\cdot-}$

G_{corr}[M062X/6-31G(d)] = 0.054921 a. u.
E[M062X/6-311G(2d)] = -331.412018 a. u.
G = G_{corr} + E = -331.357097 a. u.
C -2.15346530 0.99009900 0.00000000
C -0.75830530 0.99009900 0.00000000
C -0.06076730 2.19785000 0.00000000
C -0.75842130 3.40635900 -0.00119900
C -2.15324630 3.40628100 -0.00167800
C -2.85084730 2.19807500 -0.00068200
H -2.70322430 0.03778200 0.00045000
H -0.20879730 0.03758600 0.00131500

H -0.20822130 4.35850200 -0.00125800
H -2.70336830 4.35856200 -0.00263100
H -3.95045130 2.19825800 -0.00086200
F 1.28923247 2.19794821 0.00077832

[PhF--OH]$^{\cdot}$-1
G_{corr}[M062X/6-31G(d)] = 0.072014 a. u.
E[M062X/6-311G(2d)] = -407.164455 a. u.
G = G_{corr} + E = -407.092441 a. u.
C 1.58301007 1.18427378 -0.01323035
C 2.33048883 0.01284423 0.02036015
C 1.43080235 -1.19252050 -0.01825991
C 0.04165400 -1.21661100 -0.06052700
C -0.80239364 0.04706520 -0.02726974
C 0.08682389 1.27933017 -0.06213009
H 2.11305707 2.13966378 -0.02358935
H 3.42154952 0.01290956 0.04321283
H 1.96087135 -2.14790050 -0.02840591
H -0.50570500 -2.15622500 -0.10127800
H -0.46052911 2.21890417 -0.10343909
F -1.75536744 -0.00071569 1.28191242
O -2.14883034 -0.00175434 -1.14406197
H -2.24199434 -0.00313634 -2.09654697

[PhF--OH]$^{\cdot}$-2
G_{corr}[M062X/6-31G(d)] = 0.072018 a. u.
E[M062X/6-311G(2d)] = -407.164453 a. u.
G = G_{corr} + E = -407.092435 a. u.
C 1.47328976 1.19124458 -0.01681633
C 2.35897895 -0.01874877 0.02153404
C 1.43080235 -1.19252050 -0.01825991
C 0.04165400 -1.21661100 -0.06052700
C -0.80239364 0.04706520 -0.02726974
C 0.08682389 1.27933017 -0.06213009
H 2.00333676 2.14663458 -0.02717533
H 3.45003964 -0.01868344 0.04438672
H 1.96087135 -2.14790050 -0.02840591
H -0.50570500 -2.15622500 -0.10127800
H -0.46052911 2.21890417 -0.10343909
F -1.75536744 -0.00071569 1.28191242
O -2.14883034 -0.00175434 -1.14406197
H -2.24199434 -0.00313634 -2.09654697

[PhF--OH]$^{-}$-1
G_{corr}[M062X/6-31G(d)] = 0.072085 a. u.
E[M062X/6-311G(2d)] = -407.233171 a. u.
G = G_{corr} + E = -407.161086 a. u.

C 1.58301007 1.18427378 -0.01323035
C 2.33048883 0.01284423 0.02036015
C 1.43080235 -1.19252050 -0.01825991
C 0.04165400 -1.21661100 -0.06052700
C -0.80239364 0.04706520 -0.02726974
C 0.08682389 1.27933017 -0.06213009
H 2.11305707 2.13966378 -0.02358935
H 3.42154952 0.01290956 0.04321283
H 1.96087135 -2.14790050 -0.02840591
H -0.50570500 -2.15622500 -0.10127800
H -0.46052911 2.21890417 -0.10343909
F -1.75536744 -0.00071569 1.28191242
O -2.14883034 -0.00175434 -1.14406197
H -2.24199434 -0.00313634 -2.09654697

$[PhF{-}{-}OH]^{-}$-2
Gcorr[M062X/6-31G(d)] = 0.072087 a. u.
E[M062X/6-311G(2d)] = -407.233182 a. u.
G = Gcorr＋E = -407.161095 a. u.
C 1.47328976 1.19124458 -0.01681633
C 2.35897895 -0.01874877 0.02153404
C 1.43080235 -1.19252050 -0.01825991
C 0.04165400 -1.21661100 -0.06052700
C -0.80239364 0.04706520 -0.02726974
C 0.08682389 1.27933017 -0.06213009
H 2.00333676 2.14663458 -0.02717533
H 3.45003964 -0.01868344 0.04438672
H 1.96087135 -2.14790050 -0.02840591
H -0.50570500 -2.15622500 -0.10127800
H -0.46052911 2.21890417 -0.10343909
F -1.75536744 -0.00071569 1.28191242
O -2.14883034 -0.00175434 -1.14406197
H -2.24199434 -0.00313634 -2.09654697

$[PhF{-}{-}OH]^{-}$-3
G_{corr}[M062X/6-31G(d)] = 0.065814 a. u.
E[M062X/6-311G(2d)] = -407.252754 a. u.
G = G_{corr}＋E = -407.18694 a. u.
C -1.39175405 -0.68617696 0.13415323
C 0.00340595 -0.68617696 0.13415323
C 0.70094395 0.52157404 0.13415323
C 0.00328995 1.73008304 0.13295423
C -1.39153505 1.73000504 0.13247523
C -2.08913605 0.52179904 0.13347123
H 0.55291395 -1.63868996 0.13546823
H 1.80062395 0.52165404 0.13478723
H 0.55348995 2.68222604 0.13289523
H -1.94165705 2.68228604 0.13152223
H -3.40913601 0.52201872 0.13325515

F -2.06669739 -1.85534383 0.13470570
O -4.62427722 0.69057280 0.37309125
H -4.94473180 1.59550863 0.37309125

$C_6H_4F^-$
G_{corr}[M062X/6-31G(d)] = 0.049985 a. u.
E[M062X/6-311G(2d)] = -330.815908 a. u.
G = G_{corr} + E = -330.765923 a. u.
C 0.13293500 0.88821800 -0.00145700
C 1.52367100 0.92950100 0.00012400
C 2.22042100 -0.27954900 0.00152200
C 1.50738200 -1.47961500 0.00197700
C 0.10912400 -1.45331900 0.00092200
C -0.63472900 -0.26443800 -0.00111400
H 2.03852900 1.88723000 -0.00016300
H 3.30834600 -0.27880200 0.00251400
H 2.04188300 -2.42815300 0.00326500
H -0.44226700 -2.39331000 0.00194600
F -0.49739800 2.10565000 -0.00228500

$[PhF]^-$
G_{corr}[M062X/6-31G(d)] = 0.046159 a. u.
E[M062X/6-311G(2d)] = -330.709521 a. u.
G = G_{corr} + E = -330.663362 a. u.
C 0.12827877 0.88122661 -0.00145492
C 1.64738793 0.93323038 0.00026458
C 2.28252443 -0.25170018 0.00153994
C 1.47100886 -1.51322397 0.00197773
C 0.13223061 -1.49902679 0.00099426
C -0.55352688 -0.14251211 -0.00115028
H 2.16224593 1.89095938 -0.00002242
H 3.37044943 -0.25095318 0.00253194
H 2.00550986 -2.46176197 0.00326573
H -0.41916039 -2.43901779 0.00201826
F -0.45112267 1.81336891 -1.29968630

Benzyne
G_{corr}[M062X/6-31G(d)] = 0.048889 a. u.
E[M062X/6-311G(2d)] = -230.847997 a. u.
G = G_{corr} + E = -230.799108 a. u.
C -0.70272700 1.05292600 0.00000000
C 0.78676614 1.09178717 0.00000319
C 1.52209492 -0.05147179 0.00000444
C 0.59743800 -1.23257702 -0.00000206
C -0.62256200 -1.23257800 -0.00000500
C -1.52480661 -0.03048764 -0.00000281
H -1.22545200 2.00574700 0.00000000

H 1.30950814 2.04460017 0.00000619
H 2.60615192 -0.05192779 0.00000644
H -2.60886361 -0.03091964 -0.00000581

PhOH
G_{corr}[M062X/6-31G(d)] = 0.076175 a. u.
E[M062X/6-311G(2d)] = -307.413512 a. u.
G = G_{corr} + E = -307.337337 a. u.
C 4.67003319 -3.58697784 1.19191676
C 6.06519319 -3.58697784 1.19191676
C 6.76273119 -2.37922684 1.19191676
C 6.06507719 -1.17071784 1.19071776
C 4.67025219 -1.17079584 1.19023876
C 3.97265119 -2.37900184 1.19123476
H 4.12027419 -4.53929484 1.19236676
H 6.61470119 -4.53949084 1.19323176
H 6.61527719 -0.21857484 1.19065876
H 4.12013019 -0.21851484 1.18928576
H 2.87304719 -2.37881884 1.19105476
O 8.19273066 -2.37936916 1.19314536
H 8.51396256 -2.37985861 0.28848530

·OH
G_{corr}[M062X/6-31G(d)] = -0.008490 a. u.
E[M062X/6-311G(2d)] = -75.722826 a. u.
G = G_{corr} + E = -75.731316 a. u.
O -1.01479849 0.39609262 -0.00809977
H -1.33525307 1.30102845 -0.00809977

OH^-
Gcorr[M062X/6-31G(d)] = -0.008256 a. u.
E[M062X/6-311G(2d)] = -75.779084 a. u.
G = G_{corr} + E = -75.78734 a. u.
O -2.11726378 0.34201954 0.00000000
H -2.43771837 1.24695537 0.00000000

F·
G_{corr}[M062X/6-31G(d)] = -0.014813 a. u.
E[M062X/6-311G(2d)] = -99.726039 a. u.
G = G_{corr} + E = -99.740852 a. u.
F -1.50990096 0.27225389 -0.00356400

F^-
G_{corr}[M062X/6-31G(d)] = -0.014159 a. u.
E[M062X/6-311G(2d)] = -99.842468 a. u.

$G = G_{corr} + E$ = -99.856627 a. u.
F -1.50990096 0.27225389 -0.00356400

H_2O
G_{corr}[M062X/6-31G(d)] = 0.003885 a. u.
E[M062X/6-311G(2d)] = -76.412607 a. u.
$G = G_{corr} + E$ = -76.408722 a. u.
O -1.00725953 0.68058075 0.00000000
H -0.04725953 0.68058075 0.00000000
H -1.32771412 1.58551658 0.00000000

OFB
G_{corr}[M062X/6-31G(d)] = 0.106545 a. u.
E[M062X/6-311G(2d)] = -1367.846477 a. u.
$G = G_{corr} + E$ = -1367.739932 a. u.
C 0.53911203 -3.22975462 -0.26456418
C 1.93427203 -3.22975462 -0.26456418
C 2.63181003 -2.02200362 -0.26456418
C 1.93415603 -0.81349462 -0.26576318
C 0.53933103 -0.81357262 -0.26624218
C -0.15826997 -2.02177862 -0.26524618
C -1.66004077 0.42073343 -0.01614173
C -0.26594542 0.47522119 -0.01696253
C 0.38388930 1.70928346 -0.02214815
C -0.36043342 2.88960936 -0.02771493
C -1.75419118 2.83505484 -0.02737301
C -2.40407066 1.60053942 -0.02118957
F -1.50826993 -2.02155395 -0.26546716
F -0.13583130 -4.39892150 -0.26401171
F -2.38098271 -0.62654592 0.01953286
F 3.98180980 -2.02190541 -0.26378586
F 2.60959791 0.35538444 -0.26583561
F 1.73285539 1.76210872 -0.02216446
F -3.75304930 1.54803900 -0.02061721
F 0.26884024 4.08396641 -0.03280628
N -2.53866775 4.07822096 -0.03324694
H -2.33536399 4.60456747 0.79236007
H -2.30066051 4.61875264 -0.84020298
N 2.66884473 -4.50305736 -0.26280631
H 3.22426553 -4.56860332 -1.09178849
H 3.26291295 -4.54405393 0.54056299

a1
G_{corr}[M062X/6-31G(d)] = 0.156588 a. u.
E[M062X/6-311G(2d)] = -1271.779735 a. u.
$G = G_{corr} + E$ = -1271.623147 a. u.
C 0.53911203 -3.22975462 -0.26456418

C 1.93427203 -3.22975462 -0.26456418
C 2.63181003 -2.02200362 -0.26456418
C 1.93415603 -0.81349462 -0.26576318
C 0.53933103 -0.81357262 -0.26624218
C -0.15826997 -2.02177862 -0.26524618
C -1.66004077 0.42073343 -0.01614173
C -0.26594542 0.47522119 -0.01696253
C 0.38388930 1.70928346 -0.02214815
C -0.36043342 2.88960936 -0.02771493
C -1.75419118 2.83505484 -0.02737301
C -2.40407066 1.60053942 -0.02118957
F -1.50826993 -2.02155395 -0.26546716
F -2.42522925 -0.69082084 0.02172233
F 2.60959791 0.35538444 -0.26583561
F 1.73285539 1.76210872 -0.02216446
N -2.53866775 4.07822096 -0.03324694
H -2.33536399 4.60456747 0.79236007
H -2.30066051 4.61875264 -0.84020298
N 2.66884473 -4.50305736 -0.26280631
H 3.22426553 -4.56860332 -1.09178849
H 3.26291295 -4.54405393 0.54056299
O -3.83298878 1.54492786 -0.02058329
H -4.17505548 2.10167624 0.68271011
O 0.30613053 4.15474312 -0.03310799
H 0.05270075 4.65380772 0.74683295
O -0.17582794 -4.46820546 -0.26397897
H 0.13007435 -5.01516090 -0.99120902
O 4.06180979 -2.02189959 -0.26373974
H 4.38274904 -2.47444102 -1.04719634

a2

G_{corr}[M062X/6-31G(d)] = 0.131919 a. u.
E[M062X/6-311G(2d)] = -1270.450983 a. u.
G = G_{corr} + E = -1270.319064 a. u.

C 2.95025496 1.40685239 0.10920529
C 3.70746329 0.27009460 -0.47545049
C 3.05887155 -0.93296498 -1.09349289
C 1.55950890 -0.97585957 -1.10195900
C 0.72446135 0.13774958 -0.54287916
C 1.46534672 1.30266433 0.04368924
C -1.37586217 1.29812270 -1.13874781
C -0.62548311 0.13781463 -0.55512426
C -1.41864825 -1.00009058 0.01589307
C -2.90672017 -1.01564693 0.03602927
C -3.63066454 0.15692789 -0.56577894
C -2.87529632 1.32763089 -1.16749689
F 0.78774863 2.31263491 0.54429141
F -0.70126443 2.16887582 -1.91926489
F 0.88652522 -1.98807082 -1.60782672
F -0.74573124 -2.01251600 0.52143435

N -4.89607534 0.17605954 -0.57429464
H -5.23460874 -0.68993101 -0.12846374
N 4.97287543 0.28937151 -0.46743741
H 5.31132180 -0.57659319 -0.91338118
O 3.49331971 2.36336633 0.61020888
O -3.41829568 2.28422459 -1.66842129
O -3.49332098 -1.96001334 0.52599647
O 3.64537075 -1.87740254 -1.58344318
H -1.31129119 0.93776143 -2.14416829
H -1.57663081 -1.92065587 -0.50612328
H 1.79264094 1.60581463 -0.92887356
H 1.65241713 -0.33645728 -1.95485570

a2'

G_{corr}[M062X/6-31G(d)] = 0.133603 a. u.
E[M062X/6-311G(2d)] = -1270.501189 a. u.
G = G_{corr} + E = -1270.367586 a. u.
C 0.40696404 -3.23619973 -0.26484444
C 1.94696404 -3.23619973 -0.26484444
C 2.71716496 -1.90263663 -0.26484444
C 2.02200132 -0.76877332 -0.26606647
C 0.50475003 -0.78505037 -0.26657614
C -0.25134587 -2.08054940 -0.26545749
C -1.70955608 0.26863547 -0.05039493
C -0.19071600 0.32799883 -0.05128917
C 0.52681237 1.69061406 -0.05701498
C -0.19947907 2.80478293 -0.06235212
C -1.70511448 2.70253508 -0.06177229
C -2.42770739 1.38807110 -0.05514314
F -1.56479017 -2.38595843 -0.32940589
F -2.61011489 -0.73463017 0.01997468
F 2.87209678 0.27945563 -0.29852847
F 1.85063688 1.95317453 -0.02460098
N -2.39353269 3.79347678 -0.06692696
H -2.19022893 4.31982329 0.75868005
N 2.59158906 -4.35358785 -0.26330182
H 3.18565728 -4.39458442 0.54006748
O -3.85662551 1.33245954 -0.05453686
H -4.19869221 1.88920792 0.64875654
O 0.46708488 4.06991669 -0.06774518
H 0.21365510 4.56898129 0.71219576
O -0.30797593 -4.47465057 -0.26425923
H -0.00207364 -5.02160601 -0.99148928
O 4.14716472 -1.90253260 -0.26402000
H 4.46810397 -2.35507403 -1.04747660

a3

G_{corr}[M062X/6-31G(d)] = 0.080379 a. u.
E[M062X/6-311G(2d)] = -711.699540 a. u.

$G = G_{corr} + E = -711.619161$ a. u.
C -1.76567606 -0.28344373 0.00000000
C -0.56666661 -0.99677939 0.00000000
C 0.65031675 -0.31547679 0.00000000
C 0.66865073 1.07982926 -0.00119900
C -0.53011070 1.79292660 -0.00167800
C -1.74738085 1.11126519 -0.00068200
O -0.51175550 3.22280826 -0.00291717
H -0.50666694 3.54243957 -0.90812981
O 1.87932090 -1.04653638 0.00082444
H 2.01243332 -1.44861588 0.86234238
O -3.01331142 -0.98223245 0.00058521
H -3.29267598 -1.13998322 -0.90421872
F -2.90746460 1.80170392 -0.00090299
F -0.58479005 -2.34665677 0.00161437
N 1.95149127 1.79761762 -0.00127787
H 2.46302229 1.56614562 0.82622242
H 2.48198216 1.53208092 -0.80630561

a4
G_{corr}[M062X/6-31G(d)] = 0.129075 a. u.
E[M062X/6-311G(2d)] = -1140.160724 a. u.
$G = G_{corr} + E = -1140.031649$ a. u.
C -1.93955912 1.32307613 0.20633075
C -1.29400099 1.06131649 -1.14557707
C -0.37234906 -0.13183728 -1.00178087
C -1.10047247 -1.42962061 -0.70018134
C 1.88135912 -1.23005485 -1.11200463
C 0.95968271 -0.03635298 -1.00243632
C 1.68455782 1.27418650 -0.77561939
C 3.06947942 1.07879366 -0.11912461
C 3.36491674 -0.21637322 0.63470198
C 2.57977315 -1.44899174 0.22910257
F -0.73549504 -2.02169727 0.43618848
F 1.77823439 -2.47969799 -1.61226190
F -0.69293592 2.16609351 -1.65874888
F 0.99261565 2.00241028 0.17461323
N 4.27669357 -0.29430111 1.50833781
H 4.72897644 0.62744050 1.59723315
O 2.54152924 -2.47365711 0.84492906
O 3.86616387 1.98126944 -0.10821872
O -1.94645658 2.37585920 0.78204088
H 2.66328970 -0.97462960 -1.85033822
H 1.79554114 1.88840569 -1.67375187
H -1.19224273 -2.10902893 -1.55120383
H -2.10295733 0.77024692 -1.83971688
O -3.28796634 0.84777389 0.17829952
H -4.23873304 0.71601343 0.19508832
H -0.14349802 -1.59390588 -1.19546940

a8

G_{corr}[M062X/6-31G(d)] = 0.099577 a. u.
E[M062X/6-311G(2d)] = -1367.850231 a. u.
G = G_{corr} + E = -1367.750654 a. u.

C 0.53911203 -3.22975462 -0.26456418
C 1.93427203 -3.22975462 -0.26456418
C 2.63181003 -2.02200362 -0.26456418
C 1.93415603 -0.81349462 -0.26576318
C 0.53933103 -0.81357262 -0.26624218
C -0.15826997 -2.02177862 -0.26524618
C -1.66004077 0.42073343 -0.01614173
C -0.26594542 0.47522119 -0.01696253
C 0.38388930 1.70928346 -0.02214815
C -0.36043342 2.88960936 -0.02771493
C -1.75419118 2.83505484 -0.02737301
C -2.40407066 1.60053942 -0.02118957
F -1.50826993 -2.02155395 -0.26546716
F -0.13583130 -4.39892150 -0.26401171
F -2.38098271 -0.62654592 0.01953286
F 3.98180980 -2.02190541 -0.26378586
F 2.60959791 0.35538444 -0.26583561
F 1.73285539 1.76210872 -0.02216446
F -3.75304930 1.54803900 -0.02061721
F 0.26884024 4.08396641 -0.03280628
N -2.53866775 4.07822096 -0.03324694
H -2.33536399 4.60456747 0.79236007
H -2.30066051 4.61875264 -0.84020298
N 2.66884473 -4.50305736 -0.26280631
H 3.22426553 -4.56860332 -1.09178849
H 3.26291295 -4.54405393 0.54056299

a9

G_{corr}[M062X/6-31G(d)] = 0.113691 a. u.
E[M062X/6-311G(2d)] = -1443.653169 a. u.
G = G_{corr} + E = -1443.539478 a. u.

C 0.16583103 -4.12576954 -0.13522608
C 1.56099103 -4.12576954 -0.13522608
C 2.25852903 -2.91801854 -0.13522608
C 1.56087503 -1.70950954 -0.13642508
C 0.16605003 -1.70958754 -0.13690408
C -0.53155097 -2.91779354 -0.13590808
C -2.03332177 -0.47528149 0.11319637
C -0.63922642 -0.42079373 0.11237557
C 0.01060830 0.81326854 0.10718995
C -0.73371442 1.99359444 0.10162317
C -2.12747218 1.93903992 0.10196509
C -2.77735166 0.70452450 0.10814853

F -1.88155093 -2.91756887 -0.13612906
F -2.79851025 -1.58683576 0.15106043
F 3.07731372 -2.22296893 1.07757962
F 2.23631691 -0.54063048 -0.13649751
F 1.35957439 0.86609380 0.10717364
N -2.91194875 3.18220604 0.09609116
H -2.70864499 3.70855255 0.92169817
H -2.67394151 3.72273772 -0.71086488
N 2.38204506 -5.31999374 -0.38139439
H 2.10434876 -5.73980835 0.48268921
H 3.26039418 -5.77984811 -0.25086935
O 2.90736579 -2.21504204 -1.60060483
H 3.41355716 -2.24827964 -2.41562971
F -4.12633030 0.65202408 0.10872089
F -0.10444076 3.18795148 0.09653182
F -0.50911230 -5.29493642 -0.13467361

a10

G_{corr}[M062X/6-31G(d)] = 0.119404 a. u.
E[M062X/6-311G(2d)] = -1343.827339 a. u.
G = G_{corr} + E = -1343.707935 a. u.
C 0.53911203 -3.22975462 -0.26456418
C 1.93427203 -3.22975462 -0.26456418
C 2.63181003 -2.02200362 -0.26456418
C 1.93415603 -0.81349462 -0.26576318
C 0.53933103 -0.81357262 -0.26624218
C -0.15826997 -2.02177862 -0.26524618
C -1.66004077 0.42073343 -0.01614173
C -0.26594542 0.47522119 -0.01696253
C 0.38388930 1.70928346 -0.02214815
C -0.36043342 2.88960936 -0.02771493
C -1.75419118 2.83505484 -0.02737301
C -2.40407066 1.60053942 -0.02118957
F -1.50826993 -2.02155395 -0.26546716
F -0.13583130 -4.39892150 -0.26401171
F -2.42522925 -0.69082084 0.02172233
F 3.98180980 -2.02190541 -0.26378586
F 2.60959791 0.35538444 -0.26583561
F 1.73285539 1.76210872 -0.02216446
F 0.26884024 4.08396641 -0.03280628
N -2.53866775 4.07822096 -0.03324694
H -2.33536399 4.60456747 0.79236007
H -2.30066051 4.61875264 -0.84020298
N 2.66884473 -4.50305736 -0.26280631
H 3.22426553 -4.56860332 -1.09178849
H 3.26291295 -4.54405393 0.54056299
O -3.83298878 1.54492786 -0.02058329
H -4.17505548 2.10167624 0.68271011

a11
G_{corr}[M062X/6-31G(d)] = 0.131744 a. u.
E[M062X/6-311G(2d)] = -1319.806067 a. u.
G = G_{corr} + E = -1319.674323 a. u.
C 0.53911203 -3.22975462 -0.26456418
C 1.93427203 -3.22975462 -0.26456418
C 2.63181003 -2.02200362 -0.26456418
C 1.93415603 -0.81349462 -0.26576318
C 0.53933103 -0.81357262 -0.26624218
C -0.15826997 -2.02177862 -0.26524618
C -1.66004077 0.42073343 -0.01614173
C -0.26594542 0.47522119 -0.01696253
C 0.38388930 1.70928346 -0.02214815
C -0.36043342 2.88960936 -0.02771493
C -1.75419118 2.83505484 -0.02737301
C -2.40407066 1.60053942 -0.02118957
F -1.50826993 -2.02155395 -0.26546716
F -0.13583130 -4.39892150 -0.26401171
F -2.42522925 -0.69082084 0.02172233
F 3.98180980 -2.02190541 -0.26378586
F 2.60959791 0.35538444 -0.26583561
F 1.73285539 1.76210872 -0.02216446
N -2.53866775 4.07822096 -0.03324694
H -2.33536399 4.60456747 0.79236007
H -2.30066051 4.61875264 -0.84020298
N 2.66884473 -4.50305736 -0.26280631
H 3.22426553 -4.56860332 -1.09178849
H 3.26291295 -4.54405393 0.54056299
O -3.83298878 1.54492786 -0.02058329
H -4.17505548 2.10167624 0.68271011
O 0.30613053 4.15474312 -0.03310799
H 0.05270075 4.65380772 0.74683295

a12
G_{corr}[M062X/6-31G(d)] = 0.144476 a. u.
E[M062X/6-311G(2d)] = -1295.794109 a. u.
G = G_{corr} + E = -1295.649633 a. u.
C 0.53911203 -3.22975462 -0.26456418
C 1.93427203 -3.22975462 -0.26456418
C 2.63181003 -2.02200362 -0.26456418
C 1.93415603 -0.81349462 -0.26576318
C 0.53933103 -0.81357262 -0.26624218
C -0.15826997 -2.02177862 -0.26524618
C -1.66004077 0.42073343 -0.01614173
C -0.26594542 0.47522119 -0.01696253
C 0.38388930 1.70928346 -0.02214815
C -0.36043342 2.88960936 -0.02771493
C -1.75419118 2.83505484 -0.02737301
C -2.40407066 1.60053942 -0.02118957

F -1.50826993 -2.02155395 -0.26546716
F -2.42522925 -0.69082084 0.02172233
F 3.98180980 -2.02190541 -0.26378586
F 2.60959791 0.35538444 -0.26583561
F 1.73285539 1.76210872 -0.02216446
N -2.53866775 4.07822096 -0.03324694
H -2.33536399 4.60456747 0.79236007
H -2.30066051 4.61875264 -0.84020298
N 2.66884473 -4.50305736 -0.26280631
H 3.22426553 -4.56860332 -1.09178849
H 3.26291295 -4.54405393 0.54056299
O -3.83298878 1.54492786 -0.02058329
H -4.17505548 2.10167624 0.68271011
O 0.30613053 4.15474312 -0.03310799
H 0.05270075 4.65380772 0.74683295
O -0.17582794 -4.46820546 -0.26397897
H 0.13007435 -5.01516090 -0.99120902

PFeSA

G_{corr}[M062X/6-31G(d)] = 0.006734 a. u.
E[M062X/6-311G(2d)] = -1199.233398 a. u.
G = G_{corr}＋E = -1199.226664 a. u.
C -1.71374131 0.46279491 0.06733308
C -0.21349131 0.46279491 0.06733308
F 0.26387810 1.09433783 1.16084668
F 0.26385573 1.09423792 -1.02624797
F 0.26449142 -0.79975509 0.06740560
F -2.19121519 1.72553744 0.06733308
F -2.19147970 -0.16842214 -1.02620753
S -2.34374554 -0.36955253 1.50910020
O -1.80454284 0.41176660 2.88303647
O -1.80442120 -1.95003318 1.51959661
O -4.01334367 -0.35130086 1.47733615

$C_2F_5^-$

G_{corr}[M062X/6-31G(d)] = -0.007640 a. u.
E[M062X/6-311G(2d)] = -575.414211 a. u.
G = G_{corr}＋E = -575.421851 a. u.
C -0.66243194 -0.13611615 0.00000000
C 0.83781806 -0.13611615 0.00000000
F -1.13990582 1.12662638 0.00000000
F -1.14024414 -0.76739089 1.09347506
F -1.14017033 -0.76733320 -1.09354062
F 1.31516510 0.49532686 -1.09358105
F 1.31580079 -1.39866615 0.00007251

$\cdot C_2F_5$

G_{corr}[M062X/6-31G(d)] = -0.006292 a. u.
E[M062X/6-311G(2d)] = -575.358140 a. u.
G = G_{corr} + E = -575.364432 a. u.
C -0.66243194 -0.13611615 0.00000000
C 0.83781806 -0.13611615 0.00000000
F -1.13990582 1.12662638 0.00000000
F -1.14024414 -0.76739089 1.09347506
F -1.14017033 -0.76733320 -1.09354062
F 1.31516510 0.49532686 -1.09358105
F 1.31580079 -1.39866615 0.00007251

C_2F_5OH
G_{corr}[M062X/6-31G(d)] = 0.010668 a. u.
E[M062X/6-311G(2d)] = -651.262506 a. u.
G = G_{corr} + E = -651.251838 a. u.
C -0.42635113 0.73502721 -0.01116440
C 1.07389887 0.73502721 -0.01116440
F 1.55126829 1.36657013 1.08234920
F 1.55124592 1.36647022 -1.10474545
F 1.55188161 -0.52752279 -0.01109188
F -0.90382500 1.99776974 -0.01116440
F -0.90416332 0.10375247 1.08231066
O -0.93239994 0.06640471 -1.16950742
H -1.89218057 0.07650826 -1.15164542

C_2F_3H
G_{corr}[M062X/6-31G(d)] = 0.002306 a. u.
E[M062X/6-311G(2d)] = -376.119715 a. u.
G = G_{corr} + E = -376.117409 a. u.
C -1.87840291 0.40834845 0.00000000
C -0.37815291 0.40834845 0.00000000
H -2.27351691 1.45327945 0.00000000
F 0.09921651 1.03989137 1.09351359
F 0.09982982 -0.85420155 0.00007251
F -2.35621510 -0.22292629 1.09347506

C_2F_3OH
G_{corr}[M062X/6-31G(d)] = 0.005092 a. u.
E[M062X/6-311G(2d)] = -451.494384 a. u.
G = G_{corr} + E = -451.489292 a. u.
C -0.95281307 0.06352087 0.00000000
C 0.37310293 0.06352087 0.00000000
F -1.68245358 1.19935745 0.00000000
F -1.68249397 -1.07228976 -0.00002704
F 1.10278382 1.19933150 0.00003196
O 1.14598139 -1.13962454 -0.00002473
H 1.93731851 -1.01214999 -0.52835778

C_2F_4

G_{corr}[M062X/6-31G(d)] = -0.006924 a. u.
E[M062X/6-311G(2d)] = -475.514485 a. u.
G = G_{corr}＋E= -475.521409 a. u.
C -0.95281307 0.06352087 0.00000000
C 0.37310293 0.06352087 0.00000000
F -1.68245358 1.19935745 0.00000000
F -1.68249397 -1.07228976 -0.00002704
F 1.10278382 1.19933150 0.00003196
F 1.10274344 -1.07231571 -0.00002335

C_2F_5H

G_{corr}[M062X/6-31G(d)] = 0.008568 a. u.
E[M062X/6-311G(2d)] = -576.031978 a. u.
G = G_{corr}＋E= -576.02341 a. u.
C -0.42635113 0.73502721 -0.01116440
C 1.07389887 0.73502721 -0.01116440
H -0.82168713 0.21268521 -0.91608640
F 1.55126829 1.36657013 1.08234920
F 1.55124592 1.36647022 -1.10474545
F 1.55188161 -0.52752279 -0.01109188
F -0.90382500 1.99776974 -0.01116440
F -0.90416332 0.10375247 1.08231066

CF_3COF

G_{corr}[M062X/6-31G(d)] = -0.003263 a. u.
E[M062X/6-311G(2d)] = -550.746941 a. u.
G = G_{corr}＋E= -550.750204 a. u.
C -1.89655173 0.35390199 0.00000000
C -0.38940173 0.35390199 0.00000000
F -2.36784107 1.61896577 0.00000000
F -2.37233409 -0.27862716 1.09363523
F -2.37221629 -0.27848220 -1.09377030
O 0.00062088 0.93002449 0.94067275
F 0.11198788 -0.89953666 -0.00006449

$\cdot CF_3$

G_{corr}[M062X/6-31G(d)] = -0.012942 a. u.
E[M062X/6-311G(2d)] = -337.560525 a. u.
G = G_{corr}＋E= -337.573467 a. u.
C -0.68058077 0.53539019 0.00000000
F -0.23057298 1.17178043 1.10227059
F -0.23059621 -0.73740747 0.00000000
F -2.03058077 0.53540683 0.00000000

CF_3COOH
G_{corr}[M062X/6-31G(d)] = 0.008937 a. u.
E[M062X/6-311G(2d)] = -526.652242 a. u.
G = G_{corr} + E = -526.643305 a. u.
C -1.89655173 0.35390199 0.00000000
C -0.38940173 0.35390199 0.00000000
H 1.28661727 -1.01883001 -0.00013000
F -2.36784107 1.61896577 0.00000000
F -2.37233409 -0.27862716 1.09363523
F -2.37221629 -0.27848220 -1.09377030
O 0.08729257 1.05805171 1.14971114
O 0.17034527 -1.04542601 -0.00007200

CF_3OH
G_{corr}[M062X/6-31G(d)] = 0.002507 a. u.
E[M062X/6-311G(2d)] = -413.469615 a. u.
G = G_{corr} + E = -413.467108 a. u.
C -1.06170599 0.37205081 0.00000000
F -1.53917987 1.63479334 0.00000000
F -1.53951818 -0.25922393 1.09347506
F -1.53944438 -0.25916624 -1.09354062
O 0.36829401 0.37205081 0.00000000
H 0.68874859 -0.53288499 0.00022270

CHF_2COF
G_{corr}[M062X/6-31G(d)] = 0.005851 a. u.
E[M062X/6-311G(2d)] = -451.490263 a. u.
G = G_{corr} + E = -451.484412 a. u.
C -0.42635113 0.73502721 -0.01116440
C 1.07389887 0.73502721 -0.01116440
H -0.82168713 0.21268521 -0.91608640
F 1.55126829 1.36657013 1.08234920
F -0.90382500 1.99776974 -0.01116440
F -0.90416332 0.10375247 1.08231066
O 1.48815058 -0.35918279 -0.01110155

CO_2
G_{corr}[M062X/6-31G(d)] = -0.008735 a. u.
E[M062X/6-311G(2d)] = -188.542456 a. u.
G = G_{corr} + E = -188.551191 a. u.
C -0.91651543 0.33575317 0.00000000
O 0.34188457 0.33575317 0.00000000
O -2.17491543 0.33575317 0.00000000

F_2

G_{corr}[M062X/6-31G(d)] = -0.016919 a. u.
E[M062X/6-311G(2d)] = -199.502857 a. u.
G = G_{corr}＋E= -199.519776 a. u.
F -1.27516281 0.24105754 -0.01669280
F -2.43516281 0.24105754 -0.01669280

SO_3
G_{corr}[M062X/6-31G(d)] = -0.013923 a. u.
E[M062X/6-311G(2d)] = -623.780103 a. u.
G = G_{corr}＋E= -623.794026 a. u.
S -2.34836691 0.92534991 0.00000000
O -1.61608892 2.19951360 0.00000000
O -3.80836691 0.92534991 0.00000000
O -1.62087245 -0.34049045 0.00000000

C_2F_5COOH
G_{corr}[M062X/6-31G(d)] = 0.018255 a. u.
E[M062X/6-311G(2d)] = -764.592805 a. u.
G = G_{corr}＋E= -764.57455 a. u.
C 1.06627800 0.47314000 0.18725900
C 2.35673600 -0.34061900 -0.05889500
F 1.11874700 1.00270600 1.42007900
F 1.00298700 1.45933300 -0.72232900
F 2.25170700 -1.51878500 0.56989000
F 2.49647400 -0.53394100 -1.37783900
C 3.60445900 0.38830200 0.47564500
O 3.56196598 1.54562270 0.16131531
O 3.65682440 0.27342169 1.90006080
H 4.57135612 0.30281778 2.19052182
F -0.04523251 -0.28621113 0.08513046

PFBA
G_{corr}[M062X/6-31G(d)] = 0.026822 a. u.
E[M062X/6-311G(2d)] = -1002.384933 a. u.
G = G_{corr} + E = -1002.358111 a. u.
C -0.20543100 -0.39565400 0.07041100
C 1.06627800 0.47314000 0.18725900
C 2.35673600 -0.34061900 -0.05889500
F -0.34507100 -1.11059200 1.19768300
F -0.06507100 -1.22264300 -0.97743900
F 1.11874700 1.00270600 1.42007900
F 1.00298700 1.45933300 -0.72232900
F 2.25170700 -1.51878500 0.56989000
F 2.49647400 -0.53394100 -1.37783900
C 3.60445900 0.38830200 0.47564500
O 3.56196598 1.54562270 0.16131531
O 3.65682440 0.27342169 1.90006080
H 4.57135612 0.30281778 2.19052182
F -1.31667481 0.34930508 -0.11034620

PFPeA
G_{corr}[M062X/6-31G(d)] = 0.035745 a. u.
E[M062X/6-311G(2d)] = -1240.177076 a. u.
G = G_{corr} + E = -1240.141331 a. u.
C -1.47663100 0.45653700 -0.13636500
C -0.20543100 -0.39565400 0.07041100
C 1.06627800 0.47314000 0.18725900
C 2.35673600 -0.34061900 -0.05889500
F -1.41642400 1.52248900 0.67843000
F -1.51694700 0.86732700 -1.41378300
F -0.34507100 -1.11059200 1.19768300
F -0.06507100 -1.22264300 -0.97743900
F 1.11874700 1.00270600 1.42007900
F 1.00298700 1.45933300 -0.72232900
F 2.25170700 -1.51878500 0.56989000
F 2.49647400 -0.53394100 -1.37783900
C 3.60445900 0.38830200 0.47564500
O 3.56196598 1.54562270 0.16131531
O 3.65682440 0.27342169 1.90006080
H 4.57135612 0.30281778 2.19052182
F -2.60435727 -0.23384152 0.13586830

PFHxA
G_{corr}[M062X/6-31G(d)] = 0.045077 a. u.
E[M062X/6-311G(2d)] = -1477.969270 a. u.
G = G_{corr} + E = -1477.924193 a. u.
C -2.76700300 -0.33341100 0.17513100
C -1.47663100 0.45653700 -0.13636500
C -0.20543100 -0.39565400 0.07041100

C 1.06627800 0.47314000 0.18725900
C 2.35673600 -0.34061900 -0.05889500
F -2.93123500 -0.38243200 1.50705400
F -2.63740800 -1.58106600 -0.30566200
F -1.41642400 1.52248900 0.67843000
F -1.51694700 0.86732700 -1.41378300
F -0.34507100 -1.11059200 1.19768300
F -0.06507100 -1.22264300 -0.97743900
F 1.11874700 1.00270600 1.42007900
F 1.00298700 1.45933300 -0.72232900
F 2.25170700 -1.51878500 0.56989000
F 2.49647400 -0.53394100 -1.37783900
C 3.60445900 0.38830200 0.47564500
O 3.56196598 1.54562270 0.16131531
O 3.65682440 0.27342169 1.90006080
H 4.57135612 0.30281778 2.19052182
F -3.86048732 0.23136106 -0.37968839

PFHpA

G_{corr}[M062X/6-31G(d)] = 0.053990 a. u.
E[M062X/6-311G(2d)] = -1715.761451 a. u.
G = G_{corr} + E = -1715.707461 a. u.
C -4.01532900 0.31133500 -0.45825300
C -2.76700300 -0.33341100 0.17513100
C -1.47663100 0.45653700 -0.13636500
C -0.20543100 -0.39565400 0.07041100
C 1.06627800 0.47314000 0.18725900
C 2.35673600 -0.34061900 -0.05889500
F -3.95472000 1.64260300 -0.28021100
F -4.02649100 0.03205100 -1.76884700
F -2.93123500 -0.38243200 1.50705400
F -2.63740800 -1.58106600 -0.30566200
F -1.41642400 1.52248900 0.67843000
F -1.51694700 0.86732700 -1.41378300
F -0.34507100 -1.11059200 1.19768300
F -0.06507100 -1.22264300 -0.97743900
F 1.11874700 1.00270600 1.42007900
F 1.00298700 1.45933300 -0.72232900
F 2.25170700 -1.51878500 0.56989000
F 2.49647400 -0.53394100 -1.37783900
C 3.60445900 0.38830200 0.47564500
O 3.56196598 1.54562270 0.16131531
O 3.65682440 0.27342169 1.90006080
H 4.57135612 0.30281778 2.19052182
F -5.17506645 -0.13054803 0.07301758

PFOA

G_{corr}[M062X/6-31G(d)] = 0.063538 a. u.
E[M062X/6-311G(2d)] = -1953.553632 a. u.

G = G_{corr} + E = -1953.490094 a. u.
C -5.33802900 -0.19264000 0.14767000
C -4.01532900 0.31133500 -0.45825300
C -2.76700300 -0.33341100 0.17513100
C -1.47663100 0.45653700 -0.13636500
C -0.20543100 -0.39565400 0.07041100
C 1.06627800 0.47314000 0.18725900
C 2.35673600 -0.34061900 -0.05889500
F -6.34588800 0.21301800 -0.61599700
F -5.50070900 0.28963900 1.37323400
F -5.33309200 -1.52265800 0.19500300
F -3.95472000 1.64260300 -0.28021100
F -4.02649100 0.03205100 -1.76884700
F -2.93123500 -0.38243200 1.50705400
F -2.63740800 -1.58106600 -0.30566200
F -1.41642400 1.52248900 0.67843000
F -1.51694700 0.86732700 -1.41378300
F -0.34507100 -1.11059200 1.19768300
F -0.06507100 -1.22264300 -0.97743900
F 1.11874700 1.00270600 1.42007900
F 1.00298700 1.45933300 -0.72232900
F 2.25170700 -1.51878500 0.56989000
F 2.49647400 -0.53394100 -1.37783900
C 3.60445900 0.38830200 0.47564500
O 3.56196598 1.54562270 0.16131531
O 3.65682440 0.27342169 1.90006080
H 4.57135612 0.30281778 2.19052182

TFA
G_{corr}[M062X/6-31G(d)] = 0.008937 a. u.
E[M062X/6-311G(2d)] = -526.798793 a. u.
G = G_{corr} + E = -526.789856 a. u.
C -1.89655173 0.35390199 0.00000000
C -0.38940173 0.35390199 0.00000000
H 1.28661727 -1.01883001 -0.00013000
F -2.36784107 1.61896577 0.00000000
F -2.37233409 -0.27862716 1.09363523
F -2.37221629 -0.27848220 -1.09377030
O 0.08729257 1.05805171 1.14971114
O 0.17034527 -1.04542601 -0.00007200

CF_3OH
G_{corr}[M062X/6-31G(d)] = 0.002507 a. u.
E[M062X/6-311G(2d)] = -413.477545 a. u.
G = G_{corr} + E = -413.475038 a. u.
C -1.06170599 0.37205081 0.00000000
F -1.53917987 1.63479334 0.00000000
F -1.53951818 -0.25922393 1.09347506

F -1.53944438 -0.25916624 -1.09354062
O 0.36829401 0.37205081 0.00000000
H 0.68874859 -0.53288499 0.00022270

HF
G_{corr}[M062X/6-31G(d)] = -0.007126 a. u.
E[M062X/6-311G(2d)] = -100.436926 a. u.
G = G_{corr} + E = -100.444052 a. u.
F 0.13611615 0.04535650 -0.00118770
H -0.74388385 0.04535650 -0.00118770

HSO_4^-
G_{corr}[M062X/6-31G(d)] = -0.000423 a. u.
E[M062X/6-311G(2d)] = -699.731203 a. u.
G = G_{corr} + E = -699.731626 a. u.
S -1.07948477 0.35390199 0.02826730
O -0.52280846 1.14114029 -1.33528224
O -0.52280846 1.14114029 1.39181685
O -0.52283721 -1.22059586 0.02826730
O -2.74948477 0.35392257 0.02826730
H -3.06994724 1.25885390 0.03002481

INT1
G_{corr}[M062X/6-31G(d)] = 0.059775 a. u.
E[M062X/6-311G(2d)] = -2626.111009 a. u.
G = G_{corr} + E = -2626.051234 a. u.
C -5.33802900 -0.19264000 0.14767000
C -4.01532900 0.31133500 -0.45825300
C -2.76700300 -0.33341100 0.17513100
C -1.47663100 0.45653700 -0.13636500
C -0.20543100 -0.39565400 0.07041100
C 1.06627800 0.47314000 0.18725900
C 2.35673600 -0.34061900 -0.05889500
C 3.60445900 0.38830200 0.47564500
F -6.34588800 0.21301800 -0.61599700
F -5.50070900 0.28963900 1.37323400
F -5.33309200 -1.52265800 0.19500300
F -3.95472000 1.64260300 -0.28021100
F -4.02649100 0.03205100 -1.76884700
F -2.93123500 -0.38243200 1.50705400
F -2.63740800 -1.58106600 -0.30566200
F -1.41642400 1.52248900 0.67843000
F -1.51694700 0.86732700 -1.41378300
F -0.34507100 -1.11059200 1.19768300
F -0.06507100 -1.22264300 -0.97743900
F 1.11874700 1.00270600 1.42007900
F 1.00298700 1.45933300 -0.72232900

F 2.25170700 -1.51878500 0.56989000
F 2.49647400 -0.53394100 -1.37783900
F 3.65323600 0.28129400 1.80245100
F 3.55697000 1.68169100 0.12435900
S 5.19618600 -0.30164400 -0.16792400
O 6.24275900 0.34495900 0.58532200
O 5.04508400 -1.72535600 -0.27382900
O 5.18203800 0.30588200 -1.65221400

INT2

G_{corr}[M062X/6-31G(d)] = 0.047101 a. u.
E[M062X/6-311G(2d)] = -2002.197142 a. u.
G = G_{corr}＋E= -2002.150041 a. u.
C -5.33802900 -0.19264000 0.14767000
C -4.01532900 0.31133500 -0.45825300
C -2.76700300 -0.33341100 0.17513100
C -1.47663100 0.45653700 -0.13636500
C -0.20543100 -0.39565400 0.07041100
C 1.06627800 0.47314000 0.18725900
C 2.35673600 -0.34061900 -0.05889500
F -6.34588800 0.21301800 -0.61599700
F -5.50070900 0.28963900 1.37323400
F -5.33309200 -1.52265800 0.19500300
F -3.95472000 1.64260300 -0.28021100
F -4.02649100 0.03205100 -1.76884700
F -2.93123500 -0.38243200 1.50705400
F -2.63740800 -1.58106600 -0.30566200
F -1.41642400 1.52248900 0.67843000
F -1.51694700 0.86732700 -1.41378300
F -0.34507100 -1.11059200 1.19768300
F -0.06507100 -1.22264300 -0.97743900
F 1.11874700 1.00270600 1.42007900
F 1.00298700 1.45933300 -0.72232900
F 2.25170700 -1.51878500 0.56989000
F 2.49647400 -0.53394100 -1.37783900
F 3.65323600 0.28129400 1.80245100
F 3.55697000 1.68169100 0.12435900
C 3.60445900 0.38830200 0.47564500

INT3

G_{corr}[M062X/6-31G(d)] = 0.062596 a. u.
E[M062X/6-311G(2d)] = -2002.787669 a. u.
G = G_{corr}＋E= -2002.725073 a. u.
C -5.33802900 -0.19264000 0.14767000
C -4.01532900 0.31133500 -0.45825300
C -2.76700300 -0.33341100 0.17513100
C -1.47663100 0.45653700 -0.13636500
C -0.20543100 -0.39565400 0.07041100

C 1.06627800 0.47314000 0.18725900
C 2.35673600 -0.34061900 -0.05889500
F -6.34588800 0.21301800 -0.61599700
F -5.50070900 0.28963900 1.37323400
F -5.33309200 -1.52265800 0.19500300
F -3.95472000 1.64260300 -0.28021100
F -4.02649100 0.03205100 -1.76884700
F -2.93123500 -0.38243200 1.50705400
F -2.63740800 -1.58106600 -0.30566200
F -1.41642400 1.52248900 0.67843000
F -1.51694700 0.86732700 -1.41378300
F -0.34507100 -1.11059200 1.19768300
F -0.06507100 -1.22264300 -0.97743900
F 1.11874700 1.00270600 1.42007900
F 1.00298700 1.45933300 -0.72232900
F 2.25170700 -1.51878500 0.56989000
F 2.49647400 -0.53394100 -1.37783900
F 3.65323600 0.28129400 1.80245100
F 3.55697000 1.68169100 0.12435900
C 3.60445900 0.38830200 0.47564500
H 4.04912787 -0.21909133 -0.28477787

INT4

G_{corr}[M062X/6-31G(d)] = 0.046656 a. u.
E[M062X/6-311G(2d)] = -1902.297984 a. u.
G = G_{corr}＋E= -1902.251328 a. u.
C -5.33802900 -0.19264000 0.14767000
C -4.01532900 0.31133500 -0.45825300
C -2.76700300 -0.33341100 0.17513100
C -1.47663100 0.45653700 -0.13636500
C -0.20543100 -0.39565400 0.07041100
C 1.06627800 0.47314000 0.18725900
C 2.35673600 -0.34061900 -0.05889500
F -6.34588800 0.21301800 -0.61599700
F -5.50070900 0.28963900 1.37323400
F -5.33309200 -1.52265800 0.19500300
F -3.95472000 1.64260300 -0.28021100
F -4.02649100 0.03205100 -1.76884700
F -2.93123500 -0.38243200 1.50705400
F -2.63740800 -1.58106600 -0.30566200
F -1.41642400 1.52248900 0.67843000
F -1.51694700 0.86732700 -1.41378300
F -0.34507100 -1.11059200 1.19768300
F -0.06507100 -1.22264300 -0.97743900
F 1.11874700 1.00270600 1.42007900
F 1.00298700 1.45933300 -0.72232900
F 2.25170700 -1.51878500 0.56989000
F 3.39440849 1.58672241 0.05471563
C 3.44189749 0.29333341 0.40600163
F 4.00292831 -0.47300397 -0.55341040

INT5

G_{corr}[M062X/6-31G(d)] = 0.055846 a. u.
E[M062X/6-311G(2d)] = -1803.054545 a. u.
G = G_{corr} + E = -1802.998699 a. u.
C -5.33802900 -0.19264000 0.14767000
C -4.01532900 0.31133500 -0.45825300
C -2.76700300 -0.33341100 0.17513100
C -1.47663100 0.45653700 -0.13636500
C -0.20543100 -0.39565400 0.07041100
C 1.06627800 0.47314000 0.18725900
C 2.35673600 -0.34061900 -0.05889500
F -6.34588800 0.21301800 -0.61599700
F -5.50070900 0.28963900 1.37323400
F -5.33309200 -1.52265800 0.19500300
F -3.95472000 1.64260300 -0.28021100
F -4.02649100 0.03205100 -1.76884700
F -2.93123500 -0.38243200 1.50705400
F -2.63740800 -1.58106600 -0.30566200
F -1.41642400 1.52248900 0.67843000
F -1.51694700 0.86732700 -1.41378300
F -0.34507100 -1.11059200 1.19768300
F -0.06507100 -1.22264300 -0.97743900
F 1.11874700 1.00270600 1.42007900
F 1.00298700 1.45933300 -0.72232900
F 2.25170700 -1.51878500 0.56989000
F 3.39440849 1.58672241 0.05471563
C 3.44189749 0.29333341 0.40600163
H 3.88656636 -0.31405992 -0.35442124

INT6

G_{corr}[M062X/6-31G(d)] = 0.056642 a. u.
E[M062X/6-311G(2d)] = -2625.901376 a. u.
G = G_{corr} + E = -2625.844734 a. u.
C -5.33802900 -0.19264000 0.14767000
C -4.01532900 0.31133500 -0.45825300
C -2.76700300 -0.33341100 0.17513100
C -1.47663100 0.45653700 -0.13636500
C -0.20543100 -0.39565400 0.07041100
C 1.06627800 0.47314000 0.18725900
C 2.35673600 -0.34061900 -0.05889500
C 3.60445900 0.38830200 0.47564500
F -6.34588800 0.21301800 -0.61599700
F -5.50070900 0.28963900 1.37323400
F -5.33309200 -1.52265800 0.19500300
F -3.95472000 1.64260300 -0.28021100
F -4.02649100 0.03205100 -1.76884700
F -2.93123500 -0.38243200 1.50705400

F -2.63740800 -1.58106600 -0.30566200
F -1.41642400 1.52248900 0.67843000
F -1.51694700 0.86732700 -1.41378300
F -0.34507100 -1.11059200 1.19768300
F -0.06507100 -1.22264300 -0.97743900
F 1.11874700 1.00270600 1.42007900
F 1.00298700 1.45933300 -0.72232900
F 2.25170700 -1.51878500 0.56989000
F 2.49647400 -0.53394100 -1.37783900
F 3.65323600 0.28129400 1.80245100
F 3.55697000 1.68169100 0.12435900
S 5.19618600 -0.30164400 -0.16792400
O 6.24275900 0.34495900 0.58532200
O 5.04508400 -1.72535600 -0.27382900
O 5.18203800 0.30588200 -1.65221400

INT7
G_{corr}[M062X/6-31G(d)] = 0.048368 a. u.
E[M062X/6-311G(2d)] = -2002.118382 a. u.
G = G_{corr} + E = -2002.070014 a. u.
C -5.33802900 -0.19264000 0.14767000
C -4.01532900 0.31133500 -0.45825300
C -2.76700300 -0.33341100 0.17513100
C -1.47663100 0.45653700 -0.13636500
C -0.20543100 -0.39565400 0.07041100
C 1.06627800 0.47314000 0.18725900
C 2.35673600 -0.34061900 -0.05889500
F -6.34588800 0.21301800 -0.61599700
F -5.50070900 0.28963900 1.37323400
F -5.33309200 -1.52265800 0.19500300
F -3.95472000 1.64260300 -0.28021100
F -4.02649100 0.03205100 -1.76884700
F -2.93123500 -0.38243200 1.50705400
F -2.63740800 -1.58106600 -0.30566200
F -1.41642400 1.52248900 0.67843000
F -1.51694700 0.86732700 -1.41378300
F -0.34507100 -1.11059200 1.19768300
F -0.06507100 -1.22264300 -0.97743900
F 1.11874700 1.00270600 1.42007900
F 1.00298700 1.45933300 -0.72232900
F 2.25170700 -1.51878500 0.56989000
F 2.49647400 -0.53394100 -1.37783900
F 3.65323600 0.28129400 1.80245100
F 3.55697000 1.68169100 0.12435900
C 3.60445900 0.38830200 0.47564500

INT8
G_{corr}[M062X/6-31G(d)] = 0.065802 a. u.
E[M062X/6-311G(2d)] = -2078.024117 a. u.

G = G_{corr} + E = -2077.958315 a. u.
C -5.33802900 -0.19264000 0.14767000
C -4.01532900 0.31133500 -0.45825300
C -2.76700300 -0.33341100 0.17513100
C -1.47663100 0.45653700 -0.13636500
C -0.20543100 -0.39565400 0.07041100
C 1.06627800 0.47314000 0.18725900
C 2.35673600 -0.34061900 -0.05889500
F -6.34588800 0.21301800 -0.61599700
F -5.50070900 0.28963900 1.37323400
F -5.33309200 -1.52265800 0.19500300
F -3.95472000 1.64260300 -0.28021100
F -4.02649100 0.03205100 -1.76884700
F -2.93123500 -0.38243200 1.50705400
F -2.63740800 -1.58106600 -0.30566200
F -1.41642400 1.52248900 0.67843000
F -1.51694700 0.86732700 -1.41378300
F -0.34507100 -1.11059200 1.19768300
F -0.06507100 -1.22264300 -0.97743900
F 1.11874700 1.00270600 1.42007900
F 1.00298700 1.45933300 -0.72232900
F 2.25170700 -1.51878500 0.56989000
F 2.49647400 -0.53394100 -1.37783900
F 3.65323600 0.28129400 1.80245100
F 3.55697000 1.68169100 0.12435900
C 3.60445900 0.38830200 0.47564500
O 4.19873609 -0.42344796 -0.54062108
H 5.15497228 -0.37657849 -0.46980014

INT9
G_{corr}[M062X/6-31G(d)] = 0.078111 a. u.
E[M062X/6-311G(2d)] = -2054.004904 a. u.
G = G_{corr} + E = -2053.926793 a. u.
C -5.33802900 -0.19264000 0.14767000
C -4.01532900 0.31133500 -0.45825300
C -2.76700300 -0.33341100 0.17513100
C -1.47663100 0.45653700 -0.13636500
C -0.20543100 -0.39565400 0.07041100
C 1.06627800 0.47314000 0.18725900
C 2.35673600 -0.34061900 -0.05889500
F -6.34588800 0.21301800 -0.61599700
F -5.50070900 0.28963900 1.37323400
F -5.33309200 -1.52265800 0.19500300
F -3.95472000 1.64260300 -0.28021100
F -4.02649100 0.03205100 -1.76884700
F -2.93123500 -0.38243200 1.50705400
F -2.63740800 -1.58106600 -0.30566200
F -1.41642400 1.52248900 0.67843000
F -1.51694700 0.86732700 -1.41378300

F -0.34507100 -1.11059200 1.19768300
F -0.06507100 -1.22264300 -0.97743900
F 1.11874700 1.00270600 1.42007900
F 1.00298700 1.45933300 -0.72232900
F 2.25170700 -1.51878500 0.56989000
F 2.49647400 -0.53394100 -1.37783900
F 3.65323600 0.28129400 1.80245100
C 3.60445900 0.38830200 0.47564500
O 4.19873609 -0.42344796 -0.54062108
H 5.15497228 -0.37657849 -0.46980014
O 3.55382149 1.76744250 0.10106879
H 4.42927808 2.15334249 0.18016967

INT10

G_{corr}[M062X/6-31G(d)] = 0.049952 a. u.
E[M062X/6-311G(2d)] = -1977.563443 a. u.
G = G_{corr}＋E = -1977.513491 a. u.
C -5.33802900 -0.19264000 0.14767000
C -4.01532900 0.31133500 -0.45825300
C -2.76700300 -0.33341100 0.17513100
C -1.47663100 0.45653700 -0.13636500
C -0.20543100 -0.39565400 0.07041100
C 1.06627800 0.47314000 0.18725900
C 2.35673600 -0.34061900 -0.05889500
F -6.34588800 0.21301800 -0.61599700
F -5.50070900 0.28963900 1.37323400
F -5.33309200 -1.52265800 0.19500300
F -3.95472000 1.64260300 -0.28021100
F -4.02649100 0.03205100 -1.76884700
F -2.93123500 -0.38243200 1.50705400
F -2.63740800 -1.58106600 -0.30566200
F -1.41642400 1.52248900 0.67843000
F -1.51694700 0.86732700 -1.41378300
F -0.34507100 -1.11059200 1.19768300
F -0.06507100 -1.22264300 -0.97743900
F 1.11874700 1.00270600 1.42007900
F 1.00298700 1.45933300 -0.72232900
F 2.25170700 -1.51878500 0.56989000
F 2.49647400 -0.53394100 -1.37783900
F 3.65323600 0.28129400 1.80245100
C 3.60445900 0.38830200 0.47564500
O 3.56196598 1.54562270 0.16131531

INT11

G_{corr}[M062X/6-31G(d)] = 0.063538 a. u.
E[M062X/6-311G(2d)] = -1953.553632 a. u.
G = G_{corr}＋E = -1953.490094 a. u.
C -5.33802900 -0.19264000 0.14767000

C -4.01532900 0.31133500 -0.45825300
C -2.76700300 -0.33341100 0.17513100
C -1.47663100 0.45653700 -0.13636500
C -0.20543100 -0.39565400 0.07041100
C 1.06627800 0.47314000 0.18725900
C 2.35673600 -0.34061900 -0.05889500
F -6.34588800 0.21301800 -0.61599700
F -5.50070900 0.28963900 1.37323400
F -5.33309200 -1.52265800 0.19500300
F -3.95472000 1.64260300 -0.28021100
F -4.02649100 0.03205100 -1.76884700
F -2.93123500 -0.38243200 1.50705400
F -2.63740800 -1.58106600 -0.30566200
F -1.41642400 1.52248900 0.67843000
F -1.51694700 0.86732700 -1.41378300
F -0.34507100 -1.11059200 1.19768300
F -0.06507100 -1.22264300 -0.97743900
F 1.11874700 1.00270600 1.42007900
F 1.00298700 1.45933300 -0.72232900
F 2.25170700 -1.51878500 0.56989000
F 2.49647400 -0.53394100 -1.37783900
C 3.60445900 0.38830200 0.47564500
O 3.56196598 1.54562270 0.16131531
O 3.65682440 0.27342169 1.90006080
H 4.57135612 0.30281778 2.19052182

PFOS

G_{corr}[M062X/6-31G(d)] = 0.070764 a. u.
E[M062X/6-311G(2d)] = -2626.584408 a. u.
G = G_{corr} + E = -2626.513644 a. u.
C -3.78010116 1.92117509 0.51812089
C -2.27295360 1.92117398 0.52083431
C -1.70099804 0.49132544 0.52076074
C -0.19427855 0.52734618 0.52073641
C 0.41168687 -0.88842424 0.52058931
C 1.91711502 -0.81640342 0.52104020
C 2.55674415 -2.21728690 0.52083264
C 4.06002096 -2.10930710 0.52140451
F -4.25139043 3.18623889 0.51818345
F -4.25785134 1.28785886 1.61044196
F -4.25379541 1.28957912 -0.57695914
F -1.82488271 2.58514854 1.60751285
F -1.82106364 2.58677429 -0.56326517
F -2.16150829 -0.15244292 1.61437521
F -2.16143163 -0.15219770 -0.57303032
F 0.23975005 1.20262023 1.60615067
F 0.23962209 1.20277726 -0.56463131
F -0.03364144 -1.54321529 1.61394716
F -0.03289935 -1.54256736 -0.57345816
F 2.33454753 -0.13114800 1.60670825

F 2.33508207 -0.13059619 -0.56407361
F 2.12711057 -2.88261881 1.61410682
F 2.12800085 -2.88178409 -0.57329838
F 4.46087534 -1.41435482 1.60715637
F 4.46155918 -1.41362244 -0.56362540
S 4.71858455 -3.46493592 0.52114583
O 5.95293094 -3.34666165 0.52156021
O 4.38660548 -4.11209466 1.52542441
O 4.33646397 -4.21046345 -0.63776425
H 4.97602715 -4.90898338 -0.79470004

FLE

G_{corr}[M062X/6-31G(d)] = 0.297925 a. u.
E[M062X/6-311G(2d)] = -1347.943174 a. u.
G = G_{corr} + E = -1347.645249 a. u.

C -1.05804500 -2.21068182 -0.06473129
C 0.25811488 -2.43805836 0.04307906
C 1.16399545 -1.24252349 0.05184134
C 0.73998365 0.03723296 -0.01842545
C -0.73609865 0.24673286 -0.18362483
C -1.56204945 -0.80421246 -0.19823585
C 2.61656658 -1.68979109 0.12063326
C 3.00223901 0.76028175 0.00161050
C 3.51656230 -0.49476649 0.01143655
H 3.69225368 1.59532652 -0.05188446
C -3.95874836 -1.18520161 0.46523260
C -3.53350381 0.38085184 -1.31114795
C -4.59557070 0.00578633 1.32051095
H -3.49161549 -1.90369547 1.13660889
C -4.21676818 1.54378244 -0.46386738
H -2.75954242 0.79068725 -1.95720053
H -3.78758238 0.45461589 1.90596932
H -3.42808555 2.05720983 0.08889551
N -3.00089347 -0.60363039 -0.42272353
N -5.16051962 0.96136356 0.43199305
N 1.61600909 1.21543008 0.05458804
C 1.18666988 2.61734869 0.24282297
C 2.28689847 3.54608122 0.71274758
H 0.40018803 2.62588932 1.00116920
H 0.77389516 3.00649641 -0.69166871
H 1.83352249 4.50101184 0.99451278
H 2.82763830 3.13878252 1.57343808
C 5.00429309 -0.54859309 -0.05979323
O 5.69700426 0.44308704 -0.05122863
O 5.52430986 -1.87749259 -0.15203569
H 6.49380819 -1.79309645 -0.19870380
F -1.93717950 -3.21461387 -0.10923972
F -1.24989949 1.48649367 -0.32796877
F 3.17815695 3.77626770 -0.31146662

O 2.94629492 -2.85345859 0.23364681
C -6.49629664 0.70263966 -0.07769673
H -6.89580546 1.61387663 -0.52589495
H -7.14662965 0.39628054 0.74278048
H -6.49270627 -0.09229441 -0.84206350
H -4.70834045 2.23697925 -1.14908800
H -4.32335935 -0.07625785 -1.91971499
H -5.35401011 -0.41145204 1.98597297
H -4.76744863 -1.66559246 -0.09684648
H 0.65544411 -3.44667778 0.09977195

b3'

G_{corr}[M062X/6-31G(d)] = 0.117956 a. u.
E[M062X/6-311G(2d)] = -919.490490 a. u.
G = G_{corr} + E = -919.372534 a. u.
C -1.12164243 -1.95875082 0.11515837
C 0.19958263 -2.23594898 0.12021027
C 1.24648593 -1.18152099 0.09383543
C 0.82901658 0.11003098 0.08842836
C -0.63175057 0.45072850 0.07923400
C -1.55940576 -0.52420428 0.09419445
C 2.68290829 -1.71731412 0.02723781
C 3.03077082 0.70304023 -0.02494842
C 3.57138623 -0.69455323 -0.09178776
H 3.78372926 1.47893129 -0.10214679
N -2.88542593 -0.08897458 0.12065121
N 1.77954113 1.21187170 0.11436132
C 5.03439193 -0.86498000 -0.24495759
O 5.81971706 0.06026897 -0.27855975
O 5.45765299 -2.22618692 -0.35838510
H 6.42525449 -2.18908405 -0.45590390
F -2.06173798 -2.91667724 0.09780189
F -1.03151403 1.73920136 0.03042374
H 0.49655972 -3.27893498 0.11258733
O 2.98458261 -3.11425118 0.07683068
H 3.29920016 -3.34319116 0.95444238
H -3.23326375 0.39537580 -0.68210253
H -3.58366184 -0.65829246 0.55463712

b5

G_{corr}[M062X/6-31G(d)] = 0.251774 a. u.
E[M062X/6-311G(2d)] = -1170.117466 a. u.
G = G_{corr} + E = -1169.865692 a. u.
C -1.12164243 -1.95875082 0.11515837
C 0.19958263 -2.23594898 0.12021027
C 1.24648593 -1.18152099 0.09383543
C 0.82901658 0.11003098 0.08842836
C -0.63175057 0.45072850 0.07923400

C -1.55940576 -0.52420428 0.09419445
C 2.68290829 -1.71731412 0.02723781
C 3.03077082 0.70304023 -0.02494842
C 3.57138623 -0.69455323 -0.09178776
H 3.78372926 1.47893129 -0.10214679
C -3.90624655 -0.92131709 0.75513843
C -3.39515080 0.62079709 -1.05571179
C -5.06847386 -0.01206022 1.16481396
H -3.47496619 -1.39810425 1.63934356
C -4.57506166 1.49395041 -0.62488288
H -2.60301393 1.24484141 -1.47271274
H -4.70180679 0.69946128 1.91499493
H -4.19209477 2.25464850 0.06694742
N -2.88542593 -0.08897458 0.12065121
N -5.63050938 0.75338905 0.05712261
N 1.77954113 1.21187170 0.11436132
C 5.03439193 -0.86498000 -0.24495759
O 5.81971706 0.06026897 -0.27855975
O 5.45765299 -2.22618692 -0.35838510
H 6.42525449 -2.18908405 -0.45590390
F -2.06173798 -2.91667724 0.09780189
F -1.03151403 1.73920136 0.03042374
C -6.44723546 -0.04480941 -0.84313770
H -6.89884612 0.61522052 -1.59039536
H -7.25601192 -0.51020461 -0.27130330
H -5.91101507 -0.84471491 -1.38047332
H -5.00594202 2.00531569 -1.49252266
H -3.70109530 -0.09963534 -1.83328152
H -5.86646602 -0.60769626 1.62155105
H -4.25722246 -1.71877359 0.08303800
H 0.49655972 -3.27893498 0.11258733
O 2.98458261 -3.11425118 0.07683068
H 3.29920016 -3.34319116 0.95444238

b5'

G_{corr}[M062X/6-31G(d)] = 0.251696 a. u.
E[M062X/6-311G(2d)] = -1170.114982 a. u.
G = G_{corr} + E = -1169.863286 a. u.

C -1.12164243 -1.95875082 0.11515837
C 0.19958263 -2.23594898 0.12021027
C 1.24648593 -1.18152099 0.09383543
C 0.82901658 0.11003098 0.08842836
C -0.63175057 0.45072850 0.07923400
C -1.55940576 -0.52420428 0.09419445
C 2.61268276 -1.79815341 0.03664562
C 3.13406477 0.66103419 -0.03644899
C 3.55002659 -0.63933450 -0.08914695
H 3.88702321 1.43692525 -0.11364736
C -3.90624655 -0.92131709 0.75513843
C -3.39515080 0.62079709 -1.05571179

C -5.06847386 -0.01206022 1.16481396
H -3.47496619 -1.39810425 1.63934356
C -4.57506166 1.49395041 -0.62488288
H -2.60301393 1.24484141 -1.47271274
H -4.70180679 0.69946128 1.91499493
H -4.19209477 2.25464850 0.06694742
N -2.88542593 -0.08897458 0.12065121
N -5.63050938 0.75338905 0.05712261
N 1.77954113 1.21187170 0.11436132
C 5.01303229 -0.80976127 -0.24231678
O 5.79835742 0.11548770 -0.27591894
O 5.43629335 -2.17096819 -0.35574429
H 6.40389485 -2.13386532 -0.45326309
F -2.06173798 -2.91667724 0.09780189
F -1.03151403 1.73920136 0.03042374
O 2.86993685 -2.98939754 0.07893616
C -6.44723546 -0.04480941 -0.84313770
H -6.89884612 0.61522052 -1.59039536
H -7.25601192 -0.51020461 -0.27130330
H -5.91101507 -0.84471491 -1.38047332
H -5.00594202 2.00531569 -1.49252266
H -3.70109530 -0.09963534 -1.83328152
H -5.86646602 -0.60769626 1.62155105
H -4.25722246 -1.71877359 0.08303800
H 0.49655972 -3.27893498 0.11258733
H 1.57448449 2.17893050 0.26518922

c1

G_{corr}[M062X/6-31G(d)] = 0.290785 a. u.
E[M062X/6-311G(2d)] = -1347.947723 a. u.
G = G_{corr} + E = -1347.656938 a. u.

C -1.05804500 -2.21068182 -0.06473129
C 0.25811488 -2.43805836 0.04307906
C 1.16399545 -1.24252349 0.05184134
C 0.73998365 0.03723296 -0.01842545
C -0.73609865 0.24673286 -0.18362483
C -1.56204945 -0.80421246 -0.19823585
C 2.61656658 -1.68979109 0.12063326
C 3.00223901 0.76028175 0.00161050
C 3.51656230 -0.49476649 0.01143655
H 3.69225368 1.59532652 -0.05188446
C -3.95874836 -1.18520161 0.46523260
C -3.53350381 0.38085184 -1.31114795
C -4.59557070 0.00578633 1.32051095
H -3.49161549 -1.90369547 1.13660889
C -4.21676818 1.54378244 -0.46386738
H -2.75954242 0.79068725 -1.95720053
H -3.78758238 0.45461589 1.90596932
H -3.42808555 2.05720983 0.08889551
N -3.00089347 -0.60363039 -0.42272353

N -5.16051962 0.96136356 0.43199305
N 1.61600909 1.21543008 0.05458804
C 1.18666988 2.61734869 0.24282297
C 2.28689847 3.54608122 0.71274758
H 0.40018803 2.62588932 1.00116920
H 0.77389516 3.00649641 -0.69166871
H 1.83352249 4.50101184 0.99451278
H 2.82763830 3.13878252 1.57343808
C 5.00429309 -0.54859309 -0.05979323
O 5.69700426 0.44308704 -0.05122863
O 5.52430986 -1.87749259 -0.15203569
H 6.49380819 -1.79309645 -0.19870380
F -1.93717950 -3.21461387 -0.10923972
F -1.24989949 1.48649367 -0.32796877
F 3.17815695 3.77626770 -0.31146662
O 2.94629492 -2.85345859 0.23364681
C -6.49629664 0.70263966 -0.07769673
H -6.89580546 1.61387663 -0.52589495
H -7.14662965 0.39628054 0.74278048
H -6.49270627 -0.09229441 -0.84206350
H -4.70834045 2.23697925 -1.14908800
H -4.32335935 -0.07625785 -1.91971499
H -5.35401011 -0.41145204 1.98597297
H -4.76744863 -1.66559246 -0.09684648
H 0.65544411 -3.44667778 0.09977195

c2

G_{corr}[M062X/6-31G(d)] = 0.308911 a. u.
E[M062X/6-311G(2d)] = -1423.774387 a. u.
G = G_{corr} + E = -1423.465476 a. u.

C -1.02418453 -2.28223014 -0.05715791
C 0.30537832 -2.49077711 0.04900614
C 1.16399545 -1.24252349 0.05184134
C 0.73998365 0.03723296 -0.01842545
C -0.73609865 0.24673286 -0.18362483
C -1.66291996 -0.93256077 -0.20002024
C 2.61656658 -1.68979109 0.12063326
C 3.00223901 0.76028175 0.00161050
C 3.51656230 -0.49476649 0.01143655
H 3.69225368 1.59532652 -0.05188446
C -4.05961887 -1.31354992 0.46344821
C -3.63437432 0.25250353 -1.31293234
C -4.69644121 -0.12256198 1.31872656
H -3.59248600 -2.03204378 1.13482450
C -4.31763869 1.41543413 -0.46565177
H -2.86041293 0.66233894 -1.95898492
H -3.88845289 0.32626758 1.90418493
H -3.52895606 1.92886152 0.08711112
N -3.10176398 -0.73197870 -0.42450792
N -5.26139013 0.83301525 0.43020866

N 1.61600909 1.21543008 0.05458804
C 1.18666988 2.61734869 0.24282297
C 2.28689847 3.54608122 0.71274758
H 0.40018803 2.62588932 1.00116920
H 0.77389516 3.00649641 -0.69166871
H 1.83352249 4.50101184 0.99451278
H 2.82763830 3.13878252 1.57343808
C 5.00429309 -0.54859309 -0.05979323
O 5.69700426 0.44308704 -0.05122863
O 5.52430986 -1.87749259 -0.15203569
H 6.49380819 -1.79309645 -0.19870380
F -1.90331903 -3.28616219 -0.10166634
F -0.73061600 0.98641152 -1.62489005
F 3.17815695 3.77626770 -0.31146662
O 2.94629492 -2.85345859 0.23364681
C -6.59716715 0.57429135 -0.07948112
H -6.99667597 1.48552832 -0.52767934
H -7.24750016 0.26793223 0.74099609
H -6.59357678 -0.22064272 -0.84384789
H -4.80921096 2.10863094 -1.15087239
H -4.42422986 -0.20460616 -1.92149938
H -5.45488062 -0.53980035 1.98418858
H -4.86831914 -1.79394077 -0.09863087
H 0.70270755 -3.49939653 0.10569903
O -1.40714346 1.58652683 0.72033921
H -1.67654181 2.47521691 0.87345233

c3

G_{corr}[M062X/6-31G(d)] = 0.308438 a. u.
E[M062X/6-311G(2d)] = -1323.915863 a. u.
G = G_{corr} + E = -1323.607425 a. u.

C -1.05804500 -2.21068182 -0.06473129
C 0.25811488 -2.43805836 0.04307906
C 1.16399545 -1.24252349 0.05184134
C 0.73998365 0.03723296 -0.01842545
C -0.73609865 0.24673286 -0.18362483
C -1.56204945 -0.80421246 -0.19823585
C 2.61656658 -1.68979109 0.12063326
C 3.00223901 0.76028175 0.00161050
C 3.51656230 -0.49476649 0.01143655
H 3.69225368 1.59532652 -0.05188446
C -3.95874836 -1.18520161 0.46523260
C -3.53350381 0.38085184 -1.31114795
C -4.59557070 0.00578633 1.32051095
H -3.49161549 -1.90369547 1.13660889
C -4.21676818 1.54378244 -0.46386738
H -2.75954242 0.79068725 -1.95720053
H -3.78758238 0.45461589 1.90596932
H -3.42808555 2.05720983 0.08889551
N -3.00089347 -0.60363039 -0.42272353

N -5.16051962 0.96136356 0.43199305
N 1.61600909 1.21543008 0.05458804
C 1.18666988 2.61734869 0.24282297
C 2.28689847 3.54608122 0.71274758
H 0.40018803 2.62588932 1.00116920
H 0.77389516 3.00649641 -0.69166871
H 1.83352249 4.50101184 0.99451278
H 2.82763830 3.13878252 1.57343808
C 5.00429309 -0.54859309 -0.05979323
O 5.69700426 0.44308704 -0.05122863
O 5.52430986 -1.87749259 -0.15203569
H 6.49380819 -1.79309645 -0.19870380
F -1.93717950 -3.21461387 -0.10923972
F 3.17815695 3.77626770 -0.31146662
O 2.94629492 -2.85345859 0.23364681
C -6.49629664 0.70263966 -0.07769673
H -6.89580546 1.61387663 -0.52589495
H -7.14662965 0.39628054 0.74278048
H -6.49270627 -0.09229441 -0.84206350
H -4.70834045 2.23697925 -1.14908800
H -4.32335935 -0.07625785 -1.91971499
H -5.35401011 -0.41145204 1.98597297
H -4.76744863 -1.66559246 -0.09684648
H 0.65544411 -3.44667778 0.09977195
O -1.28044652 1.56020123 -0.33655046
H -1.13072768 1.86665595 -1.23391870

c4

G_{corr}[M062X/6-31G(d)] = 0.296770 a. u.
E[M062X/6-311G(2d)] = -1347.694550 a. u.
G = G_{corr} + E = -1347.39778 a. u.

C -2.92465514 -0.74107055 0.10836021
C -1.75299245 -1.45252126 0.10837495
C -0.45445202 -0.68187688 0.10837406
C -0.40186642 0.67206517 0.10873561
C -1.69674605 1.42922216 0.10889809
C -2.89523840 0.75864096 0.10860809
C 0.81657777 -1.47842142 0.10809206
C 2.11444629 0.65339136 0.10858089
C 2.09192783 -0.71642348 0.10810899
H 3.08697140 1.16731042 0.10840289
C -4.78678282 1.85628395 1.43555931
C -4.78723546 1.85683396 -1.21090378
C -6.07948229 2.60553470 1.30307774
H -4.31132216 1.57925026 2.38762616
C -6.08438597 2.61009386 -1.21065213
H -4.31180929 1.58127971 -2.16335789
H -6.55536610 2.88082948 2.25546627
H -6.55958885 2.88655960 -2.16313475
N -4.22721517 1.53158200 0.10875089

N -6.77151135 3.00808235 0.07021474
N 0.86193108 1.42287532 0.10886690
C 0.87828760 2.89278428 0.10921424
C 2.28738591 3.40775218 0.10946992
H 0.32758852 3.24747616 1.01416713
H 0.32769045 3.24792391 -0.79561781
H 2.30026523 4.52471617 0.10967391
H 2.83768592 3.05233890 1.01441688
C 3.42397358 -1.48924561 0.10785418
O 4.14312496 -1.82824485 1.04671658
O 3.84533703 -1.84358172 -1.21191806
H 4.68603416 -2.32895307 -1.18356587
F -4.11826404 -1.37178268 0.10826203
F -1.65735532 2.77864736 0.10899870
F 2.95263600 2.97869689 -0.98408112
O 0.78076132 -2.70789984 0.10811442
C -8.04320579 3.74543753 0.07097795
H -8.07336130 4.38965784 -0.84120974
H -8.06020365 4.41051969 0.96840719
H -8.87256690 3.06951590 0.08476556
H -6.70229044 1.71797617 -1.10857322
H -5.25308713 0.87187431 -1.18029792
H -6.72728422 1.74094588 1.44778030
H -4.79310769 0.78514610 1.63736048
H -1.90732759 -2.50521000 -0.12853657

c5

G_{corr}[M062X/6-31G(d)] = 0.286521 a. u.
E[M062X/6-311G(2d)] = -1347.069139 a. u.
G = G_{corr} + E = -1346.782618 a. u.

C -1.05804500 -2.21068182 -0.06473129
C 0.25811488 -2.43805836 0.04307906
C 1.16399545 -1.24252349 0.05184134
C 0.73998365 0.03723296 -0.01842545
C -0.73609865 0.24673286 -0.18362483
C -1.56204945 -0.80421246 -0.19823585
C 2.61656658 -1.68979109 0.12063326
C 3.00223901 0.76028175 0.00161050
C 3.51656230 -0.49476649 0.01143655
H 3.69225368 1.59532652 -0.05188446
C -3.95874836 -1.18520161 0.46523260
C -3.53350381 0.38085184 -1.31114795
C -4.59557070 0.00578633 1.32051095
C -4.21676818 1.54378244 -0.46386738
H -2.75954242 0.79068725 -1.95720053
H -3.78758238 0.45461589 1.90596932
H -3.42808555 2.05720983 0.08889551
N -3.00089347 -0.60363039 -0.42272353
N -5.16051962 0.96136356 0.43199305
N 1.61600909 1.21543008 0.05458804

C 1.18666988 2.61734869 0.24282297
C 2.28689847 3.54608122 0.71274758
H 0.40018803 2.62588932 1.00116920
H 0.77389516 3.00649641 -0.69166871
H 1.83352249 4.50101184 0.99451278
H 2.82763830 3.13878252 1.57343808
C 5.00429309 -0.54859309 -0.05979323
O 5.69700426 0.44308704 -0.05122863
O 5.52430986 -1.87749259 -0.15203569
H 6.49380819 -1.79309645 -0.19870380
F -1.93717950 -3.21461387 -0.10923972
F -1.24989949 1.48649367 -0.32796877
F 3.17815695 3.77626770 -0.31146662
O 2.94629492 -2.85345859 0.23364681
C -6.49629664 0.70263966 -0.07769673
H -6.89580546 1.61387663 -0.52589495
H -7.14662965 0.39628054 0.74278048
H -6.49270627 -0.09229441 -0.84206350
H -4.70834045 2.23697925 -1.14908800
H -4.32335935 -0.07625785 -1.91971499
H -5.35401011 -0.41145204 1.98597297
H -4.76744863 -1.66559246 -0.09684648
H 0.65544411 -3.44667778 0.09977195

c6
G_{corr}[M062X/6-31G(d)] = 0.286520 a. u.
E[M062X/6-311G(2d)] = -1347.069138 a. u.
G = G_{corr} + E = -1346.782618 a. u.
C -1.05804500 -2.21068182 -0.06473129
C 0.25811488 -2.43805836 0.04307906
C 1.16399545 -1.24252349 0.05184134
C 0.73998365 0.03723296 -0.01842545
C -0.73609865 0.24673286 -0.18362483
C -1.56204945 -0.80421246 -0.19823585
C 2.61656658 -1.68979109 0.12063326
C 3.00223901 0.76028175 0.00161050
C 3.51656230 -0.49476649 0.01143655
H 3.69225368 1.59532652 -0.05188446
C -3.93238369 -1.05342388 0.46023274
C -3.53350381 0.38085184 -1.31114795
C -4.59557070 0.00578633 1.32051095
C -4.21676818 1.54378244 -0.46386738
H -2.75954242 0.79068725 -1.95720053
H -3.78758238 0.45461589 1.90596932
H -3.42808555 2.05720983 0.08889551
N -3.00089347 -0.60363039 -0.42272353
N -5.16051962 0.96136356 0.43199305
N 1.61600909 1.21543008 0.05458804
C 1.18666988 2.61734869 0.24282297
C 2.28689847 3.54608122 0.71274758

H 0.40018803 2.62588932 1.00116920
H 0.77389516 3.00649641 -0.69166871
H 1.83352249 4.50101184 0.99451278
H 2.82763830 3.13878252 1.57343808
C 5.00429309 -0.54859309 -0.05979323
O 5.69700426 0.44308704 -0.05122863
O 5.52430986 -1.87749259 -0.15203569
H 6.49380819 -1.79309645 -0.19870380
F -1.93717950 -3.21461387 -0.10923972
F -1.24989949 1.48649367 -0.32796877
F 3.17815695 3.77626770 -0.31146662
O 2.94629492 -2.85345859 0.23364681
C -6.49629664 0.70263966 -0.07769673
H -6.89580546 1.61387663 -0.52589495
H -7.14662965 0.39628054 0.74278048
H -6.49270627 -0.09229441 -0.84206350
H -4.70834045 2.23697925 -1.14908800
H -4.32335935 -0.07625785 -1.91971499
H -5.35401011 -0.41145204 1.98597297
H -4.74108396 -1.53381473 -0.10184634
H 0.65544411 -3.44667778 0.09977195

c7

G_{corr}[M062X/6-31G(d)] = 0.315874 a. u.
E[M062X/6-311G(2d)] = -1423.514815 a. u.
G = G_{corr} + E = -1423.198941 a. u.
C -1.07716192 -2.17834670 -0.09479724
C 0.23899796 -2.40572324 0.01301311
C 1.14487853 -1.21018837 0.02177539
C 0.72086673 0.06956808 -0.04849140
C -0.75521557 0.27906798 -0.21369078
C -1.58116637 -0.77187734 -0.22830180
C 2.59744966 -1.65745597 0.09056731
C 2.98312209 0.79261687 -0.02845545
C 3.49744538 -0.46243137 -0.01862940
H 3.67313676 1.62766164 -0.08195041
C -4.61386504 -1.44579208 1.04967432
C -3.56788382 0.44139941 -1.36667360
C -4.61083379 0.03399878 1.29505124
C -4.25114819 1.60433001 -0.51939303
H -2.79392243 0.85123482 -2.01272618
H -3.80284547 0.48282834 1.88050961
H -3.46246556 2.11775740 0.03336986
N -3.02001039 -0.57129527 -0.45278948
N -5.19489963 1.02191113 0.37646740
N 1.59689217 1.24776520 0.02452209
C 1.16755296 2.64968381 0.21275702
C 2.26778155 3.57841634 0.68268163
H 0.38107111 2.65822444 0.97110325
H 0.75477824 3.03883153 -0.72173466

H 1.81440557 4.53334696 0.96444683
H 2.80852138 3.17111764 1.54337213
C 4.98517617 -0.51625797 -0.08985918
O 5.67788734 0.47542216 -0.08129458
O 5.50519294 -1.84515747 -0.18210164
H 6.47469127 -1.76076133 -0.22876975
F -1.95629642 -3.18227875 -0.13930567
F -1.26901641 1.51882879 -0.35803472
F 3.15904003 3.80860282 -0.34153257
O 2.92717800 -2.82112347 0.20358086
C -6.53067665 0.76318723 -0.13322238
H -6.93018547 1.67442420 -0.58142060
H -7.18100966 0.45682811 0.68725483
H -6.52708628 -0.03174684 -0.89758915
H -4.74272046 2.29752682 -1.20461365
H -4.35773936 -0.01571028 -1.97524064
H -5.36927320 -0.38323959 1.96051326
H -5.42256531 -1.92618293 0.48759524
H 0.63632719 -3.41434266 0.06970600
H -3.22855958 -1.33531424 -1.06334843
O -4.03252140 -2.36809842 1.55106298
H -3.73170987 -3.27594998 1.63423732

c8

G_{corr}[M062X/6-31G(d)] = 0.295339 a. u.
E[M062X/6-311G(2d)] = -1423.149804 a. u.
G = G_{corr} + E = -1422.854465 a. u.
C -1.07716192 -2.17834670 -0.09479724
C 0.23899796 -2.40572324 0.01301311
C 1.14487853 -1.21018837 0.02177539
C 0.72086673 0.06956808 -0.04849140
C -0.75521557 0.27906798 -0.21369078
C -1.58116637 -0.77187734 -0.22830180
C 2.59744966 -1.65745597 0.09056731
C 2.98312209 0.79261687 -0.02845545
C 3.49744538 -0.46243137 -0.01862940
H 3.67313676 1.62766164 -0.08195041
C -4.61386504 -1.44579208 1.04967432
C -3.56788382 0.44139941 -1.36667360
C -4.61083379 0.03399878 1.29505124
C -4.25114819 1.60433001 -0.51939303
H -2.79392243 0.85123482 -2.01272618
H -3.80284547 0.48282834 1.88050961
H -3.46246556 2.11775740 0.03336986
N -3.02001039 -0.57129527 -0.45278948
N -5.19489963 1.02191113 0.37646740
N 1.59689217 1.24776520 0.02452209
C 1.16755296 2.64968381 0.21275702
C 2.26778155 3.57841634 0.68268163
H 0.38107111 2.65822444 0.97110325

H 0.75477824 3.03883153 -0.72173466
H 1.81440557 4.53334696 0.96444683
H 2.80852138 3.17111764 1.54337213
C 4.98517617 -0.51625797 -0.08985918
O 5.67788734 0.47542216 -0.08129458
O 5.50519294 -1.84515747 -0.18210164
H 6.47469127 -1.76076133 -0.22876975
F -1.95629642 -3.18227875 -0.13930567
F -1.26901641 1.51882879 -0.35803472
F 3.15904003 3.80860282 -0.34153257
O 2.92717800 -2.82112347 0.20358086
C -6.53067665 0.76318723 -0.13322238
H -6.93018547 1.67442420 -0.58142060
H -7.18100966 0.45682811 0.68725483
H -6.52708628 -0.03174684 -0.89758915
H -4.74272046 2.29752682 -1.20461365
H -4.35773936 -0.01571028 -1.97524064
H -5.36927320 -0.38323959 1.96051326
H -5.42256531 -1.92618293 0.48759524
H 0.63632719 -3.41434266 0.06970600
H -3.22855958 -1.33531424 -1.06334843
O -4.03252140 -2.36809842 1.55106298

c9

G_{corr}[M062X/6-31G(d)] = 0.250709 a. u.
E[M062X/6-311G(2d)] = -1169.858976 a. u.
G = G_{corr} + E = -1169.608267 a. u.
C -1.12164243 -1.95875082 0.11515837
C 0.19958263 -2.23594898 0.12021027
C 1.24648593 -1.18152099 0.09383543
C 0.82901658 0.11003098 0.08842836
C -0.63175057 0.45072850 0.07923400
C -1.55940576 -0.52420428 0.09419445
C 2.68290829 -1.71731412 0.02723781
C 3.03077082 0.70304023 -0.02494842
C 3.57138623 -0.69455323 -0.09178776
H 3.78372926 1.47893129 -0.10214679
C -3.90624655 -0.92131709 0.75513843
C -3.39515080 0.62079709 -1.05571179
C -5.06847386 -0.01206022 1.16481396
H -3.47496619 -1.39810425 1.63934356
C -4.57506166 1.49395041 -0.62488288
H -2.60301393 1.24484141 -1.47271274
H -4.70180679 0.69946128 1.91499493
H -4.19209477 2.25464850 0.06694742
N -2.88542593 -0.08897458 0.12065121
N -5.63050938 0.75338905 0.05712261
N 1.77954113 1.21187170 0.11436132
C 5.03439193 -0.86498000 -0.24495759
O 5.81971706 0.06026897 -0.27855975

O 5.45765299 -2.22618692 -0.35838510
H 6.42525449 -2.18908405 -0.45590390
F -2.06173798 -2.91667724 0.09780189
F -1.03151403 1.73920136 0.03042374
C -6.44723546 -0.04480941 -0.84313770
H -6.89884612 0.61522052 -1.59039536
H -7.25601192 -0.51020461 -0.27130330
H -5.91101507 -0.84471491 -1.38047332
H -5.00594202 2.00531569 -1.49252266
H -3.70109530 -0.09963534 -1.83328152
H -5.86646602 -0.60769626 1.62155105
H -4.25722246 -1.71877359 0.08303800
H 0.49655972 -3.27893498 0.11258733
O 2.98458261 -3.11425118 0.07683068
H 3.29920016 -3.34319116 0.95444238

c10

G_{corr}[M062X/6-31G(d)] = 0.239682 a. u.
E[M062X/6-311G(2d)] = -1169.274868 a. u.
G = G_{corr} + E = -1169.035186 a. u.
C -1.12164243 -1.95875082 0.11515837
C 0.19958263 -2.23594898 0.12021027
C 1.24648593 -1.18152099 0.09383543
C 0.82901658 0.11003098 0.08842836
C -0.63175057 0.45072850 0.07923400
C -1.55940576 -0.52420428 0.09419445
C 2.68290829 -1.71731412 0.02723781
C 3.03077082 0.70304023 -0.02494842
C 3.57138623 -0.69455323 -0.09178776
H 3.78372926 1.47893129 -0.10214679
C -3.90624655 -0.92131709 0.75513843
C -3.39515080 0.62079709 -1.05571179
C -5.06847386 -0.01206022 1.16481396
C -4.57506166 1.49395041 -0.62488288
H -2.60301393 1.24484141 -1.47271274
H -4.70180679 0.69946128 1.91499493
H -4.19209477 2.25464850 0.06694742
N -2.88542593 -0.08897458 0.12065121
N -5.63050938 0.75338905 0.05712261
N 1.77954113 1.21187170 0.11436132
C 5.03439193 -0.86498000 -0.24495759
O 5.81971706 0.06026897 -0.27855975
O 5.45765299 -2.22618692 -0.35838510
H 6.42525449 -2.18908405 -0.45590390
F -2.06173798 -2.91667724 0.09780189
F -1.03151403 1.73920136 0.03042374
C -6.44723546 -0.04480941 -0.84313770
H -6.89884612 0.61522052 -1.59039536
H -7.25601192 -0.51020461 -0.27130330
H -5.91101507 -0.84471491 -1.38047332

H -5.00594202 2.00531569 -1.49252266
H -3.70109530 -0.09963534 -1.83328152
H -5.86646602 -0.60769626 1.62155105
H -4.25722246 -1.71877359 0.08303800
H 0.49655972 -3.27893498 0.11258733
O 2.98458261 -3.11425118 0.07683068
H 3.29920016 -3.34319116 0.95444238

c11

G_{corr}[M062X/6-31G(d)] = 0.239680 a. u.
E[M062X/6-311G(2d)] = -1169.274868 a. u.
G = G_{corr} + E= -1169.035188 a. u.
C -1.12164243 -1.95875082 0.11515837
C 0.19958263 -2.23594898 0.12021027
C 1.24648593 -1.18152099 0.09383543
C 0.82901658 0.11003098 0.08842836
C -0.63175057 0.45072850 0.07923400
C -1.55940576 -0.52420428 0.09419445
C 2.68290829 -1.71731412 0.02723781
C 3.03077082 0.70304023 -0.02494842
C 3.57138623 -0.69455323 -0.09178776
H 3.78372926 1.47893129 -0.10214679
C -3.84406202 -0.82744216 0.70921772
C -3.39515080 0.62079709 -1.05571179
C -5.06847386 -0.01206022 1.16481396
C -4.57506166 1.49395041 -0.62488288
H -2.60301393 1.24484141 -1.47271274
H -4.70180679 0.69946128 1.91499493
H -4.19209477 2.25464850 0.06694742
N -2.88542593 -0.08897458 0.12065121
N -5.63050938 0.75338905 0.05712261
N 1.77954113 1.21187170 0.11436132
C 5.03439193 -0.86498000 -0.24495759
O 5.81971706 0.06026897 -0.27855975
O 5.45765299 -2.22618692 -0.35838510
H 6.42525449 -2.18908405 -0.45590390
F -2.06173798 -2.91667724 0.09780189
F -1.03151403 1.73920136 0.03042374
C -6.44723546 -0.04480941 -0.84313770
H -6.89884612 0.61522052 -1.59039536
H -7.25601192 -0.51020461 -0.27130330
H -5.91101507 -0.84471491 -1.38047332
H -5.00594202 2.00531569 -1.49252266
H -3.70109530 -0.09963534 -1.83328152
H -5.86646602 -0.60769626 1.62155105
H -4.19503793 -1.62489866 0.03711729
H 0.49655972 -3.27893498 0.11258733
O 2.98458261 -3.11425118 0.07683068
H 3.29920016 -3.34319116 0.95444238

c12

G_{corr}[M062X/6-31G(d)] = 0.268837 a. u.

E[M062X/6-311G(2d)] = -1245.699007 a. u.

G = G_{corr} + E = -1245.43017 a. u.

C -1.03219290 -1.61460312 0.40502509
C 0.27819677 -1.93228563 0.33826633
C 1.35620976 -0.91023386 0.29562013
C 0.98083618 0.39299893 0.35125604
C -0.46701519 0.77854169 0.42255262
C -1.42393913 -0.16738057 0.45065142
C 2.76992555 -1.48739681 0.14360271
C 3.19289505 0.92151851 0.15201102
C 3.68442552 -0.48902250 0.01500055
H 3.96607819 1.67604696 0.06376369
C -4.58956056 -0.98240867 1.35848061
C -3.33352933 1.09299629 -0.56864419
C -4.92102594 0.43818503 1.70782891
C -4.46327442 1.98714758 -0.05447554
H -2.54197741 1.70595821 -1.00283904
H -4.49700458 1.11269844 2.46196194
H -4.02411383 2.71223135 0.64219216
N -2.79266790 0.32907437 0.55888292
N -5.50911469 1.25704548 0.65312715
N 1.96659484 1.46363259 0.36693542
C 5.13243428 -0.69912567 -0.21307674
O 5.94478465 0.20215911 -0.25458517
O 5.50580074 -2.06815817 -0.38984640
H 6.46849447 -2.05757668 -0.53214187
F -2.00249552 -2.54207688 0.40199471
F -0.82683485 2.07960137 0.43391473
C -6.39226666 0.51477767 -0.23214795
H -6.85670106 1.21291808 -0.93562956
H -7.18809138 0.05565137 0.36247183
H -5.90777951 -0.28287003 -0.81963501
H -4.91742307 2.54015236 -0.88386437
H -3.75774913 0.72070102 -1.50113215
H -5.71565394 -0.14754257 2.18287844
H -3.70749726 -1.49965462 0.98096111
H 0.54068513 -2.98307098 0.28328174
O 3.02835049 -2.89382098 0.13424253
H 3.37600184 -3.16145060 0.98812363
H -3.06374674 -0.51180856 0.09044706
O -4.95838187 -1.51960364 0.35081380
H -5.18543639 -1.82755833 -0.52964640

c13

G_{corr}[M062X/6-31G(d)] = 0.250433 a. u.

E[M062X/6-311G(2d)] = -1245.333943 a. u.

$G = G_{corr} + E = -1245.08351$ a. u.

C -1.03219290 -1.61460312 0.40502509
C 0.27819677 -1.93228563 0.33826633
C 1.35620976 -0.91023386 0.29562013
C 0.98083618 0.39299893 0.35125604
C -0.46701519 0.77854169 0.42255262
C -1.42393913 -0.16738057 0.45065142
C 2.76992555 -1.48739681 0.14360271
C 3.19289505 0.92151851 0.15201102
C 3.68442552 -0.48902250 0.01500055
H 3.96607819 1.67604696 0.06376369
C -4.58956056 -0.98240867 1.35848061
C -3.33352933 1.09299629 -0.56864419
C -4.92102594 0.43818503 1.70782891
C -4.46327442 1.98714758 -0.05447554
H -2.54197741 1.70595821 -1.00283904
H -4.49700458 1.11269844 2.46196194
H -4.02411383 2.71223135 0.64219216
N -2.79266790 0.32907437 0.55888292
N -5.50911469 1.25704548 0.65312715
N 1.96659484 1.46363259 0.36693542
C 5.13243428 -0.69912567 -0.21307674
O 5.94478465 0.20215911 -0.25458517
O 5.50580074 -2.06815817 -0.38984640
H 6.46849447 -2.05757668 -0.53214187
F -2.00249552 -2.54207688 0.40199471
F -0.82683485 2.07960137 0.43391473
C -6.39226666 0.51477767 -0.23214795
H -6.85670106 1.21291808 -0.93562956
H -7.18809138 0.05565137 0.36247183
H -5.90777951 -0.28287003 -0.81963501
H -4.91742307 2.54015236 -0.88386437
H -3.75774913 0.72070102 -1.50113215
H -5.71565394 -0.14754257 2.18287844
H -3.70749726 -1.49965462 0.98096111
H 0.54068513 -2.98307098 0.28328174
O 3.02835049 -2.89382098 0.13424253
H 3.37600184 -3.16145060 0.98812363
H -3.07048219 -0.50157609 0.07634894
O -4.95838187 -1.51960364 0.35081380

c14

G_{corr}[M062X/6-31G(d)] = 0.249760 a. u.
E[M062X/6-311G(2d)] = -1245.055263 a. u.
$G = G_{corr} + E = -1244.805503$ a. u.

C -1.03219290 -1.61460312 0.40502509
C 0.27819677 -1.93228563 0.33826633

C 1.35620976 -0.91023386 0.29562013
C 0.98083618 0.39299893 0.35125604
C -0.46701519 0.77854169 0.42255262
C -1.42393913 -0.16738057 0.45065142
C 2.76992555 -1.48739681 0.14360271
C 3.19289505 0.92151851 0.15201102
C 3.68442552 -0.48902250 0.01500055
H 3.96607819 1.67604696 0.06376369
C -4.58956056 -0.98240867 1.35848061
C -3.33352933 1.09299629 -0.56864419
C -4.92102594 0.43818503 1.70782891
C -4.46327442 1.98714758 -0.05447554
H -2.54197741 1.70595821 -1.00283904
H -4.49700458 1.11269844 2.46196194
H -4.02411383 2.71223135 0.64219216
N -2.79266790 0.32907437 0.55888292
N -5.50911469 1.25704548 0.65312715
N 1.96659484 1.46363259 0.36693542
C 5.13243428 -0.69912567 -0.21307674
O 5.94478465 0.20215911 -0.25458517
O 5.50580074 -2.06815817 -0.38984640
H 6.46849447 -2.05757668 -0.53214187
F -2.00249552 -2.54207688 0.40199471
F -0.82683485 2.07960137 0.43391473
C -6.39226666 0.51477767 -0.23214795
H -6.85670106 1.21291808 -0.93562956
H -7.18809138 0.05565137 0.36247183
H -5.90777951 -0.28287003 -0.81963501
H -4.91742307 2.54015236 -0.88386437
H -3.75774913 0.72070102 -1.50113215
H -5.71565394 -0.14754257 2.18287844
H -3.70749726 -1.49965462 0.98096111
H 0.54068513 -2.98307098 0.28328174
O 3.02835049 -2.89382098 0.13424253
H 3.37600184 -3.16145060 0.98812363
H -3.07275832 -0.49801819 0.07157681
O -4.95838187 -1.51960364 0.35081380

c15

G_{corr}[M062X/6-31G(d)] = 0.238426 a. u.
E[M062X/6-311G(2d)] = -1244.487432 a. u.
G = G_{corr} + E = -1244.249006 a. u.
C -1.03219290 -1.61460312 0.40502509
C 0.27819677 -1.93228563 0.33826633
C 1.35620976 -0.91023386 0.29562013
C 0.98083618 0.39299893 0.35125604
C -0.46701519 0.77854169 0.42255262
C -1.42393913 -0.16738057 0.45065142
C 2.76992555 -1.48739681 0.14360271
C 3.19289505 0.92151851 0.15201102

C 3.68442552 -0.48902250 0.01500055
H 3.96607819 1.67604696 0.06376369
C -4.58956056 -0.98240867 1.35848061
C -3.33352933 1.09299629 -0.56864419
C -4.92102594 0.43818503 1.70782891
C -4.46327442 1.98714758 -0.05447554
H -2.54197741 1.70595821 -1.00283904
H -4.49700458 1.11269844 2.46196194
H -4.02411383 2.71223135 0.64219216
N -2.79266790 0.32907437 0.55888292
N -5.50911469 1.25704548 0.65312715
N 1.96659484 1.46363259 0.36693542
C 5.13243428 -0.69912567 -0.21307674
O 5.94478465 0.20215911 -0.25458517
O 5.50580074 -2.06815817 -0.38984640
H 6.46849447 -2.05757668 -0.53214187
F -2.00249552 -2.54207688 0.40199471
F -0.82683485 2.07960137 0.43391473
C -6.39226666 0.51477767 -0.23214795
H -6.85670106 1.21291808 -0.93562956
H -7.18809138 0.05565137 0.36247183
H -5.90777951 -0.28287003 -0.81963501
H -4.91742307 2.54015236 -0.88386437
H -5.71565394 -0.14754257 2.18287844
H -3.70749726 -1.49965462 0.98096111
H 0.54068513 -2.98307098 0.28328174
O 3.02835049 -2.89382098 0.13424253
H 3.37600184 -3.16145060 0.98812363
H -3.07350400 -0.49684139 0.07001254
O -4.95838187 -1.51960364 0.35081380

c16

G_{corr}[M062X/6-31G(d)] = 0.238419 a. u.
E[M062X/6-311G(2d)] = -1244.487432 a. u.
$G = G_{corr} + E$ = -1244.249013 a. u.
C -1.03219290 -1.61460312 0.40502509
C 0.27819677 -1.93228563 0.33826633
C 1.35620976 -0.91023386 0.29562013
C 0.98083618 0.39299893 0.35125604
C -0.46701519 0.77854169 0.42255262
C -1.42393913 -0.16738057 0.45065142
C 2.76992555 -1.48739681 0.14360271
C 3.19289505 0.92151851 0.15201102
C 3.68442552 -0.48902250 0.01500055
H 3.96607819 1.67604696 0.06376369
C -4.54327371 -1.04778501 1.45497423
C -3.28724248 1.02761995 -0.47215057
C -4.87473909 0.37280869 1.80432253
C -4.41698757 1.92177124 0.04201808
H -2.49569056 1.64058187 -0.90634542

H -4.45071773 1.04732210 2.55845556
H -3.97782698 2.64685501 0.73868578
N -2.79266790 0.32907437 0.55888292
N -5.46282784 1.19166914 0.74962077
N 1.96659484 1.46363259 0.36693542
C 5.13243428 -0.69912567 -0.21307674
O 5.94478465 0.20215911 -0.25458517
O 5.50580074 -2.06815817 -0.38984640
H 6.46849447 -2.05757668 -0.53214187
F -2.00249552 -2.54207688 0.40199471
F -0.82683485 2.07960137 0.43391473
C -6.34597981 0.44940133 -0.13565433
H -6.81041421 1.14754174 -0.83913594
H -7.14180453 -0.00972497 0.45896545
H -5.86149266 -0.34824637 -0.72314139
H -4.87113622 2.47477602 -0.78737075
H -5.66936709 -0.21291891 2.27937206
H -3.66121041 -1.56503096 1.07745473
H 0.54068513 -2.98307098 0.28328174
O 3.02835049 -2.89382098 0.13424253
H 3.37600184 -3.16145060 0.98812363
H -3.07350400 -0.49684139 0.07001254
O -4.91209502 -1.58497998 0.44730742

c17

G_{corr}[M062X/6-31G(d)] = 0.113029 a. u.
E[M062X/6-311G(2d)] = -919.509054 a. u.
G = G_{corr} + E = -919.396025 a. u.
C -1.12164243 -1.95875082 0.11515837
C 0.19958263 -2.23594898 0.12021027
C 1.24648593 -1.18152099 0.09383543
C 0.82901658 0.11003098 0.08842836
C -0.63175057 0.45072850 0.07923400
C -1.55940576 -0.52420428 0.09419445
C 2.68290829 -1.71731412 0.02723781
C 3.03077082 0.70304023 -0.02494842
C 3.57138623 -0.69455323 -0.09178776
H 3.78372926 1.47893129 -0.10214679
N -2.88542593 -0.08897458 0.12065121
N 1.77954113 1.21187170 0.11436132
C 5.03439193 -0.86498000 -0.24495759
O 5.81971706 0.06026897 -0.27855975
O 5.45765299 -2.22618692 -0.35838510
H 6.42525449 -2.18908405 -0.45590390
F -2.06173798 -2.91667724 0.09780189
F -1.03151403 1.73920136 0.03042374
H 0.49655972 -3.27893498 0.11258733
O 2.98458261 -3.11425118 0.07683068
H 3.29920016 -3.34319116 0.95444238
H -3.23326375 0.39537580 -0.68210253

H -3.58366184 -0.65829246 0.55463712

c18

G_{corr}[M062X/6-31G(d)] = 0.126503 a. u.
E[M062X/6-311G(2d)] = -995.273189 a. u.
G = G_{corr} + E = -995.146686 a. u.

C 2.27561663 1.50540808 0.17752363
C 0.92166365 1.74624310 0.27215727
C 0.07119509 0.62992852 0.29947957
C 0.59676664 -0.69866866 0.18031955
C 1.99676839 -0.88203507 0.10703585
C 2.85201396 0.24200337 0.07022230
C -1.33826699 0.77044717 0.39199968
C -1.48528706 -1.64181544 0.22930521
C -2.14071485 -0.37079374 0.36471808
H -2.12164719 -2.52484640 0.20050478
N 4.20411086 -0.03813889 0.15296530
N -0.20373568 -1.81493564 0.13887824
C -3.59182918 -0.38540515 0.44996668
O -4.32045727 -1.35354842 0.42417638
O -4.16287163 0.86678772 0.57070066
H -5.12001532 0.70704786 0.61737296
F 3.13353414 2.55951509 0.21347645
F 2.01858599 -1.19448804 1.69646881
H 0.54013742 2.75516768 0.35737771
O 1.39757654 -0.43177923 -1.47433659
H 1.23641457 -0.05396121 -2.34987964
H 4.39852596 -0.40770802 1.06160243
H 4.73223606 0.64218297 0.66114435
O -1.80211902 2.02948679 0.49987372
H -2.77155781 2.00301036 0.56446544

c19

G_{corr}[M062X/6-31G(d)] = 0.130463 a. u.
E[M062X/6-311G(2d)] = -895.470369 a. u.
G = G_{corr} + E = -895.339906 a. u.

C -1.12164243 -1.95875082 0.11515837
C 0.19958263 -2.23594898 0.12021027
C 1.24648593 -1.18152099 0.09383543
C 0.82901658 0.11003098 0.08842836
C -0.63175057 0.45072850 0.07923400
C -1.55940576 -0.52420428 0.09419445
C 2.68290829 -1.71731412 0.02723781
C 3.03077082 0.70304023 -0.02494842
C 3.57138623 -0.69455323 -0.09178776
H 3.78372926 1.47893129 -0.10214679
N -2.88542593 -0.08897458 0.12065121
N 1.77954113 1.21187170 0.11436132

C 5.03439193 -0.86498000 -0.24495759
O 5.81971706 0.06026897 -0.27855975
O 5.45765299 -2.22618692 -0.35838510
H 6.42525449 -2.18908405 -0.45590390
F -2.06173798 -2.91667724 0.09780189
H 0.49655972 -3.27893498 0.11258733
O 2.98458261 -3.11425118 0.07683068
H 3.29920016 -3.34319116 0.95444238
H -3.23326375 0.39537580 -0.68210253
H -3.58366184 -0.65829246 0.55463712
O -1.05522050 1.81560939 0.02752923
H -1.40178421 2.07627283 0.88400349

c20

G_{corr}[M062X/6-31G(d)] = 0.143245 a. u.
E[M062X/6-311G(2d)] = -871.451323 a. u.
G = G_{corr} + E = -871.308078 a. u.

C -1.12164243 -1.95875082 0.11515837
C 0.19958263 -2.23594898 0.12021027
C 1.24648593 -1.18152099 0.09383543
C 0.82901658 0.11003098 0.08842836
C -0.63175057 0.45072850 0.07923400
C -1.55940576 -0.52420428 0.09419445
C 2.68290829 -1.71731412 0.02723781
C 3.03077082 0.70304023 -0.02494842
C 3.57138623 -0.69455323 -0.09178776
H 3.78372926 1.47893129 -0.10214679
N -2.88542593 -0.08897458 0.12065121
N 1.77954113 1.21187170 0.11436132
C 5.03439193 -0.86498000 -0.24495759
O 5.81971706 0.06026897 -0.27855975
O 5.45765299 -2.22618692 -0.35838510
H 6.42525449 -2.18908405 -0.45590390
H 0.49655972 -3.27893498 0.11258733
O 2.98458261 -3.11425118 0.07683068
H 3.29920016 -3.34319116 0.95444238
H -3.23326375 0.39537580 -0.68210253
H -3.58366184 -0.65829246 0.55463712
O -1.05522050 1.81560939 0.02752923
H -1.40178421 2.07627283 0.88400349
O -2.12317787 -2.97928247 0.09666756
H -2.19501539 -3.37361821 0.96898556

c21

G_{corr}[M062X/6-31G(d)] = 0.047363 a. u.
E[M062X/6-311G(2d)] = -254.235046 a. u.
G = G_{corr} + E = -254.187683 a. u.

C -0.62590979 0.76419213 0.00000000
C 0.87434021 0.76419213 0.00000000

H -1.02102379 1.80912313 0.00000000
H -1.02130479 0.24180513 0.90486300
H 1.26936221 1.28673413 -0.90497800
H 1.26938021 1.28681613 0.90492100
O -1.13195860 0.09556963 -1.15834302
H -2.09173924 0.10577440 -1.14053944
F 1.35232295 -0.49835787 0.00007251

c22
G_{corr}[M062X/6-31G(d)] = 0.032220 a. u.
E[M062X/6-311G(2d)] = -253.567749 a. u.
G = G_{corr} + E= -253.535529 a. u.
C -0.62590979 0.76419213 0.00000000
C 0.87434021 0.76419213 0.00000000
H -1.02102379 1.80912313 0.00000000
H -1.02130479 0.24180513 0.90486300
H 1.26938021 1.28681613 0.90492100
O -1.13195860 0.09556963 -1.15834302
H -2.09173924 0.10577440 -1.14053944
F 1.35232295 -0.49835787 0.00007251

c23
G_{corr}[M062X/6-31G(d)] = 0.050355 a. u.
E[M062X/6-311G(2d)] = -329.459609 a. u.
G = G_{corr} + E= -329.409254 a. u.
C -0.62590979 0.76419213 0.00000000
C 0.87434021 0.76419213 0.00000000
H -1.02102379 1.80912313 0.00000000
H -1.02130479 0.24180513 0.90486300
H 1.26938021 1.28681613 0.90492100
O -1.13195860 0.09556963 -1.15834302
H -2.09173924 0.10577440 -1.14053944
F 1.35232295 -0.49835787 0.00007251
O 1.37997449 1.43305398 -1.15838586
H 2.01780755 2.09888584 -0.89110900

c24
G_{corr}[M062X/6-31G(d)] = 0.035273 a. u.
E[M062X/6-311G(2d)] = -229.000852 a. u.
G = G_{corr} + E= -228.965579 a. u.
C -0.62590979 0.76419213 0.00000000
C 0.87434021 0.76419213 0.00000000
H -1.02102379 1.80912313 0.00000000
H -1.02130479 0.24180513 0.90486300
H 1.26938021 1.28681613 0.90492100
O -1.13195860 0.09556963 -1.15834302
H -2.09173924 0.10577440 -1.14053944

O 1.29864870 1.32547480 -0.97207205

H_2
G_{corr}[M062X/6-31G(d)] = -0.001167 a. u.
E[M062X/6-311G(2d)] = -1.164709 a. u.
G = G_{corr} + E = -1.165876 a. u.
H -0.55489137 0.24456521 0.00000000
H -1.15489137 0.24456521 0.00000000

SiO_2
G_{corr}[M062X/6-31G(d)] = 0.042418 a. u.
E[M062X/6-311G(2d)] = -1913.124451 a. u.
G = G_{corr} + E = -1913.082033 a. u.
Si -0.32174222 0.07312734 -1.22894482
O -2.14750310 0.13669031 -1.12190769
O 0.25017025 1.18650263 -2.56393980
O 0.20862785 -1.63983354 -1.59414698
Si -2.87098144 -0.39933231 -2.71506771
Si -0.51485049 -2.17585615 -3.18730700
Si -0.47330809 0.65048001 -4.15709982
O -4.69674233 -0.33576934 -2.60803058
H -5.07627195 -0.61696153 -3.44378665
O 0.01551958 -3.88881703 -3.55250917
H -0.28450007 -4.47288276 -2.85218393
O -2.34061137 -2.11229318 -3.08026987
O -2.29906898 0.71404298 -4.05006269
O 0.05706198 -1.06248086 -4.52230198
O 0.09860437 1.76385530 -5.49209479
H -0.28092525 1.48266311 -6.32785087
O 0.40173613 0.60914996 0.36421520
H 0.10171647 0.02508423 1.06454043

SiO_2-F
G_{corr}[M062X/6-31G(d)] = 0.031087 a. u.
E[M062X/6-311G(2d)] = -1937.162311 a. u.
G = G_{corr} + E = -1937.131224 a. u.
Si -0.32174222 0.07312734 -1.22894482
O -2.14750310 0.13669031 -1.12190769
O 0.25017025 1.18650263 -2.56393980
O 0.20862785 -1.63983354 -1.59414698
Si -2.87098144 -0.39933231 -2.71506771
Si -0.51485049 -2.17585615 -3.18730700
Si -0.47330809 0.65048001 -4.15709982
O -4.69674233 -0.33576934 -2.60803058
H -5.07627195 -0.61696153 -3.44378665
O 0.01551958 -3.88881703 -3.55250917
H -0.28450007 -4.47288276 -2.85218393

O -2.34061137 -2.11229318 -3.08026987
O -2.29906898 0.71404298 -4.05006269
O 0.05706198 -1.06248086 -4.52230198
O 0.09860437 1.76385530 -5.49209479
H -0.28092525 1.48266311 -6.32785087
F 0.37010866 0.58571728 0.29456886
